# Phylogenetic Systematics, Biogeography, and Ecology of the Electric Fish Genus *Brachyhypopomus* (Ostariophysi: Gymnotiformes)

**DOI:** 10.1371/journal.pone.0161680

**Published:** 2016-10-13

**Authors:** William G. R. Crampton, Carlos David de Santana, Joseph C. Waddell, Nathan R. Lovejoy

**Affiliations:** 1 Department of Biology, University of Central Florida, Orlando, Florida, 32816–2368, United States of America; 2 Department of Biological Sciences, University of Toronto Scarborough, 1265 Military Trail, Toronto, Canada; Ecole normale superieure de Lyon, FRANCE

## Abstract

A species-level phylogenetic reconstruction of the Neotropical bluntnose knifefish genus *Brachyhypopomus* (Gymnotiformes, Hypopomidae) is presented, based on 60 morphological characters, approximately 1100 base pairs of the mitochondrial cytb gene, and approximately 1000 base pairs of the nuclear rag2 gene. The phylogeny includes 28 species of *Brachyhypopomus* and nine outgroup species from nine other gymnotiform genera, including seven in the superfamily Rhamphichthyoidea (Hypopomidae and Rhamphichthyidae). Parsimony and Bayesian total evidence phylogenetic analyses confirm the monophyly of the genus, and identify nine robust species groups. Homoplastic osteological characters associated with diminutive body size and occurrence in small stream habitats, including loss of squamation and simplifications of the skeleton, appear to mislead a phylogenetic analysis based on morphological characters alone–resulting in the incorrect placing of *Microsternarchus* + *Racenisia* in a position deeply nested within *Brachyhypopomus*. Consideration of geographical distribution in light of the total evidence phylogeny indicates an origin for *Brachyhypopomus* in Greater Amazonia (the superbasin comprising the Amazon, Orinoco and major Guiana drainages), with subsequent dispersal and vicariance in peripheral basins, including the La Plata, the São Francisco, and trans-Andean basins of northwest South America and Central America. The ancestral habitat of *Brachyhypopomus* likely resembled the normoxic, low-conductivity terra firme stream system occupied by many extant species, and the genus has subsequently occupied a wide range of terra firme and floodplain habitats including low- and high-conductivity systems, and normoxic and hypoxic systems. Adaptations for impedance matching to high conductivity, and/or for air breathing in hypoxic systems have attended these habitat transitions. Several species of *Brachyhypopomus* are eurytopic with respect to habitat occupancy and these generally exhibit wider geographical ranges than stenotopic species.

## Introduction

The weakly electric ‘bluntnose knifefish’ genus *Brachyhypopomus* (Gymnotiformes, Hypopomidae) is distributed in lowland freshwaters from southern Costa Rica to southern Uruguay [1–3]. *Brachyhypopomus* species are cryptically pigmented, nocturnally active predators of small aquatic invertebrates, and are small to moderate in size (not known to exceed 461 mm total length [3], and typically < 250 mm [3]). *Brachyhypopomus* species occur in lentic or slow-flowing habitats, including streams, swamps, and seasonal floodplains–where they often represent an exceptionally abundant component of local ichthyofaunas [4]. *Brachyhypopomus* generate species-typical, pulsed weakly electric organ discharges (EODs) of ca. 0.5–5 ms duration [5, 6]. These EODs comprise from one to four phases of alternating polarity and are generated at rates of <1–110 Hz [5, 6]. In combination with a cutaneous array of tuberous electroreceptors, EODs permit active electrolocation and electrocommunication [7]. The general biology of *Brachyhypopomus* is well known in comparison to other gymnotiform taxa [8], and one species, *B*. *gauderio* (formerly ascribed to *B*. *pinnicaudatus*), is a popular model species for studies of neurobiology, behavior, and ecology; for recent reviews see Gavassa et al. [9], Markham [10], Salazar et al. [11], Silva et al. [12], and Giora et al. [13].

*Brachyhypopomus* is currently represented by 13 valid species, which we list with authors in Table 1. A forthcoming publication by Crampton et al. [3] will confirm the validity of all 13 previously described species, and formally describe 15 additional new species. Because the names of the 15 species under description by Crampton et al. [3] are not yet available (sensu International Code of Zoological Nomenclature [ICZN]), we instead refer to them using the cheironyms listed in bold in Table 1. In accordance with Article 8.3 of the ICZN, 4^th^ edition, all nomenclatural acts in this paper are disclaimed for the purpose of zoological nomenclature.

**Table 1:** List of the 28 species of *Brachyhypopomus* included in this study, with authors and type localities.

Species	Author	Area of type locality
***Brachyhypopomus* sp. “alberti”**		Bolivia, Beni, Riberalta (rio Beni, Amazon dr.)
***Brachyhypopomus* sp. “arrayae”**		Bolivia, Beni, Riberalta (rio Beni, Amazon dr.)
***Brachyhypopomus* sp. “batesi”**		Brazil, Amazonas, Tefé (rio Tefé, Amazon dr.)
*Brachyhypopomus beebei*	(Schultz, 1944)	Venezuela, Monagas, Caripito (río San Juan, Orinoco dr.)
***Brachyhypopomus* sp. “belindae”**		Brazil, Amazonas, Tefé (rio Solimões, Amazon dr.)
***Brachyhypopomus* sp. “benjamini”**		Peru, Loreto, Jenaro Herrera (río Ucayali, Amazon dr.)
*Brachyhypopomus bennetti*	Sullivan, Zuanon, & Cox Fernandes, 2013	Brazil, Amazonas, Manaus (rio Solimões, Amazon dr.)
*Brachyhypopomus bombilla*	Loureiro & Silva, 2006	Uruguay, Rocha, Cebollati (Arroyo Cuatro Palmas, Laguna Merín dr.)
*Brachyhypopomus brevirostris*	(Steindachner, 1868)	Brazil, Rondônia (rio Guaporé, Amazon dr.)
*Brachyhypopomus bullocki*	Sullivan & Hopkins, 2009	Colombia, Meta, Puerto López (río Metica, Orinoco dr.)
***Brachyhypopomus* sp. “cunia”**		Brazil, Rondônia (rio Madeira, Amazon dr.)
*Brachyhypopomus diazi*	(Fernández-Yépez, 1972)	Venezuela, Carabobo, Morón (río Alpargatón, Salado dr.)
*Brachyhypopomus draco*	Giora, Malabarba & Crampton, 2009	Brazil, Rio Grande do Sul, Viamão (Lagoa Verde, Lagoa dos Patos dr.)
***Brachyhypopomus* sp. “flavipomus”**		Brazil, Amazonas, Alvarães (rio Solimões, Amazon dr.)
*Brachyhypopomus gauderio*	Giora & Malabarba, 2009	Brazil, Rio Grande do Sul, Palmares do Sul (Lagoa dos Patos dr.)
***Brachyhypopomus* sp. “hamiltoni”**		Brazil, Amazonas, Alvarães (rio Solimões, Amazon dr.)
***Brachyhypopomus* sp. “hendersoni”**		Brazil, Amazonas, Maraã (lago Amanã, Amazon dr.)
*Brachyhypopomus janeiroensis*	(Costa & Campos-da-Paz, 1992)	Brazil, Rio de Janeiro, Silva Jardim (córrego Salto d’Água, São João dr.)
*Brachyhypopomus jureiae*	Triques & Khamis, 2003	Brazil, São Paulo, Estação Ecológica Juréia (rio do Descalvado, Una do Prelado dr.)
***Brachyhypopomus* sp. “menezesi”**		Brazil, Bahia, São Marcelo (rio Sapão, São Francisco dr.)
*Brachyhypopomus occidentalis*	(Regan, 1914)	Colombia, Chocó (río Condoto, San Juan dr.)
***Brachyhypopomus* sp. “palenque”**		Ecuador, Los Ríos, Buena Fé (río Palenque, R. Guayas)
*Brachyhypopomus pinnicaudatus*	(Hopkins, 1991)	French Guiana, Cayenne, Kourou (Grand Pripris swamp, Kourou dr.)
***Brachyhypopomus* sp. “provenzanoi”**		Venezuela, Amazonas, San Fernando de Atabapo (R. Orinoco, Orinoco dr.)
***Brachyhypopomus* sp. “regani”**		Brazil, Amazonas, Alvarães (rio Solimões, Amazon dr.)
***Brachyhypopomus* sp. “sullivani”**		Peru, Loreto, Jenaro Herrera (río Ucayali, Amazon dr.)
***Brachyhypopomus* sp. “verdii”**		Peru, Loreto, Jenaro Herrera (río Ucayali, Amazon dr.)
*Brachyhypopomus walteri*	Sullivan, Zuanon & Cox Fernandes, 2013	Brazil, Amazonas, nr. Manaus (rio Solimões, Amazon dr.)

Species under description, [3], in bold, are listed with temporary cheironyms. dr. = drainage.

The genus *Brachyhypopomus* belongs to the family Hypopomidae Mago-Leccia, which sensu Maldonado-Ocampo et al. [14], contains five additional genera: *Akawaio*–comprising only *A*. *penak* Maldonado-Ocampo, López-Fernández, Taphorn, Bernard, Crampton, & Lovejoy [14]; *Hypopomus*–comprising only *H*. *artedi* (Kaup) [15]; and three genera in the tribe Microsternarchini: *Microsternarchus*–comprising *M*. *bilineatus* Fernández-Yépez [16] and *M*. *brevis* Fernandes, Nogueira, Williston, & Alves-Gomes [17], *Procerusternarchus*–comprising only *P*. *pixuna* Fernandes, Nogueira, & Alves-Gomes [18] and, *Racenisia*–comprising only *R*. *fimbriipinna* Mago-Leccia [19].

*Hypopygus* and *Steatogenys*, which form the tribe Steatogeni [20], have historically been placed in the Hypopomidae, but were transferred to the Rhamphichthyidae Regan (formerly comprising only *Gymnorhamphichthys*, *Iracema*, and *Rhamphichthys*) by Maldonado-Ocampo et al. [14], based on molecular phylogenetic evidence. The Hypopomidae and Rhamphichthyidae together constitute the superfamily Rhamphichthyoidea, the monophyly of which is supported by multiple morphology and DNA-based studies [14, 20–23].

Phylogenetic studies of species-level interrelationships in the Rhamphichthyoidea have to date considered only a subset of the species diversity in *Brachyhypopomus*. In an unpublished doctoral dissertation, Sullivan [24] provided a first species-level phylogenetic reconstruction of Rhamphichthyoidea based on mitochondrial sequence data from cytochrome *b* (cytb), 12S rRNA, and 16S rRNA genes. This analysis included 11 species of *Brachyhypopomus*: *B*. *beebei*, *B*. *bennetti*, *B*. *brevirostris*, *B*. *bullocki*, *B*. *diazi*, *B*. *janeiroensis*, *B*. *occidentalis*, *B*. *pinnicaudatus*, *B*. sp. “regani” (listed as *B*. *electropomus*), *B*. sp. “sullivani” (listed as *B*. *royeroi*), and *B*. *walteri*. Carvalho [22], in an unpublished doctoral dissertation, presented a rhamphichthyoid phylogeny based on a combination of morphological and mitochondrial sequence data (16S rRNA, cytb, and cytochrome oxidase I (COI) genes). This study included 14 species of *Brachyhypopomus*: *B*. *beebei*, *B*. *bennetti* (listed as *B*. sp. “ben”), *B*. *bombilla*, *B*. *brevirostris*, *B*. *bullocki*, *B*. *diazi*, *B*. *draco*, *B*. *gauderio*, *B*. *janeiroensis*, *B*. *occidentalis*, *B*. sp. “palenque” (listed as *B*. sp. “pal”), *B*. *pinnicaudatus*, *B*. sp. “regani” (listed as *B*. sp. “ele”), and *B*. *walteri* (listed as *B*. sp. “wal”). Maldonado-Ocampo et al. [14] presented a molecular phylogeny of Rhamphichthyoidea based on the nuclear rag2 gene, and mitochondrial cytb and COI genes, which included four species of *Brachyhypopomus*: *B*. *brevirostris*, *B*. *occidentalis*, *B*. sp. “palenque” (listed as *B*. sp. PALE), and *B*. *pinnicaudatus*. Tagliacollo et al. [23] presented a phylogeny of the entire order Gymnotiformes based on a combined molecular and morphological dataset, which included (with variable data completeness) nine species of *Brachyhypopomus*: *B*. *beebei*, *B*. *brevirostris*, *B*. *bullocki*, *B*. *diazi*, *B*. *draco*, *B*. *occidentalis*, *B*. sp. “palenque” (listed as B. sp. ‘pal’), and *B*. sp. “sullivani” (listed as B. sp. ‘roy’).

Here we provide a species-level phylogenetic reconstruction of *Brachyhypopomus*, summarize morphological diversity and character evolution in the genus, and discuss phylogenetic patterns of geographical distributions and habitat occupancy. Our analyses combine data from morphology and molecular data, and include all 13 previously-described species of *Brachyhypopomus* as well as the 15 new species under description by Crampton et al. [3] (Table 1). As outgroups we include single representatives of two non-rhamphichthyoid genera, and seven of the ten rhamphichthyoid genera other than *Brachyhypopomus* (all genera except *Akawaio* and *Procerusternarchus* in the Hypopomidae, and *Iracema* in the Rhamphichthyidae) (listed in Table 2). Although our analyses focused on obtaining a species-level phylogeny for *Brachyhypopomus*, the inclusion of multiple rhamphichthyoid genera as outgroups allowed us to comment on the monophyly of *Brachyhypopomus*, and on phylogenetic interrelationships among rhamphichthyoid genera.

**Table 2:** List of the nine outgroups species used for the phylogenetic analyses of *Brachyhypopomus*.

Family/Species	Author	Type Locality
**Gymnotidae:**		
*Gymnotus jonasi*	Albert & Crampton, 2001	Brazil, Amazonas, R. Solimões (Amazon dr.)
**Rhamphichthyidae:**		
*Rhamphichthys marmoratus*	Castelnau, 1855	Brazil, R. Araguaia (Amazon dr.).
*Gymnorhamphichthys rondoni*	(Miranda Ribeiro 1920)	Brazil, Amazonas, R. Cautário, (Amazon dr.).
**Hypopomidae:**		
*Hypopomus artedi*	(Kaup, 1856)	French Guiana, La Mana R. (La Mana dr.).
*Steatogenys duidae*	(La Monte, 1929)	Venezuela, Amazonas, Cerro Duida (Orinoco dr.).
*Hypopygus lepturus*	Hoedeman, 1962	Suriname, Maroni basin (Maroni dr.).
*Microsternarchus bilineatus*	Fernández-Yépez, 1968	Venezuela, Guárico, R. Guariquito (Orinoco dr.)
*Racenisia fimbriipinna*	Mago-Leccia, 1994	Venezuela, Amazonas, S. Fernando de Atabapo (Orinoco dr.).
**Sternopygidae:**		
*Sternopygus astrabes*	Mago-Leccia, 1994	Venezuela, Amazonas, Puerto Ayacucho (Orinoco dr.)

Taxa are ordered following the taxonomic scheme of Albert (2001). dr. = drainage.

## Materials and Methods

### Specimens and collections

We sampled muscle tissue for storage in 96–100% ethanol, or in a buffered solution of 20% DMSO and 0.25 M EDTA at pH 8, saturated with NaCl [25]. All specimens were subsequently fixed in 10% formalin, preserved in 70% EtOH, and assigned lot numbers in biodiversity collections. Specimens for which we collected tissue samples for DNA were euthanized in a 600 mgl^-1^ solution of eugenol (following the 2013 American Veterinary Medical Association Guidelines for the Euthanasia of Animals) until apnea and EOD cessation. Animal care protocols were approved by the Institutional Animal Care and Use Committee of the University of Central Florida (permits 06–33, 09-36W, 11-39W, and 12-31W).

Specimens from which DNA samples were analyzed were deposited along with tissue samples at the biodiversity collections listed in Table 3, and sequences were deposited in GenBank (also listed in Table 3). Field numbers beginning with the letters WC herein refer to specimens with EODs recorded by the Crampton Lab, and are provided to identify specimens from multi-individual lots. Specimens cleared and stained for osteological analyses are listed in S1 and S2 Appendices.

**Table 3:** Specimens used in molecular phylogenetic analyses.

Species	Voucher	No.	GenBank Accession No.		Locality
* *			RAG2	cytb	
***Brachyhypopomus*:**					
*B*. sp. “alberti” (UM*)	ANSP 197573 (WC03.250607)	7046	KX766540	KX766456	Bolivia, Beni, R. Beni, R. Amazonas dr.
*B*. sp. “alberti” (UM*)	CBF 10279 (WC04.250607)	7047	KX766541	KX766457	Bolivia, Beni, R. Beni, R. Amazonas dr.
*B*. sp. “batesi” (CA*)	MCP 45312 (WC03.201293)	2413	KX766542	KX766458	Brazil, Amazonas, R. Tefé, R. Amazonas dr.
*B*. sp. “batesi” (CA*)	MCP 45312 (WC06.201293)	2414	KX766543	KX766459	Brazil, Amazonas, R. Tefé, R. Amazonas dr.
*B*. *beebei* (UA)	UF 126247 (WC01.200902)	1943	KX766544	KX766460	Peru, Loreto, R. Ucayali, R. Amazonas dr.
*B*. *beebei* (CA)	MCP 45461 (WC06.210201)	2142	KX766545	KX766461	Brazil, Amazonas, R. Tefé, R. Amazonas dr.
*B*. *beebei* (GU)	UF 177358 (WC04.090307)	6967	KX766546	KX766462	Suriname, Marowijne, Cottica R., Commewijne R. dr.
*B*. sp. “belindae” (CA*)	MCP 45430 (WC03.150699)	2132	KX766547	KX766463	Brazil, Amazonas, R. Solimões, R. Amazonas dr.
*B*. sp. “belindae” (CA*)	MCP 45431 (WC06.230699)	2133	KX766548	KX766464	Brazil, Amazonas, R. Solimões, R. Amazonas dr.
*B*. sp. “benjamini” (UA*)	UF 148511 (WC18.090104)	2247	KX766549	KX766465	Peru, Loreto, R. Ucayali, R. Amazonas dr.
*B*. sp. “benjamini” (UA*)	UF 148512 (WC09.160104)	2275	KX766550	KX766466	Peru, Loreto, R. Ucayali, R. Amazonas dr.
*B*. *bennetti* (CA*)	MCP 45255 (WC01.150199)	2136	KX766551	KX766467	Brazil, Amazonas, R. Solimões, R. Amazonas dr.
*B*. *bennetti* (CA*)	MCP 45465 (WC02.080301)	2134	KX766552	KX766468	Brazil, Amazonas, R. Solimões, R. Amazonas dr.
*B*. *bennetti* (UA)	UF 126301	1914	KX766553	KX766469	Peru, Loreto, R. Ucayali, R. Amazonas dr.
*B*. *bennetti* (UA)	UF 126161 (WC10.200902)	1945	KX766554	KX766470	Peru, Loreto, R. Ucayali, R. Amazonas dr.
*B*. *bombilla* (UM)	UMSS 7035 (WC44.060707)	7042	KX766555	KX766471	Bolivia, Beni, R. Beni, R. Amazonas dr.
*B*. *bombilla* (UM)	UMSS 7038 (WC28.060707)	7040	KX766556	KX766472	Bolivia, Beni, R. Beni, R. Amazonas dr.
*B*. *bombilla* (UM)	UMSS 7040 (WC30.060707)	7037	KX766557	KX766473	Bolivia, Beni, R. Beni, R. Amazonas dr.
*B*. *bombilla* (PA)	UF 183773 (WC01.231106)	7097	KX766558	KX766474	Uruguay, Durazno, R. Negro, R. Uruguay dr.
*B*. *bombilla* (PA)	UF 183773 (WC02.231106)	7096	KX766559	KX766475	Uruguay, Durazno, R. Negro, R. Uruguay dr.
*B*. *bombilla* (PA)	UFRGS 10561	9103	KX766560	KX766476	Brazil, R. Grande do Sul, R. Uruguay dr.
*B*. *bombilla* (PA)	UFRGS 10561	9104	KX766561	KX766477	Brazil, R. Grande do Sul, R. Uruguay dr.
*B*. *brevirostris* (CA)	MCP 45623 (WC02.160201)	2139	KX766562	KX766478	Brazil, Amazonas, R. Tefé, R. Amazonas dr.
*B*. *brevirostris* (CA)	MCP 44758 (WC01.030597)	2140	KX766563	KX766479	Brazil, Amazonas, R. Tefé, R. Amazonas dr.
*B*. *brevirostris* (UA)	UF 116556	2617	GQ862536	GQ862588	Peru, Loreto, R. Nanay, R. Amazonas dr.
*B*. *brevirostris* (GU)	UF 177359 (WC17.090307)	7020	KX766564	KX766480	Suriname, Marowijne, Cottica R., Commewijne R. dr.
*B*. *brevirostris* (GU)	UF 177359 (WC18.090307)	7019	KF533301	KF533280	Suriname, Marowijne, Cottica R., Commewijne R. dr.
*B*. *brevirostris* (UM*)	UMSS 7024 (WC39.060707)	7038	KX766565	KX766481	Bolivia, Beni, R. Beni, R. Amazonas dr.
*B*. *brevirostris* (UM*)	UMSS 7033 (WC56.060707)	7044	KX766566	KX766482	Bolivia, Beni, R. Beni, R. Amazonas dr.
*B*. *bullocki* (OR)	UF 177348 (WC02.110304)	2364	KX766567	KX766483	Venezuela, Amazonas, R. Orinoco, R. Orinoco dr.
*B*. *bullocki* (OR)	UF 177348 (WC12.110304)	2362	KX766568	KX766484	Venezuela, Amazonas, R. Orinoco, R. Orinoco dr.
*B*. sp. “cunia” (CA*)	MCP 46937	9105	KX766569	KX766485	Brazil, Rondônia, R. Madeira, R. Amazonas dr.
*B*. sp. “cunia” (CA*)	MCP 46937	9106	KX766570	KX766486	Brazil, Rondônia, R. Madeira, R. Amazonas dr.
*B*. *diazi* (NW*)	UF 174333	2408	GQ862538	GQ862590	Venezuela, Carabobo, R. Alpargatón, R. Salado dr.
*B*. *diazi* (NW*)	UF 174333	2409	KX766571	KX766487	Venezuela, Carabobo, R. Alpargatón, R. Salado dr.
*B*. *diazi* (OR)	UF 174334 (WC01.210304)	305	GQ862537	GQ862589	Venezuela, Portuguesa, R. de las Marias, R. Orinoco dr.
*B*. *diazi* (OR)	UF 174334 (WC14.210304)	306	KX766572	KX766488	Venezuela, Portuguesa, R. de las Marias, R. Orinoco dr.
*B*. *draco* (SE)	UFRGS 14562	9100	KX766573	KX766489	Brazil, Rio Grande do Sul, R. Tramandaí dr.
*B*. *draco* (SE)	UFRGS 14562	9101	KX766574	KX766490	Brazil, Rio Grande do Sul, R. Tramandaí dr.
*B*. sp. “flavipomus” (CA*)	MCP 45265	2141	KX766575	KX766491	Brazil, Amazonas, R. Solimões, R. Amazonas dr.
*B*. sp. “flavipomus” (UA)	UF 129798	1926	KX766576	KX766492	Peru, Loreto, R. Ucayali, R. Amazonas dr.
*B*. sp. “flavipomus” (UA)	UF 129798	1928	KX766577	KX766493	Peru, Loreto, R. Ucayali, R. Amazonas dr.
*B*. *gauderio* (PA)	UF 177364 (WC02.130308)	7081	KX766578	KX766494	Argentina, Corrientes, Chaco region, R. Paraná dr.
*B*. *gauderio* (PA)	UF 177364 (WC03.130308)	7082	KX766579	KX766495	Argentina, Corrientes, Chaco region, R. Paraná dr.
*B*. *gauderio* (PA)	UF 183774	7094	KX766580	KX766496	Uruguay, Durazno, R. Negro, R. Uruguay dr.
*B*. *gauderio* (PA)	UF 183774	7095	KX766581	KX766497	Uruguay, Durazno, R. Negro, R. Uruguay dr.
*B*. *gauderio* (SE*)	UFRGS 14563	9102	KX766582	—	Brazil, R. Grande do Sul, R. Maquiné dr.
*B*. sp. “hamiltoni” (CA*)	MCP 45482 (WC05.080301)	2130	KX766583	KX766498	Brazil, Amazonas, R. Solimões, R. Amazonas dr.
*B*. sp. “hamiltoni” (CA*)	MCP 45681 (WC06.180898b)	7234	KX766584	KX766499	Brazil, Amazonas, R. Solimões, R. Amazonas dr.
*B*. sp. “hamiltoni” (CA*)	MCP 45302 (WC04.031298)	2125	KX766585	KX766500	Brazil, Amazonas, L. Amanã, R. Amazonas dr.
*B*. sp. “hamiltoni” (CA*)	MCP 45307 (WC01.160293b)	2206	KX766586	KX766501	Brazil, Amazonas, L. Amanã, R. Amazonas dr.
*B*. sp. “hendersoni” (CA*)	MCP 45274 (WC01.160201)	2143	KX766587	KX766502	Brazil, Amazonas, R. Tefé, R. Amazonas dr.
*B*. sp. “hendersoni” (CA*)	MCP 45397 (WC08.291298)	2240	KX766588	KX766503	Brazil, Amazonas, R. Tefé, R. Amazonas dr.
*B*. *janeiroensis* (BC*)	UF 183780	2954	KX766589	KX766504	Brazil, Rio de Janeiro, R. São João dr.
*B*. *janeiroensis* (BC*)	UF 183780	2955	KX766590	KX766505	Brazil, Rio de Janeiro, R. São João dr.
*B*. *jureiae* (BC*)	MZUSP 93118 (WC02.290706)	7108	KX766591	KX766506	Brazil, São Paulo, R. Momuna, R. Ribeira de Iguape dr.
*B*. *jureiae* (BC*)	MZUSP 100268 (WC02.160708)	7232	KX766592	KX766507	Brazil, São Paulo, R. Momuna, R. Ribeira de Iguape dr.
*B*. *occidentalis* (PS*)	IMCN 4523	8199	KX766593	KX766508	Colombia, Valle del Cauca, R. San Cipriano dr.
*B*. *occidentalis* (PS*)	IMCN 4523	8204	KX766594	KX766509	Colombia, Valle del Cauca, R. San Cipriano dr.
*B*. *occidentalis* (MA)	UF 183793	7159	KX766595	KX766510	Panama, Bocas del Toro, R. Cricamola dr.
*B*. *occidentalis* (MA)	UF 183793	7160	KX766596	KX766511	Panama, Bocas del Toro, R. Cricamola dr.
*B*. sp. “palenque” (PS*)	UF 148572 (WC02.160404)	2432	GQ862539	GQ862591	Ecuador, Los Ríos, R. Palenque, R. Guayas dr.
*B*. sp. “palenque” (PS*)	UF 148572 (WC03.160404)	2433	KX766597	KX766512	Ecuador, Los Ríos, R. Palenque, R. Guayas dr.
*B*. *pinnicaudatus* (CA)	MCP 45281 (WC01.080301)	2121	KF533303	KF533282	Brazil, Amazonas, R. Solimões, R. Amazonas dr.
*B*. *pinnicaudatus* (CA)	MCP 45433 (WC01.150699)	2122	KF533304	KF533283	Brazil, Amazonas, R. Solimões, R. Amazonas dr.
*B*. *pinnicaudatus* (UM)	UMSS 7030 (WC26.270607)	7051	KX766598	KX766513	Bolivia, Beni, R. Beni, R. Amazonas dr.
*B*. *pinnicaudatus* (UM)	UMSS 7031 (WC27.270607)	7035	KX766599	KX766514	Bolivia, Beni, R. Beni, R. Amazonas dr.
*B*. sp. “provenzanoi” (OR*)	MBUCV-V 35651 (WC30.150304)	2308	KX766600	KX766515	Venezuela, Amazonas, R. Orinoco, R. Orinoco dr.
*B*. sp. “provenzanoi” (OR*)	UF 177347 (WC07.110304)	2365	KX766601	KX766516	Venezuela, Amazonas, R. Orinoco, R. Orinoco dr.
*B*. sp. “regani” (CA*)	MCP 45284 (WC03.080301)	2118	KX766602	KX766517	Brazil, Amazonas, R. Solimões, R. Amazonas dr.
*B*. sp. “regani” (CA*)	MCP 45473 (WC02.100301)	2119	KX766603	KX766518	Brazil, Amazonas, R. Solimões, R. Amazonas dr.
*B*. sp. “regani” (UA)	UF 129816	1901	KX766604	KX766519	Peru, Loreto, R. Ucayali, rio Amazonas dr.
*B*. sp. “regani” (GU)	UF 177360 (WC06.090307)	6963	KX766605	KX766520	Suriname, Marowijne, Cottica R., Commewijne R. dr.
*B*. sp. “regani” (GU)	UF 177360 (WC12.090307)	6964	KX766606	KX766521	Suriname, Marowijne, Cottica R., Commewijne R. dr.
*B*. sp. “sullivani” (UM)	CBF 10254 (WC19.280607)	7036	KX766607	KX766522	Bolivia, Beni, R. Beni, R. Amazonas dr.
*B*. sp. “sullivani” (CA)	MCP 45464 (WC04.210201)	2123	KX766608	KX766523	Brazil, Amazonas, R. Tefé, R. Amazonas dr.
*B*. sp. “sullivani” (UA*)	UF 148515 (WC 04.130104)	2267	KX766609	—	Peru, Loreto, R. Ucayali, R. Amazonas dr.
*B*. sp. “sullivani” (UA*)	UF 148517 (WC 04.150104)	2274	KX766610	KX766524	Peru, Loreto, R. Ucayali, R. Amazonas dr.
*B*. sp. “sullivani” (UM)	UF 177341 (WC55.060707)	7039	KX766611	KX766525	Bolivia, Beni, R. Beni, R. Amazonas dr.
*B*. sp. “verdii” (UA*)	UF 148520 (WC24.090104)	2253	KX766612	KX766526	Peru, Loreto, R. Ucayali, R. Amazonas dr.
*B*. sp. “verdii” (UA*)	UF 148520 (WC25.090104)	2254	KX766613	KX766527	Peru, Loreto, R. Ucayali, R. Amazonas dr.
*B*. *walteri* (UM)	CBF 10256 (WC01.250607)	7045	KX766614	KX766528	Bolivia, Beni, R. Beni, R. Amazonas dr.
*B*. *walteri* (UM)	CBF 10257 (WC07.250607)	7048	KX766615	KX766529	Bolivia, Beni, R. Beni, R. Amazonas dr.
*B*. *walteri* (CA*)	MCP 45477 (WC04.200201)	2116	KX766616	KX766530	Brazil, Amazonas, R. Tefé, R. Amazonas dr.
*B*. *walteri* (CA*)	MCP 45478	2115	KX766617	KX766531	Brazil, Amazonas, R. Tefé, R. Amazonas dr.
***Gymnorhamphichthys*:**					
*G*. *rondoni*	MCP 46936	2153	KF533315	—	Brazil, Amazonas, R. Tefé, R. Amazonas dr.
*G*. *rondoni*	MCP 46936	2154	KF533316	—	Brazil, Amazonas, R. Tefé, R. Amazonas dr.
***Gymnotus*:**					
*G*. *jonasi*	UF 131410 (WC01.140503)	2471	GQ862568	GQ863620	Peru, Loreto, R. Ucayali, R. Amazonas dr.
*G*. *jonasi*	MZUSP 103220 (WC04.080301)	2016	GQ862567	GQ862619	Brazil, Amazonas, R. Tefé, R. Amazonas dr.
***Hypopomus*:**					
*H*. *artedi*	ANSP 179505	2232	GQ862585	GQ862637	Guyana, Cuyuni-Mazaruni, Essequibo R. dr.
*H*. *artedi*	AUM 35574	2233	KF533306	KF533285	Guyana, Cuyuni-Mazaruni, Essequibo R. dr.
***Hypopygus*:**					
*H*. *lepturus*	UF 176880	2438	KX766618	KX766532	Peru, Loreto, R. Ucayali, R. Amazonas dr.
*H*. *lepturus*	UF 176882 (WC06.050307)	7024	KX766619	KX766533	Suriname, Para, Suriname R. dr.
***Microsternarchus*:**					
*M*. *bilineatus*	MCP 45463	2138	KF533310	KF533291	Brazil, Amazonas, R. Tefé, R. Amazonas dr.
*M*. *bilineatus*	MCP 45480 (WC02.200201)	2137	—	KF533290	Brazil, Amazonas, R. Tefé, R. Amazonas dr.
***Racenisia***					
*R*. *fimbriipinna*	UF 177352 (WC05.130304)	2339	KF533311	KF533292	Venezuela, Amazonas, R. Orinoco, R. Orinoco dr.
*R*. *fimbriipinna*	UF 177352 (WC12.120304)	2340	KF533312	KF533293	Venezuela, Amazonas, R. Orinoco, R. Orinoco dr.
***Rhamphichthys*:**					
*R*. *marmoratus*	MCP 46932 (WC05.100299)	2156	KX766620	KX766534	Brazil, Amazonas, R. Japurá, R. Amazonas dr.
*R*. *marmoratus*	MCP 46929 (WC10.070201)	2155	KX766621	KX766535	Brazil, Amazonas, R. Japurá, R. Amazonas dr.
***Steatogenys*:**					
*S*. *duidae*	MCP 31958	2147	KX766622	KX766536	Brazil, Amazonas, R. Demini, R. Amazonas dr.
*S*. *duidae*	MCP 31960 (WC01.210201)	2146	KX766623	KX766537	Brazil, Amazonas, R. Tefé, R. Amazonas dr.
***Sternopygus*:**					
*S*. *astrabes*	MCP 32231 (WC16.240899)	2203	KX766624	KX766538	Brazil, Amazonas, R. Tefé, R. Amazonas dr.
*S*. *astrabes*	MCP 32235 (WC07.291000)	2204	KX766625	KX766539	Brazil, Amazonas, R. Tefé, R. Amazonas dr.

Species: specimens are ordered alpha-numerically by genus, species, and museum lot; drainage units are reported in parentheses as two letter codes (see ‘Geographic and ecological distributions‘ in Materials and Methods). *Brachyhypopomus* specimens marked with an asterisk are from the region of the type locality. Voucher: field numbers prefixed WC refer to specimen-specific electric signal recordings. No.: voucher numbers match the tissue numbers reported in the phylogenetic trees presented herein (Museum acronyms are listed in text).–: sequence not available. Locality: lot information is abbreviated.

Specimens subjected to morphological and molecular analyses in this study are deposited at the following biodiversity institutions: Academy of Natural Sciences (of Drexel University), Philadelphia, PA, USA (ANSP); Auburn University Natural History Museum, Auburn, AL, USA (AUM); Colección Boliviana de Fauna, Museo Nacional de Historia Natural, Instituto de Ecología, La Paz, Bolivia (CBF); Field Museum of Natural History, Zoology Department, Chicago, IL, USA (FMNH); Instituto Vallecaucano de Investigaciones Científicas, Cali, Colombia (IMCN); Instituto Nacional de Pesquisas da Amazônia, Manaus, Brazil, Amazonas (INPA); University of Kansas Biodiversity Institute, Lawrence, KS, USA (KU); Museo de Biología de la Universidad Central de Venezuela, Caracas, Venezuela (MBUCV-V); Museu de Ciências e Tecnologia, Pontifícia Universidade Católica do Rio Grande do Sul, Porto Alegre, Rio Grande do Sul, Brazil (MCP); Museu de Zoologia da Universidade de São Paulo, São Paulo, Brazil (MZUSP); Swedish Museum of Natural History, Stockholm, Sweden (NRM); Naturalis Biodiversity Center, Leiden, Netherlands (RMNH); Florida Museum of Natural History, University of Florida, Gainesville, FL, UA (UF); Universidade Federal do Rio Grande do Sul, Porto Alegre, Brazil (UFRGS); Universidad Mayor de San Simón, Facultad de Ciencias y Tecnologia, Centro de Biodiversidad, Zoología, Laboratorio de Ictiología, Cochabamba, Bolivia (UMSS); National Museum of Natural History, Smithsonian Institution, Washington, D.C. (USNM).

All field collections conducted by WGRC, CDS, JCW and NRL were authorized by the appropriate national and regional collection and export permits, including: Brazil–via Conselho Nacional de Desenvolvimento Científico e Tecnológico (Brasília) (Expedição Científica protocols); Instituto Brasileiro do Meio Ambiente e dos Recursos Naturais Renováveis or (after 2007) Instituto Chico Mendes de Conservação de Biodiversidade, Sistema de Autorização e Informação em Biodiversidade; Instituto de Desenvolvimento Sustentável Mamirauá. Peru–via Ministerio de la Produción and Universidad Nacional Mayor de San Marcos (Lima), and Dirección Regional de La Producción, Loreto (Iquitos). Panama–via Ministerio de Ambiente de Panamá (Ciudad de Panamá) and Smithsonian Tropical Research Institute (Ciudad de Panamá). Bolivia–via Universidad Mayor de San Simón Facultad de Ciencias y Tecnología (Cochabamba). Colombia–via Instituto de Investigación de Recursos Biológicos Alexander von Humboldt (Bogotá). Ecuador–via Fundación Wong (Guayaquil) and Centro Científico río Palenque. Suriname–via Anton de Kom University of Suriname, and Ministry of Agriculture, Animal Husbandry and Fisheries Department (Paramaribo). Uruguay–via Instituto de Investigaciones Biológicas Clemente Estable (Montevideo). Venezuela–via Universidad Nacional Experimental de los Llanos Occidentales Ezequiel Zamora (Barinas), and Ministerio Ambiental y Recursos Naturales (Caracas). No protected species were sampled. Tissue samples from Brazil, including those not collected by ourselves were received via formal Material Transfer Agreements, including MTA-002-2008-PUCRS (Museu de Ciências e Tecnologia, Pontifícia Universidade do Rio Grande do Sul/UCF), MTA-008-2011-MZUSP (Museu de Zoologia da Universidade de São Paulo/UCF), and MTA-001-2011-UFRGS (Universidade Federal do Rio Grande do Sul/UCF).

### Geographic and ecological distributions

The geographic ranges and habitat occupancy data presented herein for *Brachyhypopomus* species are based on the descriptions and redescriptions of all 28 species of *Brachyhypopomus* provided by Crampton et al. [3], which include distribution maps for 11,750 specimens from 2,642 georeferenced museum lots. Geographic and ecological distributions for outgroup taxa are based on original species descriptions in the literature, and on a recent review of gymnotiform ecology and biogeography [2].

To facilitate the presentation and analysis of biogeographical distributions, collection records listed in this paper are categorized into the following five geographical regions and 14 drainage subunits listed below. These drainage units represent basins, sub-basins, or groups of adjacent small basins selected to together summarize broad patterns of distribution in the genus.

**Region 1 (Greater Amazonia):** (**OR**) = Orinoco basin; (**GU**) = Caribbean drainages of the Guianas in Guyana, Suriname and French Guiana, including Oyapock; (**RN**) = rio Negro; (**UA**) = Upper Amazon–rio Amazonas and tributaries upstream of mouth of the rio Jutai; (**CA**) = Central Amazon–rio Amazonas and tributaries between mouths of the rio Jutai and rio Uatumã, including the lower rio Madeira (below the first rapids at Cachoeira de Santo Antônio, near Porto Velho), but excluding the rio Negro; (**LA**) = Lower Amazon–rio Amazonas and tributaries downstream of the mouth of the rio Uatumã, including rio Tocantins and coastal drainages of Amapá east of Oyapock, Pará, and Maranhão west of São Luiz; (**UM**) = Upper Madeira, upstream of Cachoeira de Santo Antônio. **Region 2 (La Plata–Lagoa dos Patos):** (PA) = La Plata drainages, i.e. rio Paraná-Paraguay, rio Uruguay, and small coastal drainages west of Punta del Este, Uruguay; (**SE**) = Atlantic coastal drainages of Uruguay east of Punta del Este, the Lagoa dos Patos-Merim systems, and the adjacent Maquiné and Tramandaí drainages of Rio Grande do Sul. **Region 3 (Brazilian coastal drainages):** (**BC**) = Atlantic coastal drainages–rio Ribeira de Iguape, São Paulo, and rio São João and rio Paraíba do Sul, Rio de Janeiro. **Region 4 (São Francisco drainage):** (**NE**) = middle and upper São Francisco. **Region 5 = (Trans-Andean drainages):** (**MA**) = Middle America–Atlantic and Pacific drainages of Panama to Darien (and parts of southern Costa Rica; (**PS**) = Pacific Slope–Pacific drainages of western Ecuador and Colombia, including Pacific drainages and the Atlantic Atrato drainage; (**NW**) = northwest South America–trans-Andean Caribbean drainages of NW South America, including Sinú, Magdalena, Lago Maracaibo, and Caribbean drainages north of the Andean coastal range.

### Osteological preparation and nomenclature

The osteology of representatives of 28 species of *Brachyhypopomus* and nine outgroup species was examined from cleared and stained (CS) specimens; see list of specimens in S1 and S2 Appendices. Specimens of reproductive size for each species were examined to avoid the inclusion of juvenile characters, unless stated otherwise. Radiographs served as supplementary sources of data for some species. Specimens were cleared and counterstained for cartilage and bone using the method outlined by Taylor & Van Dyke [26]. In some specimens with weak ossification, bones were stained with alizarin red in ethanol solution instead of KOH solution [27]. The pectoral girdle, suspensorium, and components of the head were removed following Weitzman [28]. Osteological nomenclature and homology follow de Santana & Vari [29] and Hilton et al. [30], except for lateral line system nomenclature, which follows Arratia & Huaquin [31].

### Morphology-based phylogenetic reconstruction

We applied parsimony analysis to a matrix of 60 morphological characters (Table 4) from 37 terminal taxa: 28 ingroup species of *Brachyhypopomus* (Table 1) and nine outgroup species (Table 2). The outgroup species match those in our molecular phylogenetic reconstruction and represent seven of the ten rhamphichthyoid genera outside *Brachyhypopomus* (*Gymnorhamphichthys*, *Hypopomus*, *Hypopygus*, *Microsternarchus*, *Racenisia*, *Rhamphichthys*, *Steatogenys*). Three genera, *Iracema*, *Akawaio*, and *Procerusternarchus* were not included because cleared and stained specimens and molecular data were not available. Characters were chosen primarily to elucidate inter-specific phylogenetic relationships within *Brachyhypopomus*. However, we included some autapomorphic (non parsimony-informative) characters in *Brachyhypopomus*, since these may be indicative of synapomorphies with the addition of newly-discovered taxa in the future. All characters were binary, except for character 48, which is multistate and coded as unordered.

**Table 4:** Matrix of 60 morphological characters for 28 species of *Brachyhypopomus* and nine outgroup taxa.

	**1**	**2**	**3**	**4**	**5**	**6**	**7**	**8**	**9**	**10**	**11**	**12**	**13**	**14**	**15**	**16**	**17**	**18**	**19**	**20**
**Ingroup:**																				
*Brachyhypopomus* sp. "alberti"	1	0	0	0	0	1	-	-	0	0	0	1	1	0	1	1	1	0	1	1
*Brachyhypopomus* sp. "arrayae"	1	0	0	0	0	1	-	-	0	0	0	1	1	0	1	1	1	0	1	1
*Brachyhypopomus* sp. "batesi"	1	0	0	0	0	0	0	0	0	0	0	1	1	0	1	0	-	0	0	-
*Brachyhypopomus beebei*	1	0	0	0	0	0	0	0	0	0	0	1	1	0	1	1	1	0	1	0
*Brachyhypopomus* sp. "belindae"	1	0	0	0	0	1	-	-	0	0	0	1	1	0	1	1	1	0	0	-
*Brachyhypopomus* sp. "benjamini"	1	0	0	0	0	0	0	0	0	0	0	1	1	0	1	0	-	0	0	-
*Brachyhypopomus bennetti*	1	0	1	0	0	0	0	0	0	0	0	1	1	0	1	1	0	0	1	0
*Brachyhypopomus bombilla*	1	0	0	0	0	0	0	0	0	0	0	1	1	0	1	0	-	0	1	0
*Brachyhypopomus brevirostris*	1	0	0	0	0	0	0	0	0	0	0	1	1	0	1	1	0	0	0	-
*Brachyhypopomus bullocki*	1	0	0	0	0	0	0	0	0	0	0	1	1	0	1	1	1	0	1	0
*Brachyhypopomus* sp. "cunia"	1	0	0	0	0	0	0	0	0	0	0	1	1	0	1	1	1	1	0	-
*Brachyhypopomus diazi*	1	0	0	0	0	0	0	0	0	0	0	1	1	0	1	1	0	0	0	-
*Brachyhypopomus draco*	1	0	0	0	0	0	0	0	0	0	0	1	1	0	1	1	0	0	0	-
*Brachyhypopomus sp*. *"flavipomus"*	1	0	0	0	0	0	0	1	1	0	0	1	1	0	1	1	1	0	0	-
*Brachyhypopomus gauderio*	1	0	0	0	0	0	0	0	0	0	0	1	1	0	1	1	1	0	1	0
*Brachyhypopomus* sp. "hamiltoni"	1	0	0	0	0	1	-	-	0	0	0	1	1	0	1	1	1	0	1	0
*Brachyhypopomus* sp. "hendersoni"	1	0	0	0	0	0	0	0	0	0	0	1	1	0	1	1	1	1	0	-
*Brachyhypopomus janeiroensis*	1	0	0	0	0	0	0	0	0	0	0	1	1	0	1	1	0	0	0	-
*Brachyhypopomus jureiae*	1	0	0	0	0	0	0	0	0	0	0	1	1	0	1	0	-	0	0	-
*Brachyhypopomus* sp. "menezesi"	1	0	0	0	0	0	0	0	0	0	0	1	1	0	1	0	-	0	1	0
*Brachyhypopomus occidentalis*	1	0	0	0	0	0	0	0	0	0	0	1	1	0	1	1	0	0	0	-
*Brachyhypopomus* sp. "palenque"	1	0	0	0	0	0	0	0	0	0	0	1	1	0	1	1	0	0	0	-
*Brachyhypopomus pinnicaudatus*	1	0	0	0	0	0	0	0	0	0	0	1	1	0	1	1	1	0	1	0
*Brachyhypopomus* sp. "provenzanoi"	1	0	0	0	0	0	0	0	0	0	0	1	1	0	1	0	-	0	0	-
*Brachyhypopomus* sp. "regani"	1	0	0	0	0	0	0	0	0	0	0	1	1	0	1	0	-	0	1	0
*Brachyhypopomus* sp. "sullivani"	1	0	0	0	0	0	0	0	0	0	0	1	1	0	1	0	-	0	1	0
*Brachyhypopomus* sp. "verdii"	1	0	0	0	0	1	-	-	1	0	0	1	1	0	1	1	1	0	1	0
*Brachyhypopomus walteri*	1	0	1	0	0	0	0	0	0	0	0	1	1	0	1	1	0	0	1	0
**Outgroups:**																				
*Gymnotus jonasi*	0	-	0	0	0	0	0	0	0	0	0	0	0	0	0	0	-	0	-	-
*Rhamphichthys marmoratus*	1	0	0	0	0	0	0	0	0	0	0	0	0	1	1	0	-	0	0	-
*Gymnorhamphichthys rondoni*	1	0	0	0	0	0	0	0	0	0	0	0	0	1	1	0	-	0	0	-
*Hypopomus artedi*	1	0	0	0	0	0	0	0	0	0	0	1	1	0	1	1	0	0	0	-
*Steatogenys duidae*	1	0	1	0	0	0	0	0	1	1	1	0	0	0	1	0	-	0	0	-
*Hypopygus lepturus*	1	0	1	1	0	1	-	-	0	1	1	1	-	0	1	0	-	0	0	-
*Microsternarchus bilineatus*	1	1	0	0	1	0	1	0	0	0	0	1	1	0	1	0	-	0	0	-
*Racenisia fimbriipinna*	1	1	1	1	1	1	-	-	0	0	0	1	1	0	1	0	-	0	0	-
*Sternopygus astrabes*	0	-	0	0	0	0	0	0	0	0	0	0	0	0	0	1	0	0	0	-
*** ***	**21**	**22**	**23**	**24**	**25**	**26**	**27**	**28**	**29**	**30**	**31**	**32**	**33**	**34**	**35**	**36**	**37**	**38**	**39**	**40**
**Ingroup:**																				
*Brachyhypopomus* sp. "alberti"	-	1	-	-	1	0	0	1	0	0	1	-	1	-	0	0	0	-	-	-
*Brachyhypopomus* sp. "arrayae"	-	0	0	0	1	1	0	1	0	0	1	-	1	-	0	0	0	-	-	-
*Brachyhypopomus* sp. "batesi"	0	1	-	-	1	1	0	1	0	0	0	1	1	-	0	0	0	-	-	-
*Brachyhypopomus beebei*	-	1	-	-	1	0	0	1	0	0	0	1	1	-	0	1	0	-	-	-
*Brachyhypopomus* sp. "belindae"	0	1	-	-	1	0	0	1	0	1	1	-	1	-	0	0	0	-	-	-
*Brachyhypopomus* sp. "benjamini"	1	1	-	-	1	1	0	0	1	0	1	-	1	-	0	0	0	-	-	-
*Brachyhypopomus bennetti*	-	0	1	0	1	0	0	1	0	0	0	1	1	-	1	1	0	-	-	-
*Brachyhypopomus bombilla*	-	0	1	0	1	1	0	1	0	0	0	1	1	-	0	0	0	-	-	-
*Brachyhypopomus brevirostris*	0	1	-	-	1	0	1	1	0	1	0	1	0	0	0	0	1	0	0	0
*Brachyhypopomus bullocki*	-	1	-	-	0	0	0	1	0	0	0	1	1	-	0	0	1	0	0	0
*Brachyhypopomus* sp. "cunia"	1	1	-	-	1	1	0	1	0	0	0	1	1	-	1	0	1	0	1	1
*Brachyhypopomus diazi*	0	0	1	0	1	0	0	1	0	0	0	1	0	1	0	0	0	-	-	-
*Brachyhypopomus draco*	0	0	0	0	0	0	0	1	0	0	0	1	1	-	0	0	0	-	-	-
*Brachyhypopomus sp*. *"flavipomus"*	0	1	-	-	1	1	0	1	0	0	1	-	1	-	0	0	0	-	-	-
*Brachyhypopomus gauderio*	-	0	1	0	1	1	0	1	0	0	1	-	1	-	0	1	0	-	-	-
*Brachyhypopomus* sp. "hamiltoni"	-	0	1	0	1	0	0	1	0	0	1	-	1	-	1	0	0	-	-	-
*Brachyhypopomus* sp. "hendersoni"	0	1	-	-	1	1	0	1	0	0	0	1	1	-	1	0	1	1	1	1
*Brachyhypopomus janeiroensis*	0	1	-	-	1	0	0	1	0	0	0	1	0	0	0	0	0	-	-	-
*Brachyhypopomus jureiae*	0	1	-	-	1	0	0	1	0	0	0	1	0	1	0	0	0	-	-	-
*Brachyhypopomus* sp. "menezesi"	-	1	-	-	0	1	0	1	0	0	0	1	1	-	0	0	0	-	-	-
*Brachyhypopomus occidentalis*	0	0	0	1	1	0	0	1	0	0	0	1	0	1	0	0	0	-	-	-
*Brachyhypopomus* sp. "palenque"	0	0	0	1	1	0	0	1	0	0	0	1	0	1	0	0	0	-	-	-
*Brachyhypopomus pinnicaudatus*	-	0	1	0	1	0	0	1	0	0	1	-	1	-	0	1	0	-	-	-
*Brachyhypopomus* sp. "provenzanoi"	0	1	-	-	1	0	0	0	0	0	1	-	1	-	0	0	0	-	-	-
*Brachyhypopomus* sp. "regani"	-	0	P	1	0	1	0	1	0	0	0	1	1	-	0	0	0	-	-	-
*Brachyhypopomus* sp. "sullivani"	-	0	0	0	1	0	0	1	0	0	0	1	0	0	0	0	0	-	-	-
*Brachyhypopomus* sp. "verdii"	-	1	-	-	1	0	0	1	0	0	1	-	0	0	0	0	0	-	-	-
*Brachyhypopomus walteri*	-	0	1	0	1	0	0	1	0	0	0	1	1	-	1	1	0	-	-	-
**Outgroups:**																				
*Gymnotus jonasi*	-	1	-	-	0	0	0	1	0	0	0	0	0	0	0	0	0	-	-	-
*Rhamphichthys marmoratus*	0	0	0	0	1	0	0	1	0	0	0	0	0	0	0	0	1	0	0	0
*Gymnorhamphichthys rondoni*	1	0	0	0	1	0	0	1	0	0	0	0	0	0	0	0	0	-	-	-
*Hypopomus artedi*	0	0	0	0	1	0	0	1	0	0	0	0	0	0	0	0	1	0	0	0
*Steatogenys duidae*	0	0	1	1	0	0	0	0	0	0	0	0	1	-	0	0	1	0	0	0
*Hypopygus lepturus*	1	1	-	-	0	0	0	1	0	0	0	1	0	0	0	0	0	-	-	-
*Microsternarchus bilineatus*	0	1	-	-	0	0	0	0	1	0	1	-	1	-	0	0	0	-	-	-
*Racenisia fimbriipinna*	0	0	1	0	0	1	0	0	1	0	1	-	1	-	0	0	0	-	-	-
*Sternopygus astrabes*	0	0	0	0	0	0	0	1	0	0	0	0	0	0	0	0	0	-	-	-
	**41**	**42**	**43**	**44**	**45**	**46**	**47**	**48**	**49**	**50**	**51**	**52**	**53**	**54**	**55**	**56**	**57**	**58**	**59**	**60**
**Ingroup:**																				
*Brachyhypopomus* sp. "alberti"	-	0	0	0	0	?	1	1	0	?	0	1	0	1	0	1	1	0	1	0
*Brachyhypopomus* sp. "arrayae"	-	0	0	0	0	?	1	1	0	?	0	1	0	1	0	1	1	0	1	0
*Brachyhypopomus* sp. "batesi"	-	0	0	0	0	?	1	0	0	?	0	1	0	0	0	1	1	0	0	0
*Brachyhypopomus beebei*	-	0	0	0	0	?	1	1	0	?	0	1	0	1	0	0	1	0	0	0
*Brachyhypopomus* sp. "belindae"	-	0	0	0	0	?	1	1	0	?	0	1	0	1	0	1	1	0	0	0
*Brachyhypopomus* sp. "benjamini"	-	0	0	0	1	?	1	0	0	?	0	1	0	0	0	1	1	0	0	0
*Brachyhypopomus bennetti*	-	1	0	0	0	0	1	0	0	?	0	1	0	1	0	1	1	0	0	0
*Brachyhypopomus bombilla*	-	0	0	0	0	?	0	2	0	?	0	1	0	0	0	1	1	0	0	1
*Brachyhypopomus brevirostris*	1	0	1	0	0	0	1	0	0	0	0	1	1	1	0	0	1	0	0	0
*Brachyhypopomus bullocki*	1	0	1	0	0	?	1	0	0	?	0	1	1	1	0	1	1	0	0	0
*Brachyhypopomus* sp. "cunia"	1	0	1	0	0	?	1	0	0	?	0	1	1	1	0	0	1	0	0	0
*Brachyhypopomus diazi*	-	0	0	0	0	0	0	0	0	0	1	1	0	0	0	0	1	0	0	0
*Brachyhypopomus draco*	-	0	0	0	0	?	1	1	0	?	0	1	0	1	0	0	1	0	0	0
*Brachyhypopomus sp*. *"flavipomus"*	-	0	0	0	0	?	1	1	0	?	0	1	0	0	0	0	1	1	0	0
*Brachyhypopomus gauderio*	-	0	0	0	0	?	1	1	0	?	0	1	0	1	0	0	1	0	0	0
*Brachyhypopomus* sp. "hamiltoni"	-	0	0	0	0	?	1	1	0	?	0	1	0	1	0	1	1	0	0	0
*Brachyhypopomus* sp. "hendersoni"	1	0	1	0	0	?	1	0	0	?	0	1	1	1	0	1	1	0	0	0
*Brachyhypopomus janeiroensis*	-	0	0	0	0	?	1	1	0	?	0	1	0	1	0	0	1	0	0	0
*Brachyhypopomus jureiae*	-	0	0	0	0	?	1	1	0	?	0	1	0	1	0	0	1	0	0	0
*Brachyhypopomus* sp. "menezesi"	-	0	0	0	0	?	0	2	0	?	1	1	0	0	0	1	1	0	0	1
*Brachyhypopomus occidentalis*	-	0	0	0	0	?	0	0	0	?	1	1	0	0	0	0	1	0	0	0
*Brachyhypopomus* sp. "palenque"	-	0	0	0	0	0	0	1	0	0	1	1	0	0	0	0	1	0	0	0
*Brachyhypopomus pinnicaudatus*	-	0	0	1	0	?	1	1	0	?	0	1	0	1	0	0	1	0	0	0
*Brachyhypopomus* sp. "provenzanoi"	-	0	0	0	1	?	1	0	0	?	0	1	0	0	0	1	1	0	0	0
*Brachyhypopomus* sp. "regani"	-	0	0	0	0	?	0	2	0	?	1	1	0	1	0	1	1	0	0	1
*Brachyhypopomus* sp. "sullivani"	-	0	0	0	0	?	0	2	0	?	1	1	0	0	0	1	1	0	0	0
*Brachyhypopomus* sp. "verdii"	-	0	0	0	0	?	1	1	0	?	0	1	0	0	1	0	1	0	0	0
*Brachyhypopomus walteri*	-	1	0	0	0	0	1	0	0	0	0	1	0	1	0	1	1	0	0	0
**Outgroups:**																				
*Gymnotus jonasi*	-	0	0	0	0	0	1	0	0	0	0	0	-	0	0	0	0	0	0	0
*Rhamphichthys marmoratus*	0	0	0	0	0	1	1	0	0	1	0	0	-	1	0	1	0	0	0	0
*Gymnorhamphichthys rondoni*	-	0	0	0	1	?	1	0	0	?	0	0	-	1	0	0	0	0	0	0
*Hypopomus artedi*	0	0	0	0	0	?	0	0	0	?	0	0	-	0	0	0	0	0	0	0
*Steatogenys duidae*	0	0	0	0	0	?	1	0	1	?	0	0	-	1	0	0	0	1	0	0
*Hypopygus lepturus*	-	0	0	0	0	1	1	-	1	1	?	0	-	1	0	0	0	1	0	0
*Microsternarchus bilineatus*	-	0	0	0	1	1	0	2	0	1	1	1	0	1	0	0	0	0	0	0
*Racenisia fimbriipinna*	-	0	0	0	1	1	0	-	0	1	1	1	0	0	0	0	0	-	0	0
*Sternopygus astrabes*	-	0	0	0	0	0	1	0	0	0	0	0	-	1	0	1	0	0	0	0

Ingroup species are ordered alphabetically. Outgroup taxa are ordered following the taxonomic scheme of Albert (2001). Symbols:— = character not applicable.? = unknown. P = polymorphic for 0 and 1.

We rooted our phylogenetic reconstructions with *Gymnotus*, based on Albert [20], which places the Gymnotidae as the sister taxon to all remaining gymnotiforms; although see alternative conclusions in Triques [32] and Alves-Gomes et al. [21]. We subjected the character matrix to a heuristic search in PAUP* [33] using the default options (stepwise addition, simple additional sequence, branch-swapping TBR, Max trees at 1000, and branches collapsed if maximum length is zero). We generated character diagnoses and synapomorphy lists, and performed tree manipulations using PAUP* and Mesquite 3.04 [34]. Ambiguous character distributions were resolved using accelerated transformation (ACCTRAN), which maximizes reversals over parallelism [35].

### Molecular-based and total evidence phylogenetic reconstruction

Our molecular analyses included 26 ingroup species (all 28 *Brachyhypopomus* species used in our morphological phylogenetic reconstruction except *B*. sp. “arrayae” and *B*. sp. “menezesi”, for which tissue samples were unavailable; Table 1), and nine additional outgroups—also matching those from our morphological phylogenetic reconstruction (Table 2). A complete list of sequenced specimens included in our molecular analyses is provided in Table 3. A minimum of two individuals for each ingroup and outgroup species were included. For geographically widespread species of *Brachyhypopomus*, representatives of populations from major drainages were included where possible (see drainage denotations in parentheses after species names in Table 3).

#### DNA isolation, PCR, and sequencing

For each sample, we isolated total genomic DNA from muscle tissue using DNeasy Tissue Kits (QIAGEN) according to the manufacturer’s protocols. The polymerase chain reaction (PCR) was used to amplify approximately 1100 base pairs of the mitochondrial cytb gene, and approximately 1000 base pairs of the nuclear recombination activating gene-2 (rag2). We amplified using primers in the adjacent glutamine (cytbF 5’-TGACTTGAAGAACCACCGTTG-3’) and threonine (cytbR 5’-CTCCGATCTTCGGATTACAAG-3’) transfer RNAs, with PCR carried out in 25μl or 50μl volumes including 10x PCR buffer (50mM KCL, 20mM Tris-HCL, pH 8.4), 200 μM of each dNTP, 3mM MgCl_2_, 0.4 μM of each primer, 0.5 to 1 unit of Taq DNA Polymerase (Invitrogen) and 0.5 to 1 μl of DNA extract. Thermal cycling conditions were: an initial hold step of 95°C for 30s, followed by 35 cycles of 30s denaturation at 95°C, 60s annealing at 50° to 58°C, and 90s extension at 72°C. A final hold step of 300s at 72°C was added after the last cycle.

The amplification of rag2 for the majority of taxa was accomplished using the primers RAG2JF1 (5’-TGCTATCTTCCACCACTGCGVTGCC-3’) and RAG2JR1 (5’-TCATCYTCCTCATCKTCCTCATTGTA-3’) designed for this study. For some taxa, additional new amplification primers were designed and used (sequences available on request from NRL). PCR reaction volumes and concentrations followed those outlined for cytb, except that 2–5μl of DNA was used in each reaction. Thermal cycling conditions for rag2 used a touchdown protocol of one cycle of initial denaturation at 95°C for 30s, followed by denaturation, 58°C, 56°C, 54°C, 52°C, for two cycles each, then 50°C for 32 cycles annealing, followed by extension at 72°C for 90s. PCR products were purified using QIAGEN PCR purification kits (QIAGEN), and sequenced at the Centre for Applied Genomics facility at SickKids Hospital, Toronto.

#### Phylogenetic analysis

We edited and aligned sequences using Geneious Pro v5.5.6 [36]. For both rag2 and cytb, alignment was trivial and no insertions/deletions were detected. DNA sequences were concatenated by individual and combined with the morphological dataset to produce a total evidence matrix of 2131 characters for 105 operational taxonomic units. We analyzed these data using both Bayesian and parsimony approaches. Bayesian analyses allow the use of more complex models of molecular evolution and assessment of node support across a distribution of most-probable trees.

Using MrBayes v3.2.2 [37, 38], we conducted a Bayesian Inference (BI) phylogenetic analysis of (1) each gene separately, (2) both genes combined, and (3) both genes combined with morphological data (total evidence). For these analyses, we employed the best-fit substitution models for each partition selected by PartitionFinder v.1.1.1 [39], with the GTR+G model for Rag2, the GTR+I+G model for cytb, and the Mkv model for morphology. For each analysis, we conducted two independent MrBayes runs, each with four chains for 20 million generations, sampling every 1000 generations to ensure standard deviation of split frequencies were below 0.01 and potential scale reduction factors were close to 1.0. For combined analyses, partitions were unlinked. MrBayes runs were inspected in Tracer v1.6 [40] to ensure convergence of parameter estimates, and we confirmed that the Effective Sample Size (ESS) values of all parameters were well above 200. The first 25% of each run was discarded as burn-in, and we combined the remaining trees and parameter estimates to determine posterior distributions. Trees and posterior probabilities for nodes were inspected using FigTree 1.4.0 [41].

We conducted parsimony analysis of the total evidence dataset using PAUP* [33]. We defined three data partitions: rag2, cytb, and morphology, and used the parsimony-based incongruence length difference test (ILD or partition homogeneity test of PAUP*) to explore the phylogenetic congruence of these partitions [33, 42]. Pairwise ILD comparison between rag2 and cytb partitions indicated congruence (p > 0.05), but pairwise comparisons of morphology with each molecular partition (cytb and rag2) indicated incongruence (p < 0.05). Combining incongruent data partitions can increase the accuracy of phylogenetic reconstruction [43, 44]. Therefore, we proceeded with total evidence analysis (all partitions combined), but also assessed the phylogenetic hypotheses based on individual partitions.

For all analyses, we used the heuristic search algorithm with 1000 replicates of random addition of taxa, and TBR branch swapping. All trees were rooted using *Gymnotus*. Bootstrap values [45] were calculated in PAUP* using the heuristic search option (1000 replicates, 10 random taxon additions), and decay indices (Bremer support values) [46]–were calculated using the program TreeRot.v3 [47].

We used maximum likelihood (ML) to reconstruct ancestral character states for three ecological characters and geographic distributions (see below). We reconstructed characters on the BI total evidence tree using Mesquite v. 3.04 [34] using the Mk model of Pagel [48], with branch lengths set to 1.

### Illustrations & photography

Camera lucida tracings of micro-dissected osteological structures were made with a drawing tube attached to a Meiji Techno RZ stereomicroscope. These tracings were used in combination with digital photographs of the equivalent structure (taken through the stereoscope) to add stipple-texture in the final osteological illustrations. All digital rendering was conducted in Adobe Illustrator 2014.1.1 (Adobe Corporation, San Jose, CA). Some illustrations (where noted in figure captions) were prepared from the right side of dissected specimens and are inverted left-to-right to present anterior features in the traditional left position. Photographs of cleared and stained specimens were taken with a video camera or Nikon Coolpix P5100 digital camera attached to the Meiji microscope, or on an illuminated light table with a Nikon Coolpix 5100 camera attached to a Nikon EZ-Micro field microscope at x20 magnification.

## Results

### Morphology-based phylogenetic reconstruction

**Fig 1:**
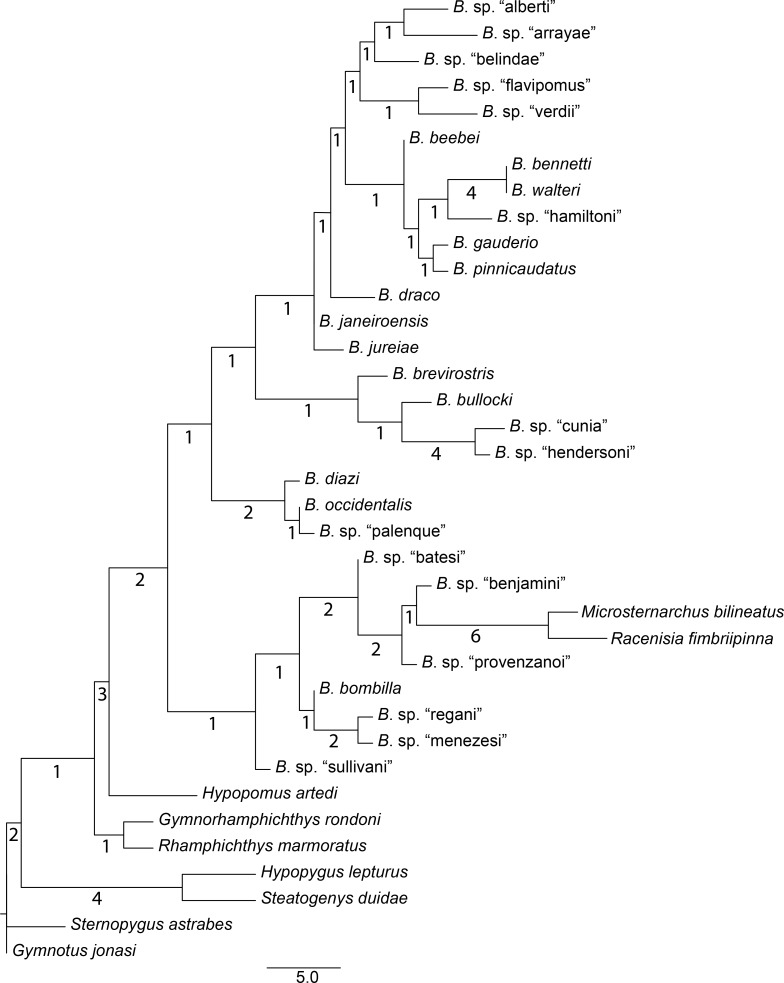
Parsimony phylogeny for *Brachyhypopomus* based on morphological data. Tree is the strict consensus of 127 equally parsimonious trees (60 characters, CI = 0.42, RC = 0.28, RI = 0.68). Numbers below branches denote decay indices. Branch lengths are proportional to reconstructed character state changes. Terminal taxa are 28 species of *Brachyhypopomus* and 9 outgroup taxa.

Our parsimony search yielded 127 shortest trees, each with a length of 151 steps (consistency index [CI] = 0.42, rescaled consistency index [RC] = 0.28, retention index [RI] = 0.68). The strict consensus tree is presented in Fig 1. The decay index (Bremer support) values across the tree are generally low, with the exception of some highly-supported terminal clades such as *B*. *bennetti* + *B*. *walteri*, *B*. sp. “cunia” + *B*. sp. “hendersoni”, and the Microsternarchini. *Brachyhypopomus* is not monophyletic in this analysis, because *Microsternarchus* and *Racenisia* are nested with *Brachyhypopomus*.

### Molecular-based phylogenetic reconstruction

**Fig 2:**
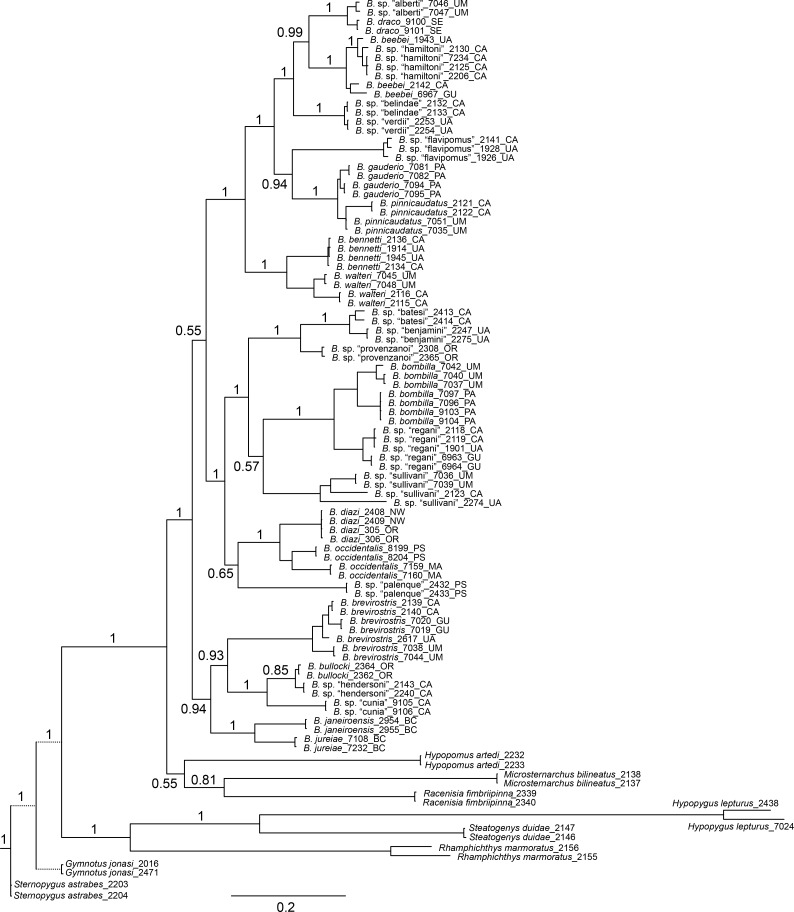
Bayesian inference phylogeny for *Brachyhypopomus* based on cytb. Bayesian posterior probabilities shown by nodes (not shown for intraspecific relationships). Branch lengths proportional to substitutions per site. Terminal taxa are 83 individuals representing 26 species of *Brachyhypopomus* and 16 individuals representing 8 outgroup taxa. Branch lengths for *Gymnotus* and *Sternopygus* (represented with dashed lines) are reduced. See Table 3 for list of sequenced specimens. Terminal two letter codes refer to the drainage units described in ‘Geographic and ecological distributions’ (Materials and Methods).

**Fig 3:**
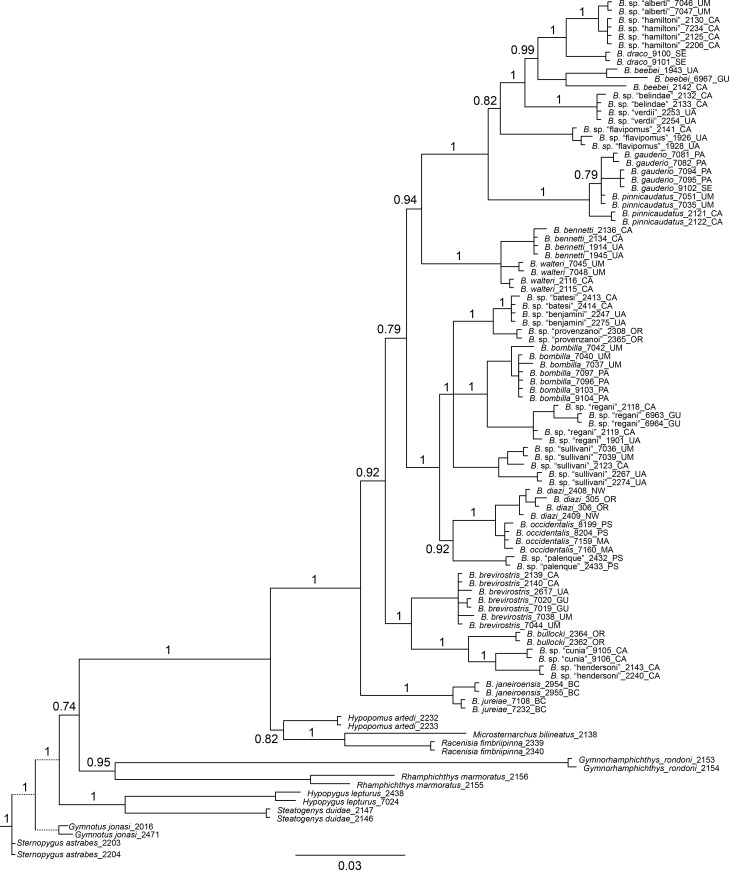
Bayesian inference phylogeny for *Brachyhypopomus* based on rag2. Bayesian posterior probabilities shown by nodes (not shown for intraspecific relationships). Branch lengths proportional to substitutions per site. Terminal taxa are 85 individuals representing 26 species of *Brachyhypopomus* and 17 individuals representing 9 outgroup taxa. Branch lengths for *Gymnotus* and *Sternopygus* (represented with dashed lines) are reduced. See Table 3 for list of sequenced specimens. Terminal two letter codes refer to the drainage units described in ‘Geographic and ecological distributions’ (Materials and Methods).

**Fig 4:**
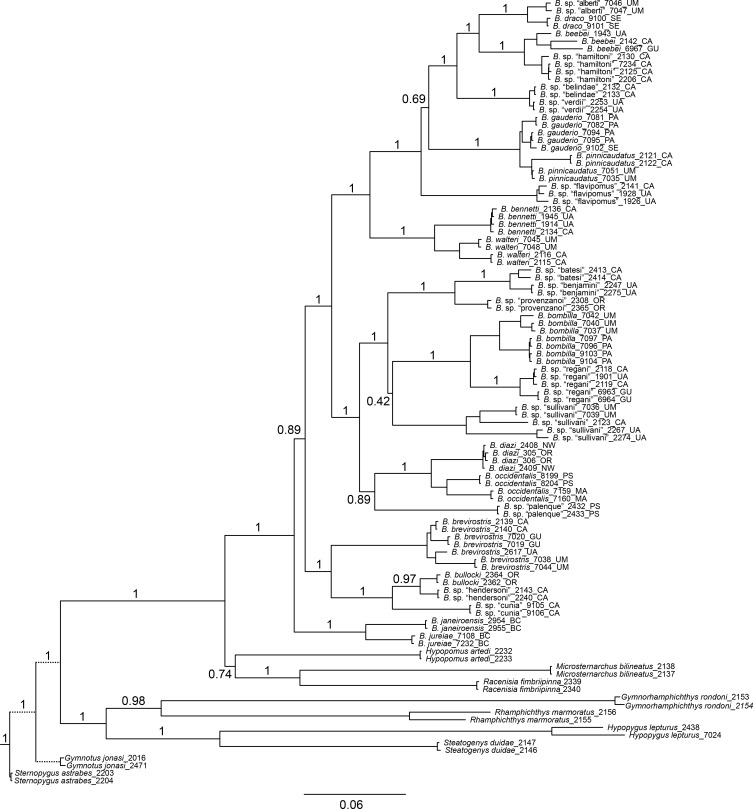
Bayesian inference phylogeny for *Brachyhypopomus* based on cytb and rag2. Bayesian posterior probabilities shown by nodes (not shown for intraspecific relationships). Branch lengths proportional to substitutions per site. Terminal taxa are 85 individuals representing 26 species of *Brachyhypopomus* and 18 individuals representing 9 outgroup taxa. Branch lengths for *Gymnotus* and *Sternopygus* (represented with dashed lines) are reduced. See Table 3 for list of sequenced specimens. Terminal two letter codes refer to the drainage units described in ‘Geographic and ecological distributions’ (Materials and Methods).

Figs 2, 3 and 4 show BI phylogenies for *Brachyhypopomus* based on cytb, rag2, and cytb+rag2 combined, respectively. For all trees, terminal two-letter code in labels of ingroup specimens indicate drainage unit (see ‘Geographic and ecological distributions‘ in Materials and Methods). These topologies are highly concordant, showing that both genes provide useful phylogenetic information. As expected, branch lengths are generally longer for the mitochondrial cytb gene compared to the nuclear rag2 gene; mitochondrial protein-coding genes generally evolve more quickly than nuclear protein-coding genes. Cytb typically provides greater resolution of relationships within species and between closely related species.

### Total evidence phylogenetic reconstruction

**Fig 5:**
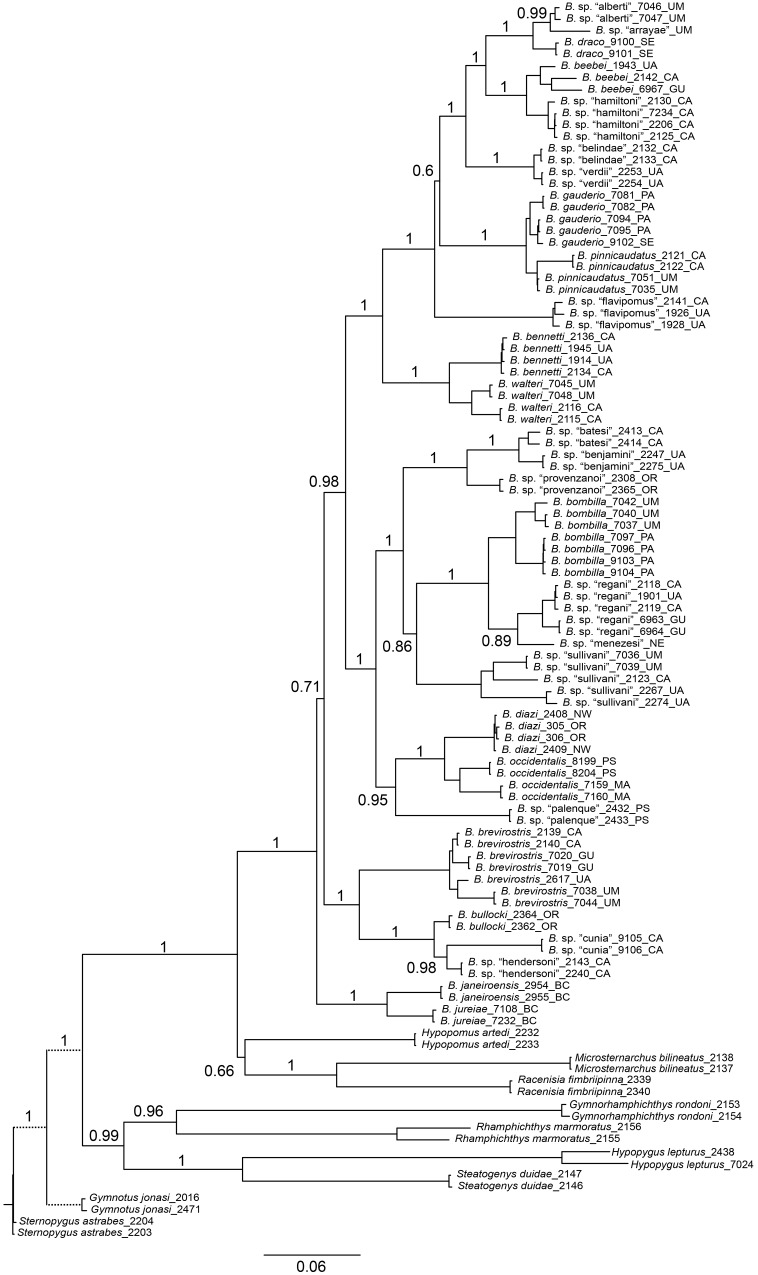
Bayesian inference phylogeny for *Brachyhypopomus* based on total evidence (cytb, rag2, and morphology). Bayesian posterior probabilities shown by nodes (not shown for intraspecific relationships). Branch lengths proportional to substitutions per site. Terminal taxa are 87 individuals representing 28 species of *Brachyhypopomus* and 18 individuals representing 9 outgroup taxa. Branch lengths show substitutions per site. Branch lengths for *Gymnotus* and *Sternopygus* (represented with dashed lines) are reduced. See Table 3 for list of sequenced specimens. Terminal two letter codes refer to the drainage units described in ‘Geographic and ecological distributions’ (Materials and Methods).

**Fig 6:**
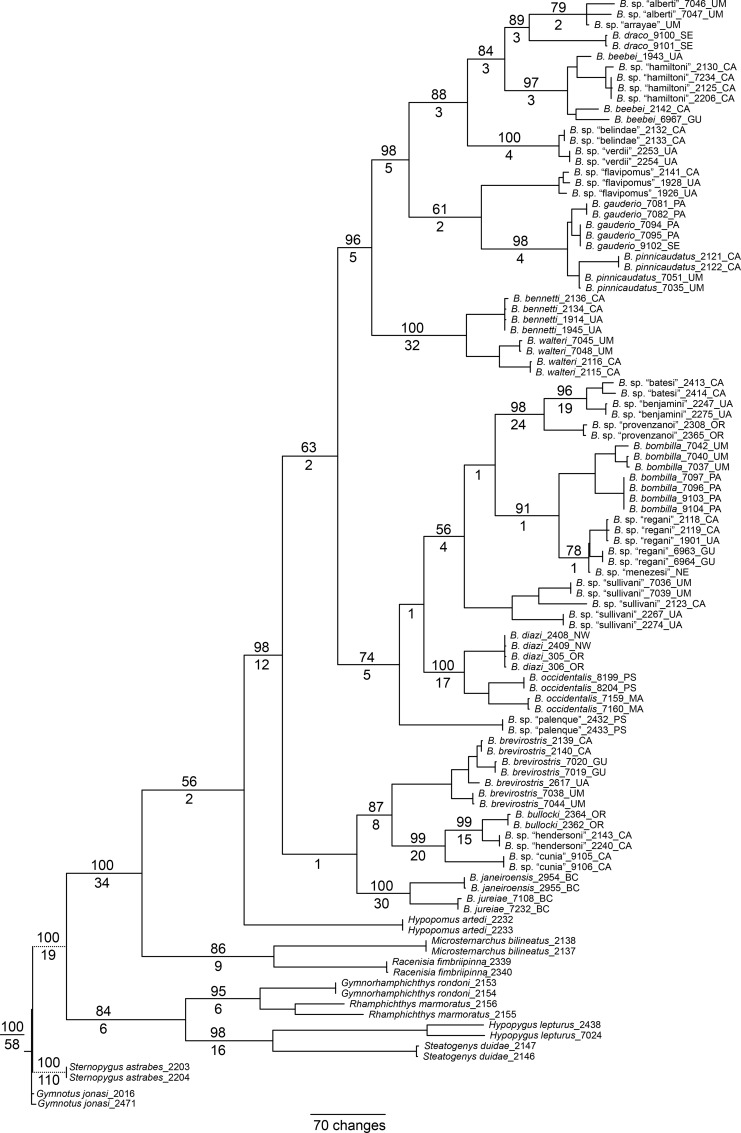
Parsimony phylogeny for *Brachyhypopomus* based on total evidence (cytb, rag2, and morphology). Tree shows strict consensus of 3244 equally parsimonious trees each with a length of 4243 steps (CI = 0.36, RCI = 0.29, RI = 0.81). Numbers above branches denote bootstrap proportions; numbers below branches denote decay indices; support values not shown for intraspecific relationships. Branch lengths proportional to reconstructed character state changes. Terminal taxa are 87 individuals representing 28 species of *Brachyhypopomus* and 18 individuals representing 9 outgroup taxa. Branch lengths for *Gymnotus* and *Sternopygus* (represented with dashed lines) are reduced. See Table 3 for list of sequenced specimens. Terminal two letter codes refer to the drainage units described in ‘Geographic and ecological distributions’ (Materials and Methods).

Our analyses of the total evidence matrix comprising the combined data from morphology, cytb, and rag2 yielded the trees shown in Fig 5 (Bayesian inference) and Fig 6 (parsimony). These trees include two to seven individuals for each sequenced species, and one individual each of *B*. sp. “arrayae” and *B*. sp. “menezesi”–for which only morphological data are available.

**Fig 7:**
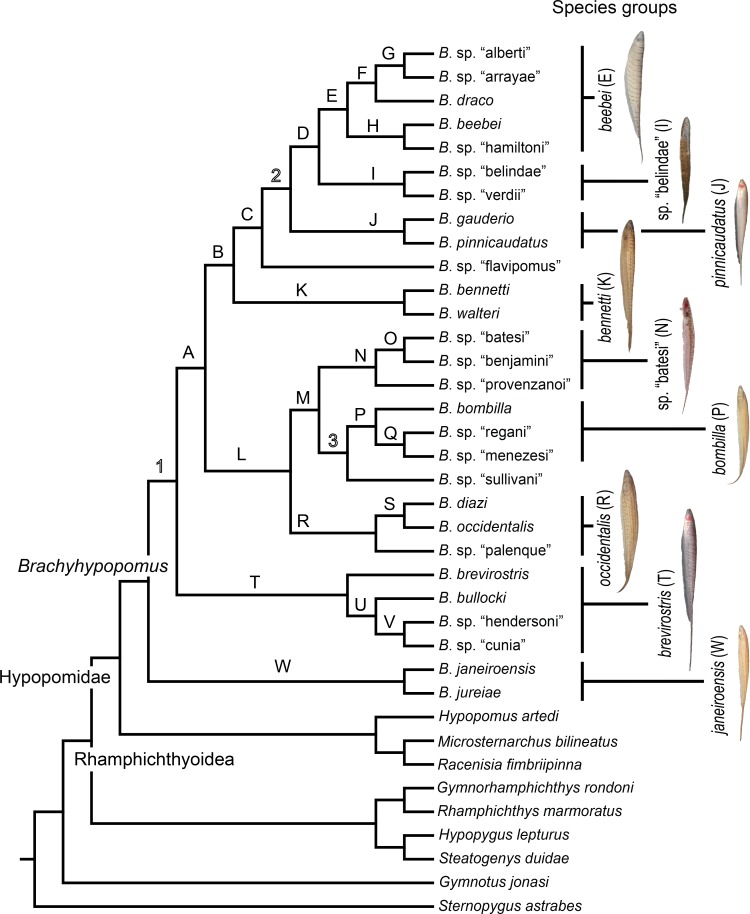
Species phylogeny for *Brachyhypopomus* based on Bayesian Inference of cytb, rag2, and morphology, with duplicate individuals for each species removed. Letters denote well-supported clades and numbers denote poorly-supported clades (see text for details). The label Hypopomidae follows Maldonado-Ocampo et al. (2014). Inset photographs are: *B*. *beebei*, *B*. sp. “belindae”, *B*. *pinnicaudatus*, *B*. *bennetti*, *B*. sp. “batesi”, *B*. *bombilla*, *B*. *occidentalis*, *B*. *brevirostris*, and *B*. *janeiroensis*. Photographs are uniformly scaled; scale bar = 10 mm.

In Fig 7 we provide a simplified cladogram based on the Bayesian total evidence phylogram depicted in Fig 5, and use this as our preferred topology for discussing morphological character state transitions, ecological characters, and phylogenetic patterns of geographical distributions in *Brachyhypopomus* (see below). We compared optimizations on the total evidence parsimony tree (not shown), and our inferences are robust to different tree-building methods. Terminal branches in Fig 7 represent species (28 *Brachyhypopomus* and 9 outgroup species) and we use an alphabetical scheme (A-W) to label clades that are well-supported–defined arbitrarily as node support exceeding 0.88 Bayesian Posterior Probability (PP). Clades that are labeled numerically (1–3) are those for which we have less confidence (PP < 0.88), and which are more likely to change in future analyses. We recognize the limitations of allocating a cut-off between well-supported and poorly-supported nodes, but make the distinction as a heuristic device to simplify our discussions. In Fig 7 we also label some of the well-supported clades as species groups. These are used to facilitate our discussions, not to imply equal taxonomic rank.

### Morphological character descriptions and analyses

Here we discuss the 60 morphological characters used for phylogenetic analysis, ordered by discrete body systems in an approximately anterior to posterior sequence. For each character we provide a summary description of the character states and a discussion of the distribution of character states among the ingroup and outgroup taxa. For each character we list the consistency and retention indices for each character based on the topology for the Bayesian total evidence analysis (summarized in Fig 7). A detailed synapomorphy scheme for morphological characters in *Brachyhypopomus*, also based on Bayesian total evidence topology, is provided in S3 Appendix. The synapomorphy scheme indicates character state changes at each node, and indicates whether they are ambiguous or unambiguous, and homoplastic or non-homoplastic.

#### Antorbital, supraorbital, infraorbitals, and associated structures

1. *Occurrence of an undescribed bone above maxilla*. (0) absent; (1) present (CI = 1.00; RI = 1.00).

**Fig 8:**
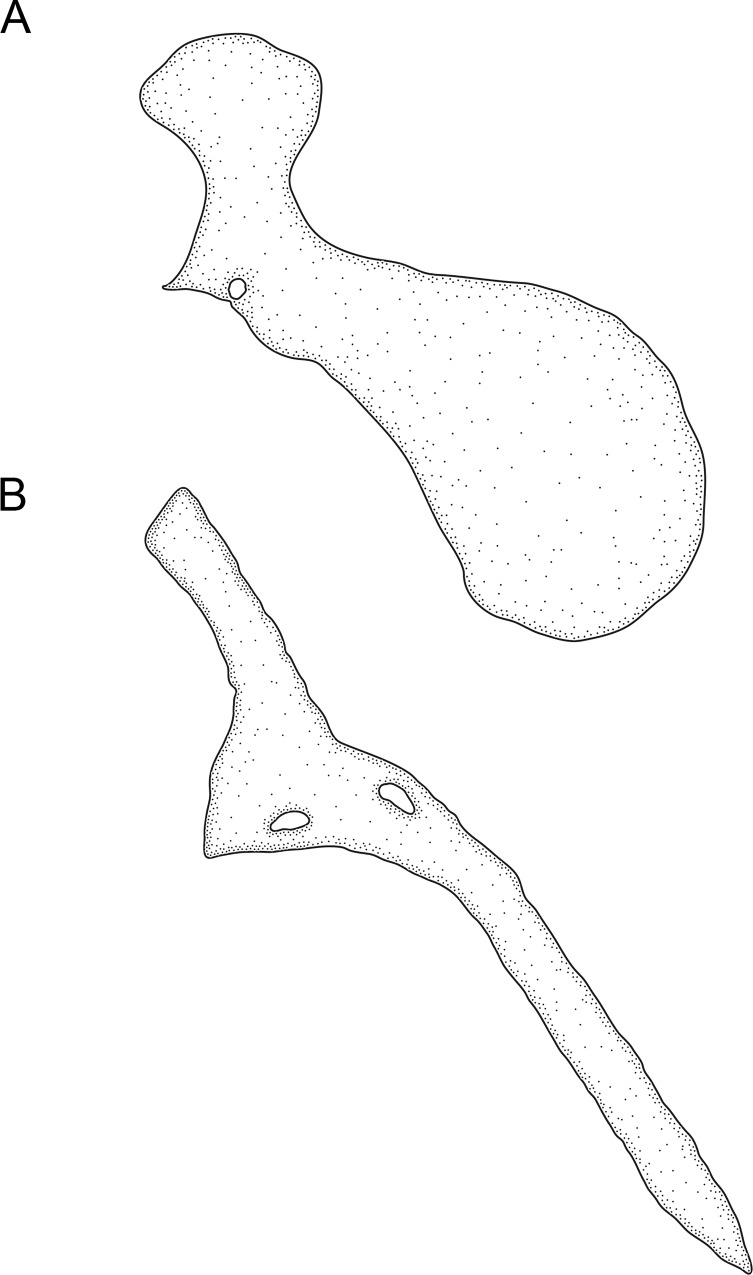
Undescribed bone located above the maxilla. In: (A) *Brachyhypopomus beebei*, MCP 45450 (WC06.090600), female, 152 mm. (B) *Racenisia fimbriipinna*, UF 177352, immature, 86 mm TL. Left side, lateral view. Anterior to left.

An as-yet undescribed bone in the Rhamphichthyoidea, located above the maxilla, was documented by de Santana and Crampton [49] as part of a discussion of the homology of the infraorbital series, antorbital, and associated structures in *Hypopygus*. This bone has elsewhere been incorrectly identified as the antorbital [18, 20, 22, 50]) or first infraorbital [19, 32]. It is absent (state 0; Mago-Leccia [51]: 57, fig 8) in *Gymnotus* and *Sternopygus*. Conversely, this bone is present (state 1; Fig 8A and 8B; Chardon & de la Hoz [52]: 18, fig 2; Triques [32]: 98, fig 5; Mago-Leccia [19]: 175, fig 77; de Santana & Crampton [49]: figs. 2–4) in all Rhamphichthyoidea (i.e. all *Brachyhypopomus* species, and in *Gymnorhamphichthys*, *Hypopomus*, *Hypopygus*, *Microsternarchus*, *Racenisia*, *Rhamphichthys*, and *Steatogenys*).

2. *Form of undescribed bone above maxilla*. (0) wide; (1) narrow (CI = 1.00; RI = 1.00).

The undescribed bone above the maxilla is distinctly wide and laterally expanded, width 40–60% of length (state 0; Fig 8A; Chardon & De La Hoz [52]: 18, fig 2; Triques [32]: 98, fig 5; de Santana & Crampton [49]: 1105, fig 3) in all species of *Brachyhypopomus*, and in *Gymnorhamphichthys*, *Hypopomus*, *Hypopygus*, *Rhamphichthys*, and *Steatogenys*. Conversely, this bone is narrow, width 10–20% of length (state 1; Fig 8B; Mago-Leccia [19]: 175, fig 77) in *Microsternarchus* and *Racenisia*. This character is not applicable to *Gymnotus* and *Sternopygus*, in which the undescribed bone above the maxilla is absent (see Character 1).

3. *Occurrence of antorbital*. (0) present; (1) absent (CI = 0.33; RI = 0.50).

**Fig 9:**
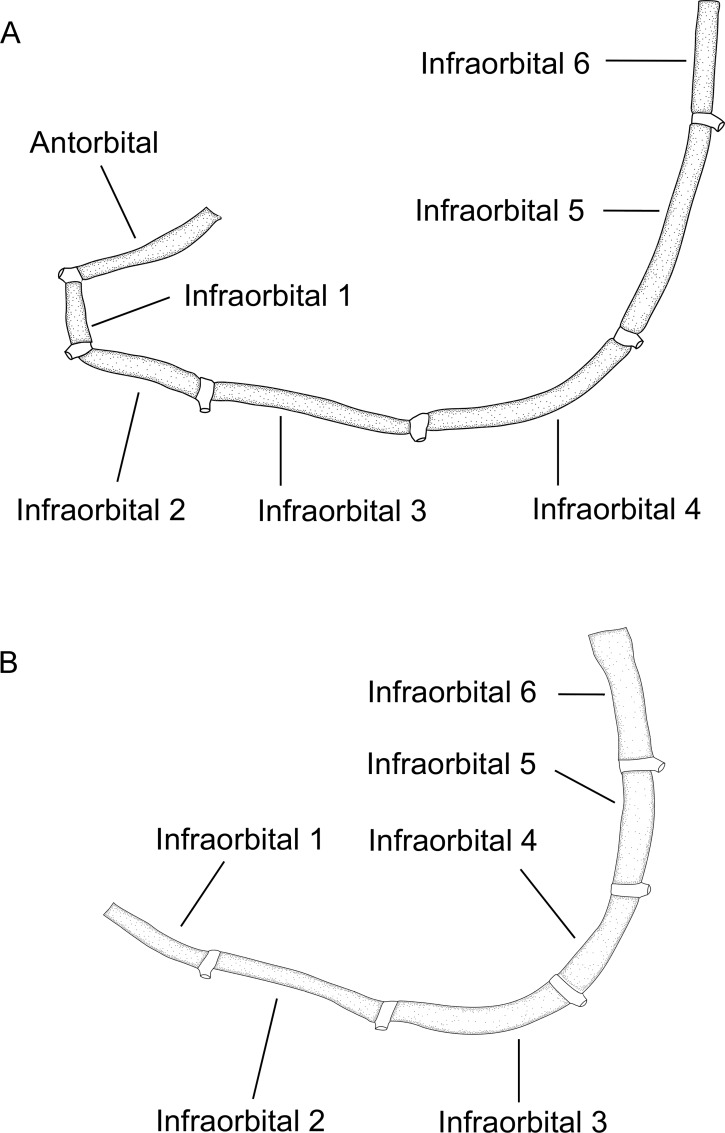
Antorbital and infraorbital canal series. In: (A). *Brachyhypopomus brevirostris*. MCP 44605 (WC06.010596), male, 374 mm TL. Left side, lateral view. Anterior to left. (B). *Brachyhypopomus bennetti*. MCP 45346, 1 (WC04.290696), female, 156 mm TL. Left side, lateral view. Anterior to left.

In gymnotiforms the antorbital is located ventral to the cavity of the posterior naris and is not to be confused with the undescribed bone that we discuss in Characters 1 and 2. The antorbital is present (state 0, Fig 9A; Albert et al. [53]: 385, 386, figs. 4,6) in all species of *Brachyhypopomus* except *B*. *bennetti* and *B*. *walteri*, and is also present (state 0) in *Gymnorhamphichthys*, *Gymnotus*, *Hypopomus*, *Microsternarchus*, *Rhamphichthys*, and *Sternopygus*. The antorbital is absent (state 1; Fig 9B) in *B*. *bennetti* and *B*. *walteri*, and also *Hypopygus*, *Racenisia*, and *Steatogenys*. The antorbital of *Brachyhypopomus* (where present) and *Gymnorhamphichthys*, *Gymnotus*, *Hypopomus*, *Microsternarchus*, *Rhamphichthys* comprises a vertically oriented ossified or cartilaginous tube–either a straight vertical tube or a slightly S-shaped tube (diagonally oriented in a dorso-posterior direction in *Brachyhypopomus*). The antorbital of *Sternopygus* forms an enlarged partial cylinder (Fink & Fink [54]: 316, fig 7; Lundberg & Mago-Leccia [55]: 56, fig 2).

4. *Occurrence of infraorbital series*. (0) present; (1) absent (CI = 0.50; RI = 0.00).

The infraorbital series is present (state 0; Triques [32]: 102, fig 9) in all species of *Brachyhypopomus*, and in *Gymnorhamphichthys Gymnotus*, *Hypopomus*, *Microsternarchus*, *Rhamphichthys*, *Steatogenys*, and *Sternopygus*. The infraorbital series is absent (state 1; Triques, [32]: 98, fig 5; Mago-Leccia, [19]: 175, fig 77) in *Hypopygus* and *Racenisia*.

5. *Association of infraorbital canal aperture and sphenotic spine*. (0) infraorbital canal opening located at vertical to and slightly anterior to sphenotic spine; (1) infraorbital canal opening distinctly anterior to sphenotic spine (CI = 1.00; RI = 1.00).

**Fig 10:**
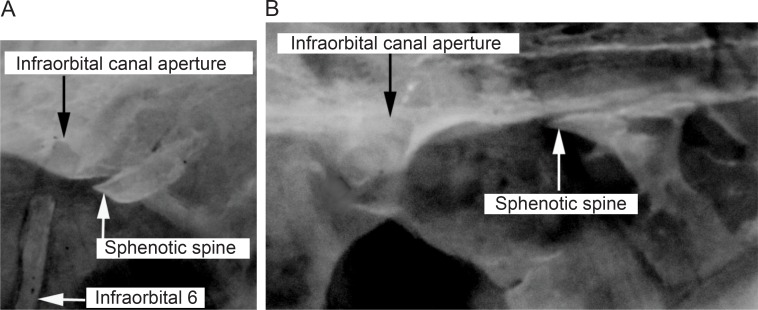
Macro-photograph of the neurocranium showing the relation between the infraorbital canal aperture and sphenotic spine. In: (A) *Brachyhypopomus brevirostris*, MCP 44605 (WC06.010596), male, 374 mm TL. (B) *Racenisia fimbriipinna*, UF 177352, immature, 86 mm TL. Left side, lateral view. Anterior to left.

The infraorbital canal opening is located at a vertical to and slightly anterior to the sphenotic spine (state 0; Fig 10A) in all species of *Brachyhypopomus*, and in *Gymnorhamphichthys*, *Gymnotus*, *Hypopomus*, *Hypopygus*, *Rhamphichthys*, *Steatogenys*, and *Sternopygus*. Conversely, the infraorbital canal opening is distinctly anterior to the sphenotic spine (state 1; Fig 10B) in *Microsternarchus* and *Racenisia*. In taxa exhibiting character state 1, the distance between the infraorbital canal opening and sphenotic spine is contained approximately one to one and a half times in the length of the sphenotic spine (Fig 10B).

6. *Occurrence of parietal branch of the supraorbital canal*. (0) present; (1) absent (CI = 0.20; RI = 0.33).

The parietal branch of the supraorbital canal, which runs over the frontal towards the parietal, is present (state 0; [49]: 1104, fig 2) in the adults of all *Brachyhypopomus* species except *B*. sp. “alberti”, *B*. sp. “arrayae”, *B*. sp. “belindae”, *B*. sp. “hamiltoni”, and *B*. sp. “verdii”, and is also present (state 0) in most adult specimens of *Gymnorhamphichthys*, *Gymnotus*, *Hypopomus*, *Microsternarchus*, *Rhamphichthys*, *Steatogenys*, and *Sternopygus*. The parietal branch of the supraorbital canal is absent (state 1; Triques [32]: 98, fig 5; Mago-Leccia [19]: 175, fig 77; [49]: 1105, fig 3) in *Brachyhypopomus* sp. “alberti”, *B*. sp. “arrayae”, *B*. sp. “belindae”, *B*. sp. “hamiltoni”, and *B*. sp. “verdii”, and also in *Hypopygus* and *Racenisia*. We noted that in small specimens of some *Brachyhypopomus* species the parietal branch of the supraorbital canal over the frontal is absent (e.g. *B*. *brevirostris*, MCP 46935, 1, post-larval, 30 mm).

7. *Position of parietal branch of the supraorbital canal*. (0) branch positioned at vertical above infraorbital canal aperture; (1) branch located distinctly posterior to infraorbital canal aperture (CI = 1.00; RI = 0.00).

The parietal branch of the supraorbital canal over the frontal is positioned at a vertical above the infraorbital canal aperture (following the nomenclature of Arratia & Huaquin [31]) (state 0) in all *Brachyhypopomus* species which possess the parietal branch of the supraorbital canal, and in *Gymnorhamphichthys*, *Gymnotus*, *Hypopomus*, *Rhamphichthys*, *Steatogenys*, and *Sternopygus*. Conversely, the parietal branch of the supraorbital canal is located distinct posterior to the infraorbital canal aperture (state 1) in *Microsternarchus*. This character is not applicable to *B*. sp. “alberti”, *B*. sp. “arrayae”, *B*. sp. “belindae”, *B*. sp. “hamiltoni”, *B*. sp. “verdii”, and also *Hypopygus* and *Racenisia*, which lack the parietal branch of the supraorbital canal in adults (see Character 6).

8. *Association of parietal branch of supraorbital canal with frontal*. (0) included in frontal; (1) independent from frontal (CI = 1.00; RI = 0.00).

The parietal branch of the supraorbital canal is included in (fused to) the frontal (state 0) in all species of *Brachyhypopomus* which possess a parietal branch of the supraorbital canal (except *B*. sp. “flavipomus”) and in *Gymnorhamphichthys*, *Gymnotus*, *Hypopomus*, *Microsternarchus*, *Rhamphichthys*, *Steatogenys*, and *Sternopygus*. The parietal branch of the supraorbital canal consists of an independent tube over the frontal (state 1) in *Brachyhypopomus* sp. “flavipomus”. This character is not applicable to *B*. sp. “alberti”, *B*. sp. “arrayae”, *B*. sp. “belindae”, *B*. sp. “hamiltoni”, *B*. sp. “verdii”, and also *Hypopygus* and *Racenisia*, which lack the parietal branch of the supraorbital canal in adults (see Character 6).

9. *Association of supraorbital canal and frontal*. (0) supraorbital canal included in frontal; (1) supraorbital canal independent from frontal (CI = 0.33; RI = 0.00).

The supraorbital canal (not including its parietal branch) is included in (fused to) the frontal (state 0) in all species of *Brachyhypopomus*, except *B*. sp. “flavipomus” and *B*. sp. “verdii”, and is also included in the frontal (state 0) in all outgroups except *Steatogenys*. The supraorbital canal is independent from the frontal (state 1) in *B*. sp. “flavipomus” and *B*. sp. “verdii”, and in *Steatogenys*.

#### Neurocranium

10. *Extent of lateral margin of frontal*. (0) broad, lateral margin of frontal extends over dorsal margin of orbitosphenoid; (1) narrow, lateral margin of frontal does not extend over dorsal margin of orbitosphenoid (CI = 1.00; RI = 1.00).

The lateral margin of the frontal is broad, extending over the dorsal margin of the orbitosphenoid (state 0) in all species of *Brachyhypopomus*, and in all outgroups except *Hypopygus* and *Steatogenys*. The lateral margin of the frontal is narrow, not extending over the dorsal margin of orbitosphenoid (state 1) in *Hypopygus* and *Steatogenys*.

11. *Extent of lateral margin of parietal*. (0) broad, lateral margin of parietal extends over dorsal margin of postotic canal; (1) narrow, lateral margin of parietal does not extend over dorsal margin of postotic canal (CI = 1.00; RI = 1.00).

The lateral margin of the parietal is broad, extending over the dorsal margin of the postotic canal (state 0) in all species of *Brachyhypopomus*, and in all outgroups except *Hypopygus* and *Steatogenys*. The lateral margin of the parietal is narrow, not extending over the dorsal margin of the postotic canal (state 1) in *Hypopygus* and *Steatogenys* (Steatogeni).

12. *Occurrence of vomer*. (0) present; (1) absent (CI = 0.50; RI = 0.75).

The vomer is present, its anterior portion contacting the posterior portion of the mesethmoid (state 0; de la Hoz & Chardon [56]: 127, fig 4a,b) in *Gymnorhamphichthys*, *Gymnotus*, *Rhamphichthys*, *Steatogenys*, and *Sternopygus*. The vomer is absent (state 1; de la Hoz & Chardon [56]: 127, fig 4c,e) in all species of *Brachyhypopomus*, and in *Hypopomus*, *Hypopygus*, *Microsternarchus*, and *Steatogenys*.

13. *Relative size of ventral ethmoid*. (0) occupying approximately three-fourth of the nasal septum; (1) occupying approximately one-fourth of the nasal septum (CI = 1.00; RI = 1.00).

The ventral ethmoid is large, occupying approximately three-fourths of the nasal septum in *Gymnorhamphichthys*, *Gymnotus*, *Rhamphichthys*, *Steatogenys*, and *Sternopygus* (state 0). The ventral ethmoid occupies about one-fourth of the nasal septum in all species of *Brachyhypopomus*, and in *Hypopomus*, *Microsternarchus*, and *Racenisia* (state 1). This character is not applicable to *Hypopygus*, in which the ventral ethmoid is absent in adults.

14. *Occurrence of posttemporal foramen*. (0) absent; (1) present (CI = 1.00; RI = 1.00).

The posttemporal foramen is absent (state 0) in all species of *Brachyhypopomus*, and in all outgroups except *Gymnorhamphichthys* and *Rhamphichthys*. The posttemporal foramen is present (state 1) in *Gymnorhamphichthys* and *Rhamphichthys*.

15. *Composition of Baudelot’s ligament*. (0) non-ossified; (1) ossified (CI = 1.00; RI = 1.00).

Baudelot’s ligament is present as a non-ossified structure (state 0) in *Gymnotus* and *Sternopygus* (state 0). Conversely it occurs as an ossified form (state 1) in all Rhamphichthyoidea (all species of *Brachyhypopomus*, and *Gymnorhamphichthys*, *Hypopomus*, *Hypopygus*, *Microsternarchus*, *Racenisia*, *Rhamphichthys*, and *Steatogenys*). In addition to observing ossification of Baudelot's ligament in adult specimens of the above taxa, we also observed it in small individuals of the following taxa, indicating that ossification occurs early in growth: *Brachyhypopomus* (*B*. *diazi*, UF 174333, 1, 20.0 mm); *Hypopygus* (*H*. *lepturus*, MCP 44755, 1, 30.0 mm); *Microsternarchus* (*M*. *bilineatus*, MCP 44653, 1, 23.0 mm); *Rhamphichthys* (*R*. *marmoratus*, MCP 44604 (WC01.020195), 43.4 mm).

16. *Occurrence of lateral ethmoid*. (0) absent; (1) present (CI = 0.20; RI = 0.71).

The lateral ethmoid is absent (state 0) in *Brachyhypopomus* sp. “batesi”, *B*. sp. “benjamini”, *B*. *bombilla*, *B*. *jureiae*, *B*. sp. “menezesi”, *B*. sp. “provenzanoi”, *B*. sp. “regani”, and *B*. sp. “sullivani”, and also in *Gymnorhamphichthys*, *Gymnotus*, *Hypopygus*, *Microsternarchus*, *Racenisia*, *Rhamphichthys*, and *Steatogenys*. The lateral ethmoid is present (state 1; Mago-Leccia [51]: 58, fig 11) in all species of *Brachyhypopomus* except *B*. sp. “batesi”, *B*. sp. “benjamini”, *B*. *bombilla*, *B*. *jureiae*, *B*. sp. “menezesi”, *B*. sp. “provenzanoi”, *B*. sp. “regani”, and *B*. sp. “sullivani”, and is also present (state 1) in *Hypopomus* and *Sternopygus*.

17. *Shape of lateral ethmoid*. (0) distinctly wider at ventral and dorsal portions, narrow and tube-shaped in mid portion; (1) tube-shaped and narrow along entire length (CI = 0.33; RI = 0.80).

In the Rhamphichthyoidea, the lateral ethmoid, where present, has two distinct forms. It is narrow and tube-shaped in its mid-section but distinctly wider at its ventral and dorsal portions, resembling a bow-tie, its width reaching approximately 50% of its length (state 0; Mago-Leccia [51]: 55, fig 6) in *Brachyhypopomus bennetti*, *B*. *brevirostris*, *B*. *diazi*, *B*. *draco*, *B*. *janeiroensis*, *B*. *occidentalis*, *B*. sp. “palenque”, and *B*. *walteri*. The lateral ethmoid exhibits a similar form in *Hypopomus*, and *Sternopygus*. The lateral ethmoid is smaller and is tube-shaped along its length, its width not exceeding 25% of its length (state 1) in *Brachyhypopomus* sp. “alberti”, *B*. sp. “arrayae”, *B*. *beebei*, *B*. sp. “belindae”, *B*. *bullocki*, *B*. sp. “cunia”, *B*. sp. “flavipomus”, *B*. *gauderio*, *B*. sp. “hamiltoni”, *B*. sp. “hendersoni”, *B*. *pinnicaudatus*, and *B*. sp. “verdii”. This character is not applicable to taxa in which the lateral ethmoid is absent, i.e. *Brachyhypopomus* sp. “batesi”, *B*. sp. “benjamini”, *B*. *bombilla*, *B*. *jureiae*, *B*. sp. “menezesi”, *B*. sp. “provenzanoi”, and *B*. sp. “sullivani”, and *Gymnorhamphichthys*, *Gymnotus*, *Hypopygus*, *Microsternarchus*, *Racenisia*, *Rhamphichthys*, and *Steatogenys* (see Character 16).

18. *Association of orbitosphenoid and parasphenoid*. (0) ventral portion of orbitosphenoid contacting dorsal region of parasphenoid; (1) ventral portion of orbitosphenoid not contacting dorsal region of parasphenoid (CI = 1.00; RI = 1.00).

The ventral portion of the orbitosphenoid contacts the dorsal surface of the parasphenoid (state 0; Albert et al. [53]: 385, fig 4; Albert & Fink [57]: 88, fig 4) in all species of *Brachyhypopomus* except *B*. sp. “cunia” and *B*. sp. “hendersoni”. It also contacts the dorsal surface of the parasphenoid (state 0) in all outgroups. The ventral portion of the orbitosphenoid does not contact the dorsal surface of the parasphenoid (state 1) in *Brachyhypopomus* sp. “cunia” and *B*. sp. “hendersoni”.

#### Branchial arch

19. *Form of interhyal*. (0) uneven, non-cone-like shape, the anterior portion approximately the same width as the posterior portion; (1) cone-like, posterior portion distinctly wider than the anterior portion (CI = 0.17; RI = 0.62).

**Fig 11:**
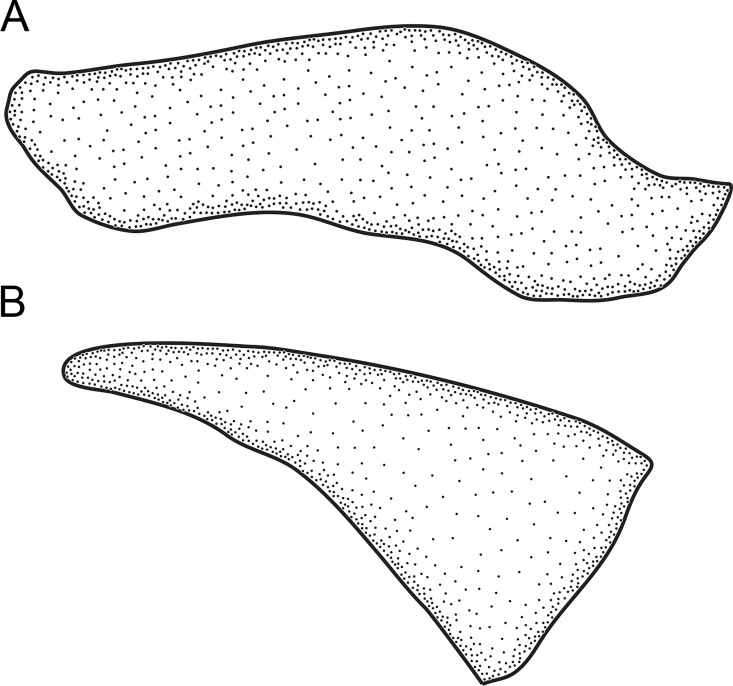
Interhyal. In: (A) *Brachyhypopomus diazi*, UF 174333 (WC06.210304), paraneotype, immature 132 mm TL. (B) *Brachyhypopomus* sp. “alberti”, UMSS 07042 (WC43.070707), paratype, 84 mm TL. Left side, lateral view. Anterior to left.

The interhyal exhibits an uneven, non-cone-like shape, the anterior portion approximately the same width as the posterior portion (state 0; Fig 11A) in *Brachyhypopomus* sp. “batesi”, *B*. sp. “belindae”, *B*. sp. “benjamini”, *B*. *brevirostris*, *B*. sp. “cunia”, *B*. *diazi*, *B*. *draco*, *B*. sp. “flavipomus”, *B*. sp. “hendersoni”, *B*. *janeiroensis*, *B*. *jureiae*, *B*. *occidentalis*, *B*. sp. “palenque”, and *B*. sp. “provenzanoi”, and in all outgroups except *Gymnotus*. The interhyal is cone-shaped (state 1; Fig 11B) in *B*. sp. “alberti”, *B*. sp. “arrayae”, *B*. *beebei*, *B*. *bennetti*, *B*. *bombilla*, *B*. *bullocki*, *B*. *gauderio*, *B*. sp. “hamiltoni”, *B*. sp. “menezesi”, *B*. *pinnicaud*atus, *B*. sp. “regani”, *B*. sp. “sullivani”, *B*. sp. “verdii”, and *B*. *walteri*. This character is not applicable to *Gymnotus*, because the interhyal adopted neither of these two forms in the examined species, *G*. *jonasi*.

20. *Form of anterior extension in cone-like interhyal*. (0) straight; (1) curved (CI = 1.00; RI = 1.00).

Where the anterior extension of the interhyal is cone-like (see Character 19), the dorsal and ventral edges are straight (state 0) in *Brachyhypopomus beebei*, *B*. *bennetti*, *B*. *bombilla*, *B*. *bullocki*, *B*. *gauderio*, *B*. sp. “hamiltoni”, *B*. sp. “menezesi”, *B*. *pinnicaudatus*, *B*. sp. “regani”, *B*. sp. “sullivani”, *B*. sp. “verdii”, and *B*. *walteri*. In contrast, the dorsal and ventral edges of the cone-like portion of the interhyal are curved (convex dorsally, concave ventrally) (state 1, Fig 11B) in *B*. sp. “alberti” and *B*. sp. “arrayae”. This character is not applicable to taxa in which interhyal is an uneven, slender, tube-like structure: i.e. *Brachyhypopomus* sp. “batesi”, *B*. sp. “belindae”, *B*. sp. “benjamini”, *B*. *brevirostris*, *B*. sp. “cunia”, *B*. *diazi*, *B*. *draco*, *B*. *flaviopomus*, *B*. sp. “hendersoni”, *B*. *janeiroensis*, *B*. *jureiae*, *B*. *occidentalis*, *B*. sp. “palenque”, and *B*. sp. “provenzanoi”, and in *Gymnorhamphichthys*, *Hypopomus*, *Hypopygus*, *Microsternarchus*, *Racenisia*, *Rhamphichthys*, *Steatogenys*, and *Sternopygus* (see Character 19), nor is this character applicable to *Gymnotus* (see Character 19 for rationale).

21. *Width of tube-like interhyal*. (0) broad; (1) narrow (CI = 0.25; RI = 0.00).

Where the interhyal is an uneven, slender, tube-like structure (see Character 19), it is expanded laterally into a broad structure (state 0) in *Brachyhypopomus* sp. “batesi”, *B*. sp. “belindae”, *B*. *brevirostris*, *B*. *diazi*, *B*. *draco*, *B*. sp. “flavipomus”, *B*. sp. “hendersoni”, *B*. *janeiroensis*, *B*. *jureiae*, *B*. *occidentalis*, *B*. sp. “palenque”, and *B*. sp. “provenzanoi”, and in *Hypopomus*, *Microsternarchus*, *Racenisia*, *Rhamphichthys*, *Steatogenys*, and *Sternopygus*. The tube-like interhyal is narrow, without expansion (state 1) in *Brachyhypopomus* sp. “benjamini” and *B*. sp. “cunia”, and in *Gymnorhamphichthys* and *Hypopygus*. This character is not applicable to taxa possessing a cone-like anterior extension of the interhyal, i.e. *B*. sp. “alberti”, *B*. sp. “arrayae”, *B*. *beebei*, *B*. *bennetti*, *B*. *bombilla*, *B*. *bullocki*, *B*. *gauderio*, *B*. sp. “hamiltoni”, *B*. sp. “menezesi”, *B*. *pinnicaudatus*, *B*. sp. “regani”, *B*. sp. “sullivani”, *B*. sp. “verdii”, and *B*. *walteri* (see Character 19), nor is this character applicable to *Gymnotus* (see Character 19 for rationale).

22. *Occurrence of first branchiostegal ray*. (0) present; (1) absent (CI = 0.09; RI = 0.41).

The homology of the branchiostegal rays in gymnotiforms was discussed by de Santana & Vari [29]. The first, anterior-most, of five branchiostegal rays, which is attached to the anterior portion of the ceratohyal, is present (state 0; Mago-Leccia [19]: 175, fig 77) in *Brachyhypopomus* sp. “arrayae”, *B*. *bennetti*, *B*. *bombilla*, *B*. *diazi*, *B*. *draco*, *B*. *gauderio*, *B*. sp. “hamiltoni”, *B*. *occidentalis*, *B*. sp. “palenque”, *B*. *pinnicaudatus*, *B*. sp. “regani”, *B*. sp. “sullivani”, and *B*. *walteri*, and in *Gymnorhamphichthys*, *Hypopomus*, *Racenisia*, *Rhamphichthys*, *Steatogenys*, and *Sternopygus*. The first of five branchiostegal ray is absent (state 1) in *B*. sp. “alberti”, *B*. sp. “batesi”, *B*. *beebei*, *B*. sp. “belindae”, *B*. sp. “benjamini”, *B*. *brevirostris*, *B*. *bullocki*, *B*. sp. “cunia”, *B*. sp. “flavipomus”, *B*. sp. “hendersoni”, *B*. *janeiroensis*, *B*. *jureiae*, *B*. sp. “menezesi”, *B*. sp. “provenzanoi”, and *B*. sp. “verdii”, and in *Gymnotus*, *Hypopygus* and *Microsternarchus*.

23. *Relative width of first branchiostegal ray*. (0) first branchiostegal ray approximately as wide as third ray; (1) first branchiostegal ray distinctly narrower than third ray (CI = 0.29; RI = 0.38).

The first branchiostegal ray is approximately as wide as the third branchiostegal ray (state 0) in *Brachyhypopomus* sp. “arrayae”, *B*. *draco*, *B*. *occidentalis*, *B*. sp. “palenque”, and *B*. sp. “sullivani”, and in *Gymnorhamphichthys*, *Hypopomus*, *Rhamphichthys*, and *Sternopygus*. The first branchiostegal is distinctly narrower than the third branchiostegal ray (state 1) in *B*. *bennetti*, *B*. *bombilla*, *B*. *diazi*, *B*. *gauderio*, *B*. sp. “hamiltoni”, *B*. *pinnicaudatus*, and *B*. *walteri*, and in *Racenisia* and *Steatogenys*. This character is polymorphic (state 0 and 1) in *B*. sp. “regani”. This character is not applicable to taxa in which the first branchiostegal ray is absent, i.e. *B*. sp. “alberti”, *B*. sp. “batesi”, *B*. *beebei*, *B*. sp. “belindae”, *B*. sp. “benjamini”, *B*. *brevirostris*, *B*. *bullocki*, *B*. sp. “cunia”, *B*. sp. “flavipomus”, *B*. sp. “hendersoni”, *B*. *janeiroensis*, *B*. *jureiae*, *B*. sp. “menezesi”, *B*. sp. “provenzanoi”, and *B*. sp. “verdii”, and in *Gymnotus*, *Hypopygus* and *Microsternarchus* (see Character 22).

24. *Relative length of first branchiostegal ray*. (0) first branchiostegal ray approximately the same length as the second branchiostegal ray; (1) first branchiostegal ray approximately half the length as the second branchiostegal ray (CI = 0.25; RI = 0.00).

The first branchiostegal ray is approximately the same length as the second branchiostegal ray (state 0) in *Brachyhypopomus* sp. “arrayae”, *B*. *bennetti*, *B*. *diazi*, *B*. *draco*, *B*. *gauderio*, *B*. *pinnicaudatus*, *B*. sp. “sullivani”, and *B*. *walteri*, and in *Gymnorhamphichthys*, *Hypopomus*, *Racenisia*, *Rhamphichthys*, and *Sternopygus*. The first branchiostegal ray is approximately half the length of the second branchiostegal ray (state 1) in *B*. *bombilla*, *B*. *occidentalis*, *B*. sp. “palenque”, and *B*. sp. “regani”, and in *Steatogenys*. This character is not applicable to taxa in which the first branchiostegal ray is absent, i.e. *B*. sp. “alberti”, *B*. sp. “batesi”, *B*. *beebei*, *B*. sp. “belindae”, *B*. sp. “benjamini”, *B*. *brevirostris*, *B*. *bullocki*, *B*. sp. “cunia”, *B*. sp. “flavipomus”, *B*. sp. “hamiltoni”, *B*. sp. “hendersoni”, *B*. *janeiroensis*, *B*. *jureiae*, *B*. sp. “menezesi”, *B*. sp. “provenzanoi”, and *B*. sp. “verdii”, and in *Gymnotus*, *Hypopygus* and *Microsternarchus* (see Character 22).

25. *Relative size of basihyal*. (0) basihyal smaller than first ceratohyal; (1) large, approximately same size of first ceratohyal (CI = 0.17; RI = 0.50).

The basihyal is clearly smaller than the first ceratohyal (state 0; Mago-Leccia [51]: 61, fig 17; Fernandes et al. [18]: 104, fig 7) in *B*. *bullocki*, *B*. *draco*, *B*. sp. “menezesi”, and *B*. sp. “regani”, and in *Gymnotus*, *Hypopygus*, *Microsternarchus*, *Racenisia*, *Steatogenys*, and *Sternopygus*. The basihyal is approximately the same size as the first ceratohyal (state 1) in all species of *Brachyhypopomus* except *B*. *bullocki*, *B*. *draco*, *B*. sp. “menezesi”, and *B*. sp. “regani”, and is also approximately the same size as the first ceratohyal (state 1) in *Gymnorhamphichthys*, *Hypopomus*, and *Rhamphichthys*.

26. *Occurrence of medial bridge on posterior portion of basihyal*. (0) present; (1) absent (CI = 0.14; RI = 0.40).

A medial bridge on the posterior portion of the basihyal is present in many gymnotiforms (state 0, see Triques [32]: 109, figs. 12–13). We noted the presence of this character in *Brachyhypopomus* sp. “alberti”, *B*. *beebei*, *B*. sp. “belindae”, *B*. *bennetti*, *B*. *brevirostris*, *B*. *bullocki*, *B*. *diazi*, *B*. *draco*, *B*. sp. “hamiltoni”, *B*. *janeiroensis*, *B*. *jureiae*, *B*. *occidentalis*, *B*. sp. “palenque”, *B*. *pinnicaudatus*, *B*. sp. “provenzanoi”, *B*. sp. “sullivani”, *B*. sp. “verdii”, and *B*. *walteri*, and in *Gymnorhamphichthys*, *Gymnotus*, *Hypopomus*, *Hypopygus*, *Microsternarchus*, *Rhamphichthys*, *Steatogenys*, and *Sternopygus*. This process is absent (state 1; de Santana & Vari [29]: 250, fig 15; Fernandes et al. [18]: 104, fig 7) in *Brachyhypopomus* sp. “arrayae”, *B*. sp. “batesi”, *B*. sp. “benjamini”, *B*. *bombilla*, *B*. sp. “cunia”, *B*. sp. “flavipomus”, *B*. *gauderio*, *B*. sp. “hendersoni”, *B*. sp. “menezesi”, and *B*. sp. “regani”, and in *Racenisia*.

27. *Ossification of first basibranchial*. (0) unossified; (1) ossified (CI = 1.00; RI = 0.00).

The homology of the first basibranchial in gymnotiforms is disputed; see for example Triques [32] and Hilton et al., [30]. The cartilaginous or ossified first basibranchial is located ventral to the basihyal, between the first hypobranchials. We noted that the first basibranchial is present and cartilaginous (state 0; de La Hoz & Chardon [58]: 30–31, figs. 18–19) in all species of *Brachyhypopomus* except *B*. *brevirostris*, and is also present and cartilaginous (state 0) in all outgroups. In contrast, the first basibranchial is ossified (state 1) in *B*. *brevirostris*.

28. *Form of second basibranchial*. (0) funnel-shaped; (1) arrow-shaped (CI = 0.25; RI = 0.25).

The second basibranchial is funnel-shaped (state 0; de Santana & Vari [29]: 251, fig 16) in *Brachyhypopomus* sp. “benjamini”, and *B*. sp. “provenzanoi”, and in *Microsternarchus*, *Racenisia*, and *Steatogenys*. In contrast, the second basibranchial is arrow-shaped (state 1; de Santana & Vari [29]: 252, fig 17) in all species of *Brachyhypopomus* except *B*. sp. “benjamini” and *B*. sp. “provenzanoi”, and is also arrow-shaped (state 1) in *Gymnorhamphichthys*, *Gymnotus*, *Hypopomus*, *Hypopygus*, *Rhamphichthys*, and *Sternopygus*.

29. *Ossification of third basibranchial*. (0) ossified; (1) unossified (CI = 0.50; RI = 0.50).

The third basibranchial is ossified (state 0) in all species of *Brachyhypopomus* except *B*. sp. “benjamini”, and is also ossified (state 0) in *Gymnorhamphichthys*, *Gymnotus*, *Hypopomus*, *Hypopygus*, *Rhamphichthys*, *Steatogenys*, and *Sternopygus*. The third basibranchial is cartilaginous (state 1) in *B*. sp. “benjamini”, and in *Microsternarchus* and *Racenisia*.

30. *Ossification of fourth basibranchial*. (0) unossified; (1) ossified (CI = 0.50; RI = 0.00).

The fourth basibranchial is unossified (state 0) in all species of *Brachyhypopomus* except *B*. sp. “belindae”, *B*. *brevirostris*, and *B*. sp. “hamiltoni”, and is also unossified (state 0) in all outgroups. The fourth basibranchial is ossified (state 1) in *B*. sp. “belindae”, *B*. *brevirostris*, and *B*. sp. “hamiltoni”.

31. *Occurrence of gill rakers*. (0) present; (1) absent (CI = 0.17; RI = 0.50).

The gill rakers are present (state 0) in *Brachyhypopomus* sp. “batesi”, *B*. *beebei*, *B*. *bennetti*, *B*. *bombilla*, *B*. *brevirostris*, *B*. *bullocki*, *B*. sp. “cunia”, *B*. *diazi*, *B*. *draco*, *B*. sp. “hendersoni”, *B*. *janeiroensis*, *B*. *jureiae*, *B*. sp. “menezesi”, *B*. *occidentalis*, *B*. sp. “palenque”, *B*. sp. “regani”, *B*. sp. “sullivani”, and, *B*. *walteri*, and in *Gymnorhamphichthys*, *Gymnotus*, *Hypopomus*, *Hypopygus*, *Rhamphichthys*, *Steatogenys*, and *Sternopygus*. The gill rakers are absent (state 1) in *B*. sp. “alberti”, *B*. sp. “arrayae”, *B*. sp. “belindae”, *B*. sp. “benjamini”, *B*. sp. “flavipomus”, *B*. *gauderio*, *B*. sp. “hamiltoni”, *B*. *pinnicaudatus*, *B*. sp. “provenzanoi”, and *B*. sp. “verdii”, and in *Microsternarchus* and *Racenisia*.

32. *Form of gill rakers*. (0) crown of teeth; (1) funnel-like (CI = 0.33; RI = 0.67).

The gill rakers are present in the form of a crown with small teeth (state 0) in *Gymnorhamphichthys*, *Gymnotus*, *Hypopomus*, *Rhamphichthys*, *Steatogenys*, and *Sternopygus*. The gill rakers are funnel-shaped (state 1) in all species of *Brachyhypopomus* that possess gill rakers: *Brachyhypopomus* sp. “batesi”, *B*. *beebei*, *B*. *bennetti*, *B*. *bombilla*, *B*. *brevirostris*, *B*. *bullocki*, *B*. sp. “cunia”, *B*. *diazi*, *B*. *draco*, *B*. sp. “hendersoni”, *B*. *janeiroensis*, *B*. *jureiae*, *B*. sp. “menezesi”, *B*. *occidentalis*, *B*. sp. “palenque”, *B*. sp. “regani”, *B*. sp. “sullivani”, and, *B*. *walteri*, and in *Hypopygus*. This character is not applicable to taxa in which the gill rakers are absent, i.e. *B*. sp. “alberti”, *B*. sp. “arrayae”, *B*. sp. “belindae”, *B*. sp. “benjamini”, *B*. sp. “flavipomus”, *B*. *gauderio*, *B*. sp. “hamiltoni”, *B*. *pinnicaudatus*, *B*. sp. “provenzanoi”, and *B*. sp. “verdii”, and *Microsternarchus* and *Racenisia* (see Character 31).

#### Pectoral girdle

33. *Number of proximal radials*. (0) present; (1) absent (CI = 0.14; RI = 0.54).

**Fig 12:**
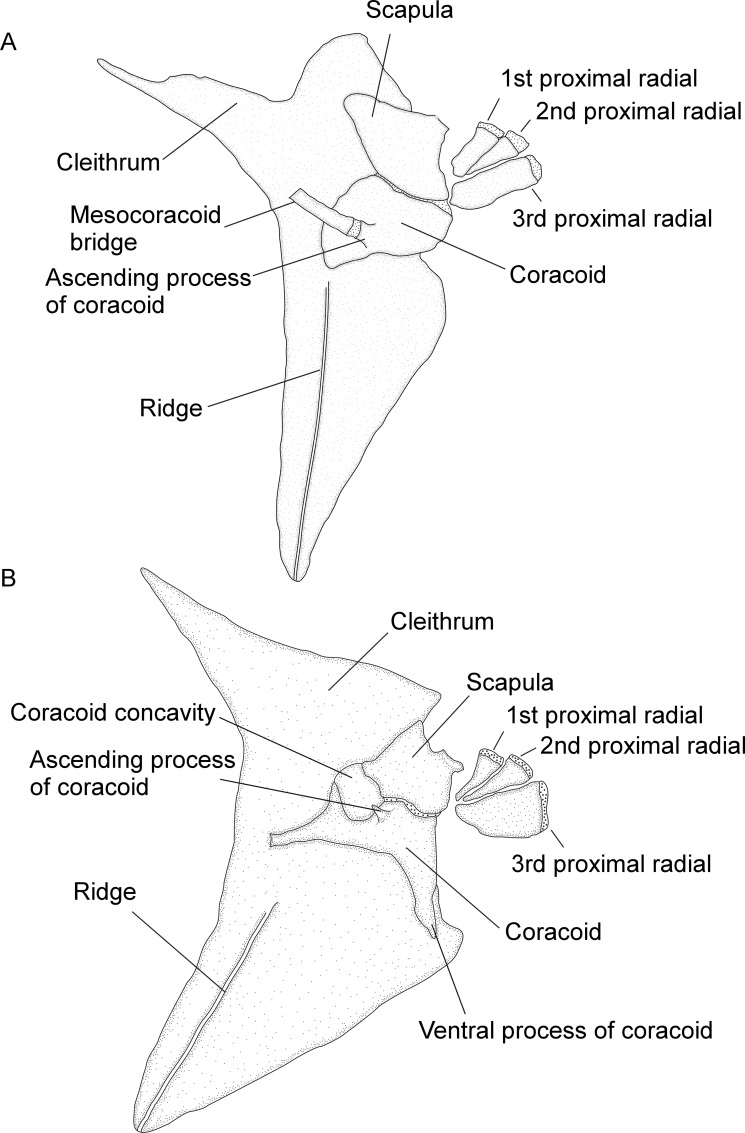
Pectoral girdle. In: (A). *Brachyhypopomus* sp. “hendersoni”. MCP 45432 (WC05.130799), male, 161 mm TL. Left side, medial view. Anterior to left. (B). *Brachyhypopomus walteri*. MCP 44649, (WC04.020698), male, 185 mm TL. Left side, medial view. Anterior to left.

The presence of four pectoral proximal radials is the typical condition in gymnotiforms. Four pectoral radials are present (state 0; e.g. Albert [20]: 9, fig 4a,b; 48, fig 33; Albert et al. [53]: 393, fig 15; Crampton et al. [59]: 252, fig 6; Crampton et al. [60], 129, fig 8; de Santana & Crampton [49]: 1112, figs. 10–11) in *Brachyhypopomus brevirostris*, *B*. *diazi*, *B*. *janeiroensis*, *B*. *jureiae*, *B*. *occidentalis*, *B*. sp. “palenque”, *B*. sp. “sullivani”, and *B*. sp. “verdii” (note in the following species the third and fourth proximals are frequently partially fused in larger specimens but seldom in small specimens < ca. 60 mm, and this fusion is apparent from a continual line along the midline of the fused bones: *B*. *diazi*, *B*. *jureiae*, *B*. *occidentalis*, *B*. sp. “palenque”). Four pectoral radials are also present (state 0) in *Gymnorhamphichthys*, *Gymnotus*, *Hypopomus*, *Hypopygus*, *Rhamphichthys*, and *Sternopygus*. Only three proximal radials are present (state 1; Fig 12A and 12B; Lundberg & Mago-Leccia [55]: 61, fig 7c,d; Fernandes et al. [18]: 106, fig 10) in all species of *Brachyhypopomus* except *B*. *brevirostris*, *B*. *diazi*, *B*. *janeiroensis*, *B*. *jureiae*, *B*. *occidentalis*, *B*. sp. “palenque”, *B*. sp. “sullivani”, and *B*. sp. “verdii”. Only three proximal radials are present (state 1) also in *Microsternarchus*, *Racenisia*, and *Steatogenys*. In *Brachyhypopomus* specimens coded as state 1 we found no evidence for partial or total fusion of the third and fourth radials in the form of a boundary along the midline of the third radial, or via the presence of four radials in early ontogeny. Where only three proximal radials are present, the last one occupies the position normally occupied by the fourth proximal radial. This led us to speculate that the derived condition involves a loss of the fourth (not third) proximal radial.

34. *Association of the third and fourth proximal radials*. (0) third and fourth proximal radials clearly separated; (1) third and fourth proximal radials partially fused (CI = 0.50; RI = 0.67).

The third and fourth proximal radials are clearly separated (state 0; Mago-Leccia [51], 66, fig 27) in *Brachyhypopomus brevirostris*, *B*. *janeiroensis*, *B*. sp. “sullivani”, and *B*. sp. “verdii”, and in *Gymnorhamphichthys*, *Gymnotus*, *Hypopomus*, *Hypopygus*, *Rhamphichthys*, and *Sternopygus*. The third and fourth proximal radials are partially fused (state 1; Mago-Leccia [51]: 60, fig 14; Lundberg & Mago-Leccia [55]: 61, fig 7a, b) in *Brachyhypopomus diazi*, *B*. *jureiae*, *B*. *occidentalis*, and *B*. sp. “palenque”. This character is not applicable to taxa in which only three proximal radials are present, i.e. in all species of *Brachyhypopomus* except *B*. *brevirostris*, *B*. *diazi*, *B*. *janeiroensis*, *B*. *jureiae*, *B*. *occidentalis*, *B*. sp. “palenque”, *B*. sp. “sullivani”, and *B*. sp. “verdii”, and also in *Microsternarchus*, *Racenisia*, and *Steatogenys* (see Character 33).

35. *Association of anterior portion of first through third proximal radials*. (0) anterior portion of first through third proximal radials separated in adults; (1) anterior portion of first through third proximal radials in contact with each other in adults (CI = 0.33; RI = 0.50).

The anterior portions of the first through third proximal radials are completely separated (state 0) in all species of *Brachyhypopomus* except *B*. *bennetti*, *B*. sp. “cunia”, *B*. sp. “hamiltoni”, *B*. sp. “hendersoni”, and *B*. *walteri*, and are also completely separated (state 0) in all outgroups. The anterior portions of the first through third proximal radials are in contact with each other (state 1) in adults (but not small juveniles) of *B*. *bennetti*, *B*. sp. “cunia”, B. sp. “hamiltoni”, *B*. sp. “hendersoni”, and *B*. *walteri*.

36. *Occurrence of abrupt concavity on coracoid*. (0) absent; (1) present (CI = 0.33; RI = 0.50).

The coracoid is continuous, with no distinct concavity in the dorsoposterior area (state 0; Fig 12A; Lundberg & Mago-Leccia [55]: 61, fig 7b,c,d) in all species of *Brachyhypopomus* except *B*. *beebei*, *B*. *bennetti*, *B*. *gauderio*, *B*. *pinnicaudatus*, and *B*. *walteri*, and is also continuous, with no foramen (state 0) in all outgroups. An abrupt concavity delineated posterolaterally by the cleithrum is present on the dorsoposterior portion of the coracoid (state 1; Fig 12B) in *B*. *beebei*, *B*. *bennetti*, *B*. *gauderio*, *B*. *pinnicaudatus*, and *B*. *walteri*.

37. *Occurrence of mesocoracoid bridge*. (0) absent; (1) present (CI = 0.25; RI = 0.50).

A mesocoracoid bridge is absent (state 0; Fig 12B; Hulen et al. [61]: 423, fig 16) in all *Brachyhypopomus* species except *B*. *brevirostris*, *B*. *bullocki*, *B*. sp. “cunia”, and *B*. sp. “hendersoni”, and is also absent (state 0) in the outgroups *Gymnorhamphichthys*, *Gymnotus*, *Hypopygus*, *Microsternarchus*, *Racenisia*, and *Sternopygus*. The mesocoracoid bridge is present (state 1; Fig 12A; Hilton et al. [30]: 20, fig 18) in *B*. *brevirostris*, *B*. *bullocki*, *B*. sp. “cunia”, and *B*. sp. “hendersoni”, and in *Hypopomus*, *Rhamphichthys*, and *Steatogenys*.

38. *Association of mesocoracoid bridge and coracoid*. (0) mesocoracoid bridge not contacting scapula; (1) mesocoracoid bridge contacting scapula (CI = 1.00; RI = 0.00).

The anterior portion of the mesocoracoid bridge contacts the medial portion of the scapula and coracoid (state 0; Hilton et al. [30]: 20, fig 18b) in *Brachyhypopomus brevirostris* and *B*. *bullocki*, and in *Hypopomus*, *Rhamphichthys*, and *Steatogenys*. Conversely, the anterior portion of the mesocoracoid bridge contacts only the medial portion of the coracoid in *B*. sp. “hendersoni” (state 1). This character is not applicable to taxa in which the mesocoracoid bridge is absent, i.e. in all species of *Brachyhypopomus* except *B*. *brevirostris*, *B*. *bullocki*, *B*. *cunia*, and *B*. sp. “hendersoni”, and also in *Gymnorhamphichthys*, *Gymnotus*, *Hypopygus*, *Microsternarchus*, *Racenisia*, and *Sternopygus* (see Character 37).

39. *Occurrence of mesocoracoid bridge process on scapula*. (0) present; (1) absent (CI = 1.00; RI = 1.00).

The scapula contacts the anterior portion of the mesocoracoid bridge via an ascending process. This process is present (state 0; Hilton et al. [30]: 20, fig 18b) in *B*. *brevirostris* and *B*. *bullocki*, and in *Hypopomus*, *Rhamphichthys*, and *Steatogenys*. The ascending process of mesocoracoid bridge contacting the scapula is absent in *B*. sp. “cunia” and *B*. sp. “hendersoni” (stage 1, Fig 12A). This character is not applicable to taxa for which the mesocoracoid bridge is absent, i.e. in all species of *Brachyhypopomus* except *B*. *brevirostris*, *B*. *bullocki*, *B*. sp. “cunia”, and *B*. sp. “hendersoni”, and also in the outgroups *Gymnorhamphichthys*, *Gymnotus*, *Hypopygus*, *Microsternarchus*, *Racenisia*, and *Sternopygus* (see Character 37).

40. *Relative size of posterior tip of mesocoracoid bridge*. (0) posterior tip of mesocoracoid bridge wide; (1) posterior tip of mesocoracoid bridge narrow (CI = 1.00; RI = 1.00).

The posterior tip of the mesocoracoid bridge is wide–twice or more as wide as the mid-portion of the mesocoracoid bridge (state 0; Hilton et al. [30]: 20, fig 18b), in *B*. *brevirostris* and *B*. *bullock*, and in *Hypopomus*, *Rhamphichthys*, and *Steatogenys*. The anterior tip of the mesocoracoid bridge is narrow–of approximately equal width to the mid-portion (state 1; Fig 12A) in *B*. sp. “cunia” and *B*. sp. “hendersoni”. This character is not applicable to taxa for which the mesocoracoid bridge is absent: i.e. in all species of *Brachyhypopomus* except *B*. *brevirostris*, *B*. *bullocki*, *B*. sp. “cunia”, and *B*. sp. “hendersoni”, and also in the following outgroups: *Gymnorhamphichthys*, *Gymnotus*, *Hypopygus*, *Microsternarchus*, *Racenisia*, and *Sternopygus* (see Character 37).

41. *Form of mesocoracoid bridge*. (0) approximately straight in dorsal view; (1) distinctly curved (CI = 1.00; RI = 1.00).

The mesocoracoid bridge is approximately straight in dorsal view (state 0) in *Hypopomus*, *Rhamphichthys*, and *Steatogenys*. It is distinctly curved towards the midline of the body (state 1) in *B*. *brevirostris*, *B*. *bullocki*, *B*. sp. “cunia”, and *B*. sp. “hendersoni”. This character is not applicable to taxa for which the mesocoracoid bridge is absent, i.e. all species of *Brachyhypopomus* except *B*. *brevirostris*, *B*. *bullocki*, *B*. sp. “cunia”, and *B*. sp. “hendersoni”, and also in *Gymnorhamphichthys*, *Gymnotus*, *Hypopygus*, *Microsternarchus*, *Racenisia*, and *Sternopygus* (see Character 37).

42. *Occurrence of ascending process on coracoid*. (0) absent; (1) present (CI = 1.00; RI = 1.00).

A small triangular ascending process on the coracoid, without cartilage, and presumably unrelated to the mesocoracoid bridge process, is absent (state 0) in all species of *Brachyhypopomus* except *B*. *bennetti* and *B*. *walteri*, and is also absent (state 0) in all outgroups. Conversely, this process is present (state 1; Fig 12B) in *B*. *bennetti* and *B*. *walteri*.

43. *Ventral process of coracoid*. (0) present; (1) absent (CI = 1.00; RI = 1.00).

A ventral process of the coracoid is present (state 0; Fig 12B) in all species of *Brachyhypopomus* except *B*. *brevirostris*, *B*. *bullocki*, *B*. sp. “cunia”, and *B*. sp. “hendersoni”, and is also present (state 0) in all outgroups. A ventral process of the coracoid is absent in *B*. *brevirostris*, *B*. *bullocki*, *B*. sp. “cunia”, and *B*. sp. “hendersoni” (state 1; Fig 12A). We noted that the ventral process of the coracoid, where present, is a distinct, narrow, and moderate to long ossification extending posteriorly to the ventral portion of cleithrum (Fig 12B) in all species of *Brachyhypopomus* and in *Steatogenys*. Conversely, the process is wide and relatively short in *Hypopomus*.

44. *Association of posttemporal and supracleithrum*. (0) separate; (1) fused (CI = 1.00; RI = 0.00).

The posttemporal is separate from the supracleithrum (state 0; Mago-Leccia [51]: 66, fig 27; Mago-Leccia [19]: 175, fig 77) in all species of *Brachyhypopomus* except *B*. *pinnicaudatus*, and is also separate from the supracleithrum (state 0) in all outgroups. The posttemporal and supracleithrum are fused (state 1; Mago-Leccia et al. [62]: 8, fig 6) in *B*. *pinnicaudatus*.

#### Squamation

45. *Occurrence of scales on dorsal region of anterior third of body*. (0) scales present; (1) scales absent (CI = 0.25; RI = 0.25).

Small cycloid scales are present on the entire middorsal region (state 0) in all species of *Brachyhypopomus* except *B*. sp. “benjamini” and *B*. sp. “provenzanoi”, and are also present (state 0) in *Gymnotus*, *Hypopomus*, *Hypopygus*, *Rhamphichthys*, *Steatogenys*, and *Sternopygus*. Scales are absent from the entire middorsal region (state 1) in the anterior third of body in *B*. sp. “benjamini” and *B*. sp. “provenzanoi”, and from the entire middorsal region along the body in *Gymnorhamphichthys*, *Microsternarchus*, and *Racenisia*.

#### Upper jaw

46. *Occurrence of teeth on premaxilla*. (0) present at some stage in ontogeny; (1) absent throughout ontogeny (CI = 0.50; RI = 0.67).

Teeth are present on the premaxilla in both juveniles and adults of *Gymnotus* and *Sternopygus* (Albert [20]: 17, fig 6; Mago-Leccia [51]: 65, fig 26; Albert et al. [53]: 388, fig 9), but Albert [20] reported the complete absence of all oral teeth in all the Rhamphichthyoidea. Nonetheless, we noted that teeth are present on the premaxilla (state 0) at some stage in ontogeny in several species of *Brachyhypopomus*. In the following species we found small, needle-like conical teeth on the premaxilla in juvenile specimens: *B*. *bennetti* (n = 1, 73 mm: 5–7 teeth; MCP 46934), *B*. *brevirostris* (n = 1, 47.7 mm, 1 tooth; MCP 44759), *B*. *diazi* (n = 2, 20–80 mm, 3–4 teeth; UF 174333; UF 176888, WC14.020403); *B*. sp. “palenque” (n = 1, 31 mm, 5 teeth; UF 180271, WC08.160404) and *B*. *walteri* (n = 1, 70 mm, 5–7 teeth; MCP 46933). We also found conical needle-like premaxillary teeth in adult males of two species: *B*. *bennetti* (1–4 teeth on each premaxilla), and *B*. *walteri* (1–3 teeth on each premaxilla) (e.g. *B*. *bennetti*: MCP 45359; *B*. *walteri* MCP 44649). Sullivan et al. [63] noted the presence of premaxillary teeth, hitherto unknown in Rhamphichthyoidea, in adults of these two species. They reported 1–5 teeth in *B*. *bennetti*, and "one or more" in *B*. *walteri* (Sullivan et al. [63]: 7, fig 1; ANSP 194025).

Specimens of small juveniles (< ca. 50 mm TL) of *Brachyhypopomus* were only available for the species listed above, and yet all exhibited teeth on the premaxilla. We suspect that this character may be more widespread in the genus but are as yet unable to confirm this. Therefore, we code other species of *Brachyhypopomus* as unknown for this character. We reported the absence of teeth on the premaxilla in both adults and small juveniles (<50 mm TL) (state 1) in the following outgroups: *Hypopygus*, *Microsternarchus*, *Racenisia*, and *Rhamphichthys* (see S1 and S2 appendices for size range of examined cleared and stained specimens). Small juveniles of *Gymnorhamphichthys*, *Hypopomus*, and *Steatogenys* were unavailable for analysis, and are coded unknown.

47. *Form of descending process of maxilla*. (0) broad in adults only, or broad throughout ontogeny; (1) narrow in all ontogenetic stages (CI = 0.33; RI = 0.78).

**Fig 13:**
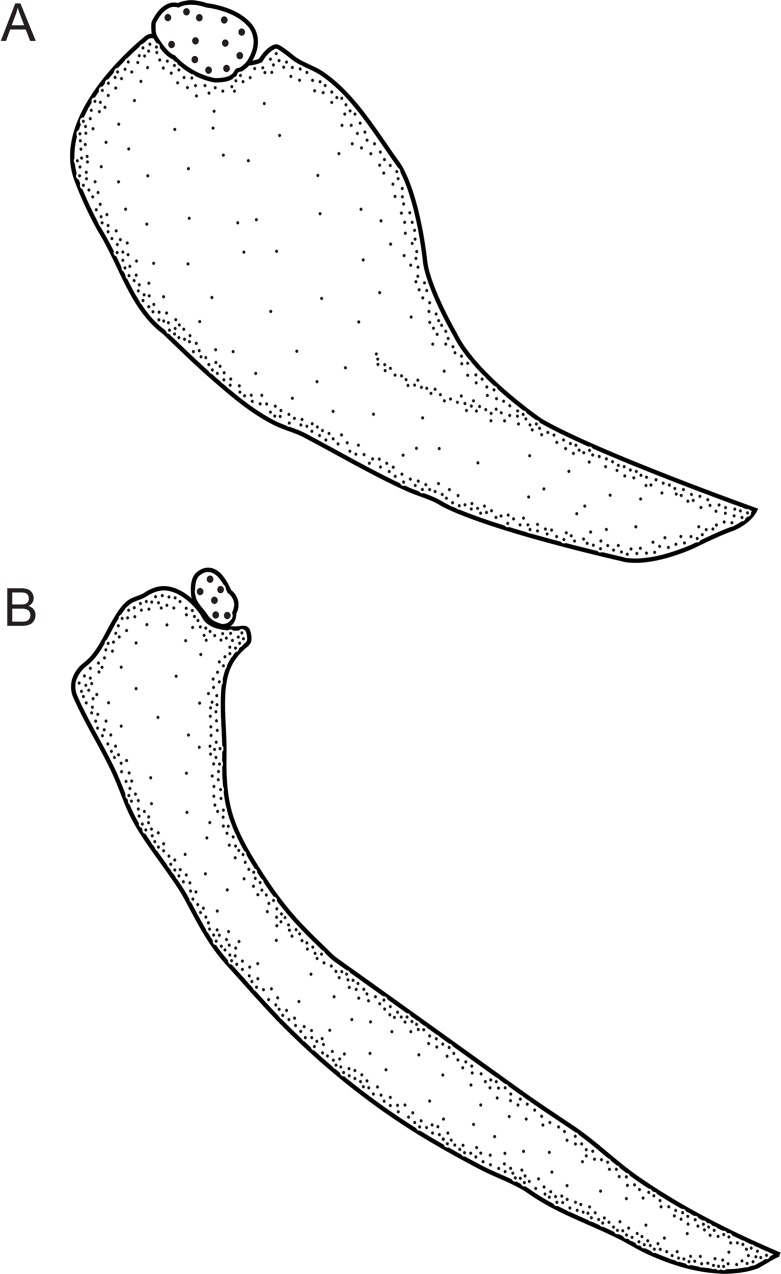
Maxilla. In: (A) *Brachyhypopomus occidentalis* USNM 293152, 156 mm TL. (B) *Brachyhypopomus* sp. “hendersoni” MCP 45432 (WC05.130799), 1, 161 mm TL. Left side, lateral view. Anterior to left.

The blade on the posterior portion of the descending process of maxilla is broad (state 0; Fig 13A) throughout ontogeny in *B*. *bombilla*, *B*. *diazi*, *B*. sp. “menezesi”, *B*. *occidentalis*, *B*. sp. “palenque”, *B*. sp. “regani”, *B*. sp. “sullivani”, and in *Hypopomus*, *Microsternarchus* and *Racenisia*. We noted that the blade on the posterior portion of the descending process of maxilla is broad in adults of many of these taxa, while narrow in juveniles, suggesting that the blade broadens during ontogeny. The posterior portion of the descending process of the maxilla is narrow at all ontogenetic stages (state 1; Fig 13B) in all species of *Brachyhypopomus* except *B*. *bombilla*, *B*. *diazi*, *B*. sp. “menezesi”, *B*. *occidentalis*, *B*. sp. “palenque”, *B*. sp. “regani”, and *B*. sp. “sullivani”, and is also narrow (state 1) in *Gymnorhamphichthys*, *Gymnotus*, *Hypopygus*, *Rhamphichthys*, *Steatogenys*, and *Sternopygus*.

#### Sympletic, hyomandibular, opercular series and related sensory canals

48. *Association of the preopercular sensory canals and preopercle* (multistate). (0) preopercular sensory canals incised in preopercle; (1) preopercular sensory canals independent of preopercle; (2) only posterior-most sensory canal incised in preopercle (CI = 0.40; RI = 0.82).

**Fig 14:**
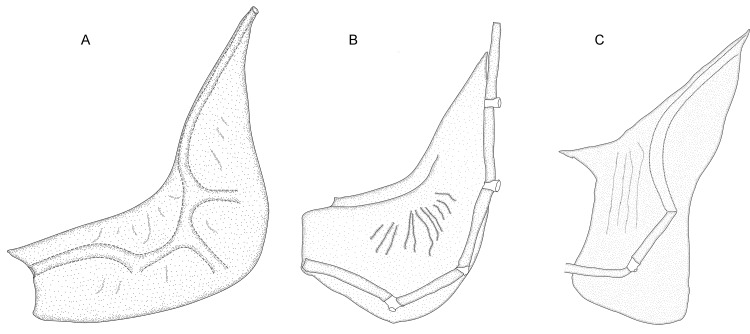
Preopercle and associated sensory canals. In: (A) *B*. *brevirostris*. MCP 44605 (WC06.010596), male, 374 mm TL. Left side, lateral view. Anterior to left. Note the incised preopercular sensory canals (borders highlighted with dashed lines). (B). *B*. *beebei*. MCP 45421 (WC04.160698), male, 201 mm TL. Left side, lateral view. Anterior to left. Note the preopercular sensory canals are completely independent of preopercle (borders highlighted with solid lines). (C) *B*. sp. “sullivani”. MCP 45486 (WC02.221299), male, 101 mm TL. Left side, lateral view. Anterior to left. Note only the posterior-most preopercular sensory canal is incised in preopercle (borders highlighted with dashed lines).

The preopercular sensory canals, when present, are incised in the preopercle (state 0, Fig 14A) in *B*. sp. “batesi”, *B*. sp. “benjamini”, *B*. *bennetti*, *B*. *brevirostris*, *B*. *bullocki*, *B*. sp. “cunia”, *B*. *diazi*, *B*. sp. “hendersoni”, *B*. *occidentalis*, *B*. sp. “provenzanoi”, and *B*. *walteri*, and in *Gymnorhamphichthys*, *Gymnotus*, *Hypopomus*, *Rhamphichthys*, *Steatogenys*, and *Sternopygus*. The preopercular sensory canals are completely independent of the preopercle (state 1, Fig 14B) in *B*. sp. “alberti”, *B*. sp. “arrayae”, *B*. *beebei*, *B*. sp. “belindae”, *B*. *draco*, *B*. sp. “flavipomus”, *B*. *gauderio*, *B*. sp. “hamiltoni”, *B*. *janeiroensis*, *B*. *jureiae*, *B*. sp. “palenque”, *B*. *pinnicaudatus*, and *B*. sp. “verdii”. Only the posterior-most preopercular sensory canal is incised in the preopercle (state 2, Fig 14C) in *B*. *bombilla*, *B*. sp. “menezesi”, *B*. sp. “regani”, and *B*. sp. “sullivani”, and in the *Microsternarchus*. This character is not applicable to *Hypopygus* and *Racenisia*, which do not possess preopercular sensory canals.

49. *Occurrence of hyomandibular dorsal foramen*. (0) absent; (1) present (CI = 1.00; RI = 1.00).

The hyomandibular dorsal foramen is absent (state 0) in all species of *Brachyhypopomus*, and in *Gymnorhamphichthys*, *Gymnotus*, *Hypopomus*, *Microsternarchus*, *Racenisia*, *Rhamphichthys*, and *Sternopygus*. The hyomandibular dorsal foramen is present (state 1) in *Hypopygus* and *Steatogenys*.

#### Lower jaw

50. *Occurrence of teeth on the dentary*. (0) present at some stage in ontogeny; (1) absent throughout ontogeny (CI = 0.50; RI = 0.67).

Mago-Leccia [51] and Albert [20] commented that members of the Rhamphichthyoidea are recognized by the absence of teeth on the oral jaws; although Fernández-Yépez [64] cited the presence of teeth on the dentary of *Microsternarchus bilineatus*. We observed the presence of small conical dentary teeth (state 0) in both juveniles and adults of *Gymnotus* and *Sternopygus*, and in juveniles of (but not adults of) the following four species of *Brachyhypopomus*: *B*. *brevirostris* (n = 1, 47.7 mm TL; MCP 44759), *B*. *diazi* (n = 1, 20 mm TL; UF 174333), *B*. sp. “palenque” (n = 1, 31 mm, 5 teeth; UF 180271, WC08.160404), and *B*. *walteri* (n = 1, 70 mm TL; MCP 46933). As with the presence of premaxillary teeth, we suspect that the presence of teeth on the dentary may be more widespread in post-larval specimens and small juveniles of the genus. Nonetheless, due to the rarity of identifiable small juvenile specimens in collections, we are as yet unable to confirm this. Therefore we coded all species of *Brachyhypopomus* for which small juveniles are currently unavailable as “unknown”. We noted the absence of dentary teeth (state 1) in both adults and small juveniles (< 42 mm) of *Hypopygus*, *Microsternarchus* (contrary to Fernández-Yépez, 1968), *Racenisia*, and *Rhamphichthys* (see S1 and S2 Appendices for size ranges of examined cleared and stained specimens). Small juveniles were not available for *Gymnorhamphichthys*, *Hypopomus*, and *Steatogenys* and we therefore coded these taxa as “unknown”.

51. *Form of dorsoposterior portion of dentary*. (0) dorsoposterior portion of dentary straight and even (1) dorsoposterior portion of dentary uneven, or with hook-like process (CI = 0.40; RI = 0.57).

**Fig 15:**
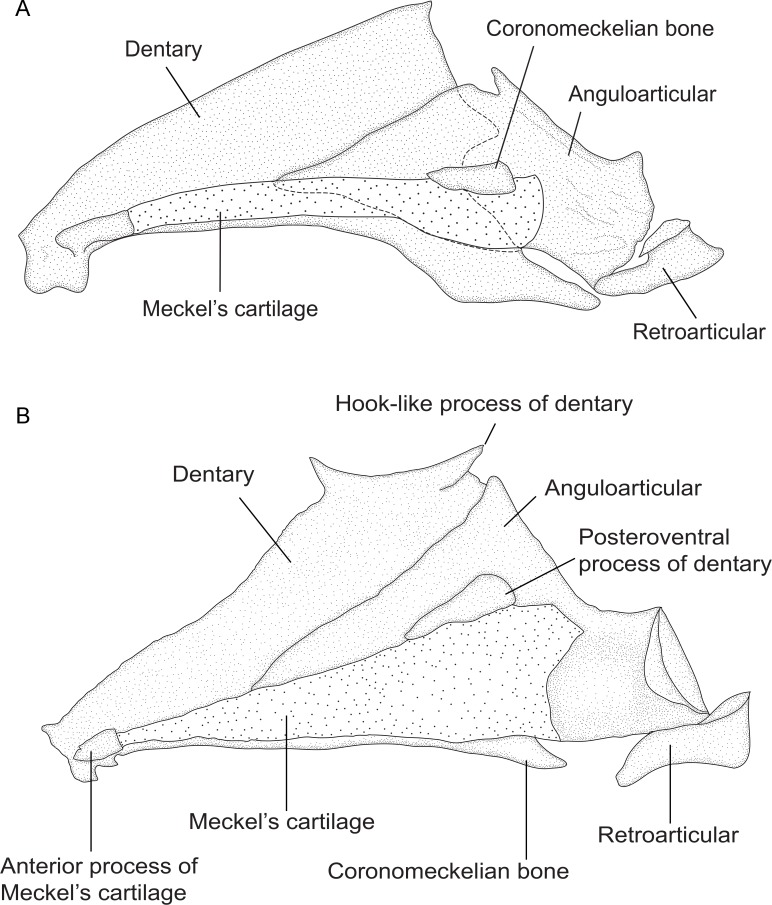
Lower jaw. In: (A) *B*. sp. “hendersoni”. MCP 45432 (WC05.130799), 1, 161 mm TL. Left side, medial view, anterior to left. Note the posterodorsal margin of the dentary is straight and even (Character 51). (B). *B*. sp. “sullivani”. MCP 45486 (WC02.221299), male, 101 mm TL. Right side (inverted), medial view, anterior to left. Note the posterodorsal margin of the dentary is concave, forming a hook-like process (Character 51).

The dorsoposterior portion of the dentary is straight and even (state 0; Fig 15A; Albert et al. [53]: 389, fig 10) in all species of *Brachyhypopomus* except *B*. *diazi*, *B*. sp. “menezesi”, *B*. *occidentalis*, *B*. sp. “palenque”, *B*. sp. “regani”, and *B*. sp. “sullivani”, and is also straight and even (state 0) in *Gymnorhamphichthys*, *Gymnotus*, *Hypopomus*, *Hypopygus*, *Rhamphichthys*, *Steatogenys*, and *Sternopygus*. The dorsoposterior portion of dentary is uneven or has a hook-like process (state 1; Fig 15B) in *B*. *bombilla*, *B*. *diazi*, *B*. sp. “menezesi”, *B*. *occidentalis*, *B*. sp. “palenque”, *B*. sp. “regani”, and *B*. sp. “sullivani”, and in *Microsternarchus* and *Racenisia*. We coded *Hypopygus* as polymorphic for this character because both character states are observed among specimens of *H*. *lepturus*.

#### Weberian apparatus

52. *Occurrence of small independent ossification on supraoccipital*. (0) present; (1) absent (CI = 0.50; RI = 0.83).

**Fig 16:**
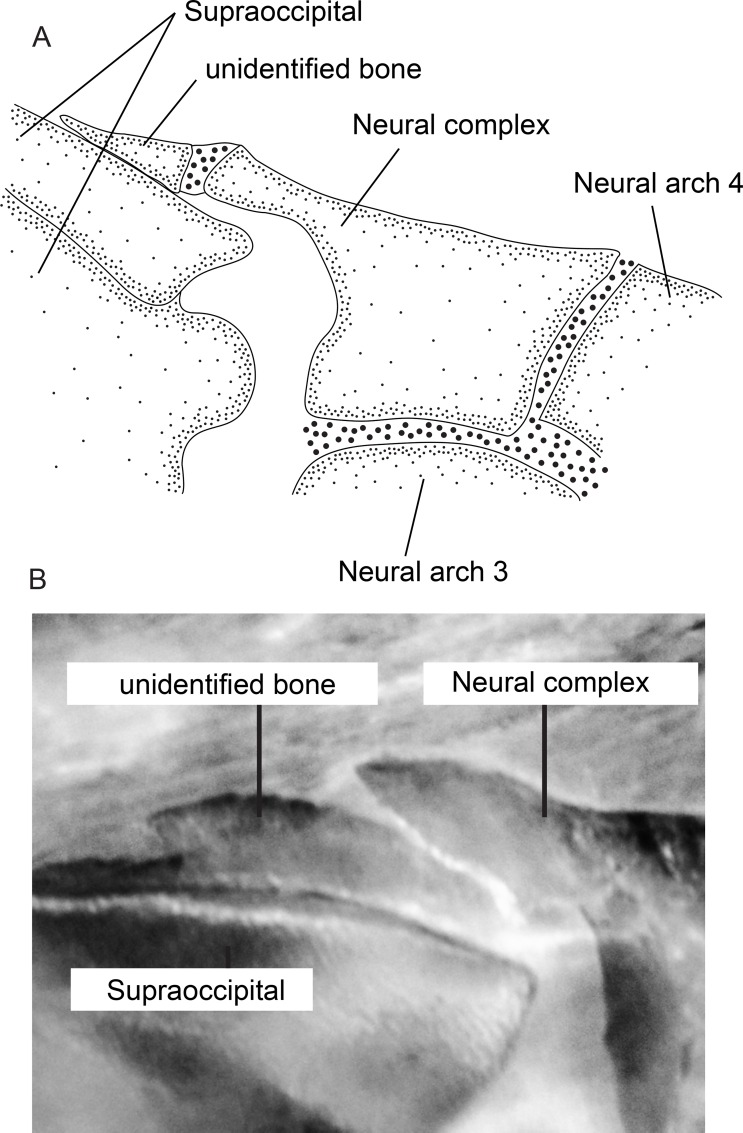
Posterior portion of the neurocranium and anterior portion of the Weberian apparatus. Note the undescribed ossification on the supraoccipital, anterior to the neural complex, in: (A) *Brachyhypopomus beebei* MCP 45450 (WC06.090600), female, 152 mm; (B) *Brachyhypopomus brevirostris* MCP 44605 (WC06.010596), male, 374 mm TL (photograph). Left side, lateral view. Anterior to left.

A small independent, undescribed ossification (bone) located on the supraoccipital, anterior to the neural complex is absent (state 0, Mago-Leccia [51]: 62, fig 18) in most gymnotiform genera, including in *Gymnorhamphichthys*, *Gymnotus*, *Hypopomus*, *Hypopygus*, *Rhamphichthys*, *Steatogenys*, and *Sternopygus*. This ossification is present (state 1; Fig 16A and 16B; Sullivan [24]: 319, fig 51) in all species of *Brachyhypopomus*, and in *Microsternarchus* and *Racenisia*.

53. *Association of supraoccipital with a small un-named bone above supraoccipital*. (0) separate; (1) overlapping (CI = 1.00; RI = 1.00).

The posterior portion of a small independent, undescribed ossification (bone) above the supraoccipital and anterior portion of neural complex is clearly separated from the supraoccipital (state 0; Fig 16A) in all species of *Brachyhypopomus* except *B*. *brevirostris*, *B*. *bullocki*, *B*. sp. “cunia”, and *B*. sp. “hendersoni”, and is also separated from the supraoccipital (state 0) in *Microsternarchus* and *Racenisia*. Conversely, this small bone overlaps with the supraoccipital (state 1; Fig 16B) in *B*. *brevirostris*, *B*. *bullocki*, *B*. sp. “cunia”, and *B*. sp. “hendersoni”. This character is not applicable to taxa for which this small independent ossification is absent, i.e. *Gymnorhamphichthys*, *Gymnotus*, *Hypopomus*, *Hypopygus*, *Rhamphichthys*, *Steatogenys*, and *Sternopygus* (see Character 52).

54. *Association of neural complex and exoccipital*. (0) neural complex not contacting exoccipital; (1) neural complex contacting exoccipital (CI = 0.14; RI = 0.54).

The neural complex does not contact the posterior part of the exoccipital (state 0) in *B*. sp. “batesi”, *B*. sp. “benjamini”, *B*. *bombilla*, *B*. *diazi*, *B*. sp. “flavipomus”, *B*. sp. “menezesi”, *B*. *occidentalis*, *B*. sp. “palenque”, *B*. sp. “provenzanoi”, *B*. sp. “sullivani”, and *B*. sp. “verdii”, and in *Gymnotus*, *Hypopomus*, and *Racenisia*. The anterior portion of the neural complex contacts the posterior part of the exooccipital (state 1) *in B*. sp. “alberti”, *B*. sp. “arrayae”, *B*. *beebei*, *B*. sp. “belindae”, *B*. *bennetti*, *B*. *brevirostris*, *B*. *bullocki*, *B*. sp. “cunia”, *B*. *draco*, *B*. *gauderio*, *B*. sp. “hamiltoni”, *B*. sp. “hendersoni”, *B*. *janeiroensis*, *B*. *jureiae*, *B*. *pinnicaudatus*, *B*. sp. “regani”, and *B*. *walteri* and in *Gymnorhamphichthys*, *Hypopygus*, *Microsternarchus*, *Rhamphichthys*, *Steatogenys*, and *Sternopygus*.

55. *Form of anterior portion of neural complex*. (0) approximately straight; (1) concave (CI = 1.00; RI = 0.00).

The anterior portion of the neural complex is approximately straight and overlaps the surface of the supraoccipital (state 0; Mago-Leccia [51]: 62, fig 18) in all outgroups, and in all species of *Brachyhypopomus* except *B*. sp. “verdii”. Conversely, the anterior portion of the neural complex is distinctly concave and does not overlap the surface of the supraoccipital (state 1) in *Brachyhypopomus* sp. “verdii”.

#### Palatine arch

56. *Association of ascending process on endopterygoid with orbitosphenoid*. (0) not contacting orbitosphenoid; (1) contacting orbitosphenoid (CI = 0.11; RI = 0.50).

**Fig 17:**
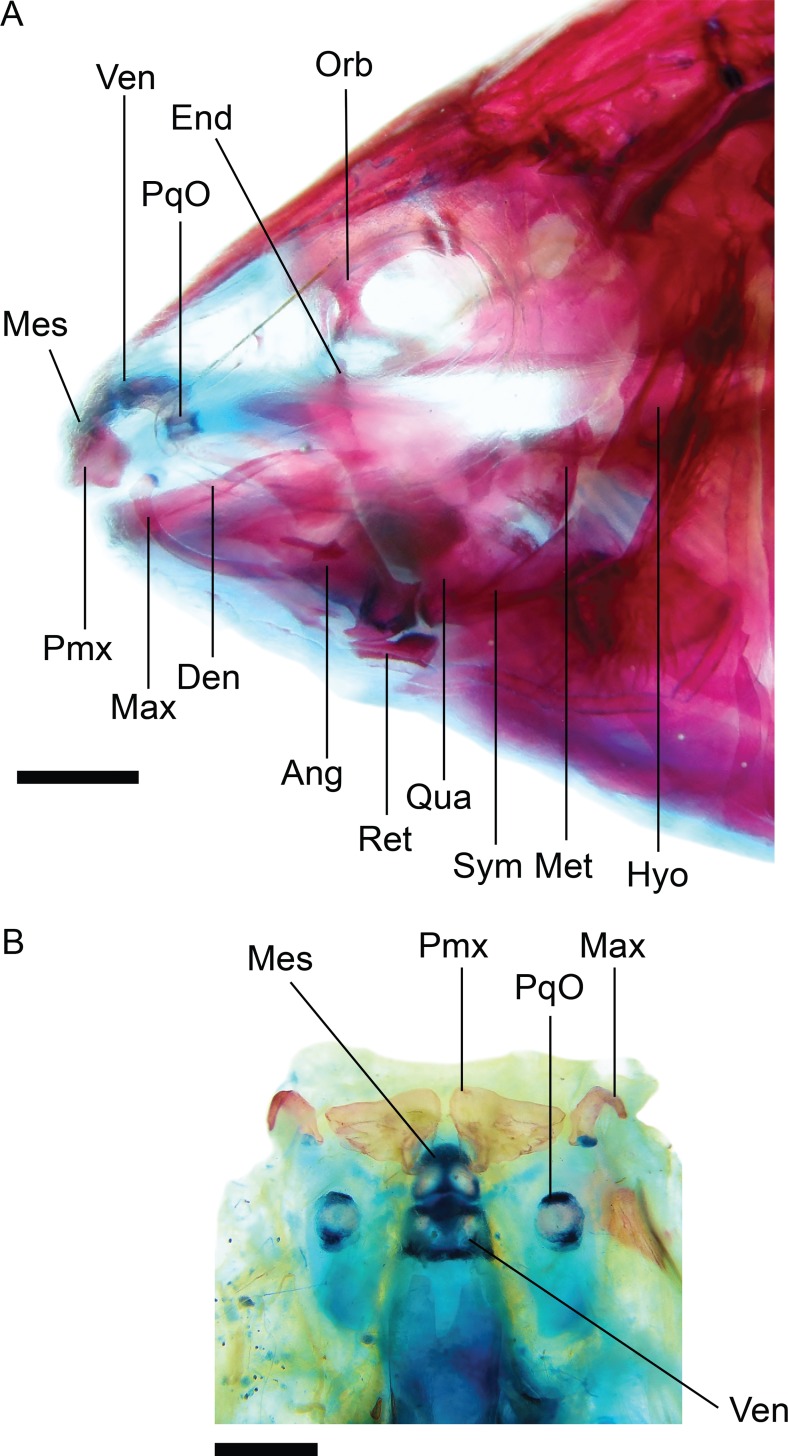
Ossification of the anterior portion of the palatoquadrate cartilage. In photographs of cleared and stained specimens of: (A) *Brachyhypopomus pinnicaudatus*—MCP 45370 (WC03.050497), female, 122 mm (anterior portion of head in lateral view); (B) *Brachyhypopomus beebei*—MCP 45450 (WC06.090600), female, 152 mm (ventral surface of anterior portion of the neurocranium and associated structures). Note the typical disk-like outline of the ossified element in the palatoquadrate cartilage (PqO) in ventral view–B, and rectangular outline in lateral view–A. Note also, in A, the ascending process of the endopterygoid (End) does not form a contact with the orbitosphenoid (Orb). Abbreviations for bones in the jaw and suspensorium are: Pmx—premaxilla; Mes—mesethmoid; Ven—ventral ethmoid; Max—maxilla; Den—dentary; Ang—anguloarticular; Ret—retroarticular; Qua—quadrate; Sym—symplectic; Met—metapterygoid; Hyo—hyomandibula. Scale bar 1 mm.

A small ascending process on the endopterygoid (mesopterygoid of Mago-Leccia [51]: 10, e.g. 57, fig 8) is present in all species of *Brachyhypopomus* (Fig 17A, see end of pointer labeled “End”; Arratia & Schultze [65]: 43, fig 26; de Santana & Crampton [49]: 1104, fig 2), and in *Gymnotus* (Albert et al. [53]: 387, fig 8), *Hypopomus*, *Hypopygus* (de Santana & Crampton [49]: 1105, figs. 3–4), *Microsternarchus*, *Racenisia* (Mago-Leccia [19]: 175, fig 77), *Steatogenys*, and *Sternopygus* (Mago-Leccia [51]: 64, fig 25; Crampton et al. [60]: 129, fig 8; Hulen et al. [61]: 414, fig 7). The ascending process of the endopterygoid is absent in *Gymnorhamphichthys*. This ascending process on the endopterygoid does not contact the orbitosphenoid (state 0; Fig 17A) in *B*. *beebei*, *B*. *brevirostris*, *B*. sp. “cunia”, *B*. *diazi*, *B*. *draco*, *B*. sp. “flavipomus”, *B*. *gauderio*, *B*. *janeiroensis*, *B*. *jureiae*, *B*. *occidentalis*, *B*. sp. “palenque”, *B*. *pinnicaudatus*, and *B*. sp. “verdii”, and in *Gymnotus*, *Hypopomus*, *Hypopygus*, *Microsternarchus*, *Racenisia*, and *Steatogenys*, or is absent (in *Gymnorhamphichthys*). The ascending process on the endopterygoid forms a contact with the orbitosphenoid (state 1) in *Brachyhypopomus* sp. “alberti”, *B*. sp. “arrayae”, *B*. sp. “batesi”, *B*. sp. “belindae”, *B*. sp. “benjamini”, *B*. *bennetti*, *B*. *bombilla*, *B*. *bullocki*, *B*. sp. “hamiltoni”, *B*. sp. “hendersoni”, *B*. sp. “menezesi”, *B*. sp. “provenzanoi”, *B*. sp. “regani”, *B*. sp. “sullivani”, *and B*. *walteri*, and in *Gymnorhamphichthys*, *Rhamphichthys*, and *Sternopygus*.

57. *Ossification of palatoquadrate cartilage in adult specimens*. (0) does not ossify; (1) ossifies (CI = 1.00; RI = 1.00).

The palatoquadrate cartilage does not ossify (state 0; Triques [32]: 100, fig 7; Hilton [30]: 14, fig 11; de Santana & Vari [29]: 239–240, figs. 8–10) in all outgroups. Conversely, a disk-like ossification is present in adults in the anterior portion of the palatoquadrate cartilage–the pars autopalatine of Hilton et al. [30] (state 1; Fig 17A and 17B; Arratia & Schultze [65]: 43, fig 26b; Sullivan [24]: 316, fig 49; Sullivan et al. [63]: 7, fig 1) in all species of *Brachyhypopomus*. Note: the illustration in Arratia & Schultze [65] (fig 26b) is of KU 13800 (CS), identified therein as *B*. *brevirostris*. We examined both this cleared and stained specimen and ethanol-preserved specimens from the same lot, and identified them as *B*. *beebei*. We noted that in some specimens the disk-like ossification of the palatoquadrate cartilage is present, but relatively hard to discern due to poor uptake of stain. This may explain why Sullivan [24] did not observe this ossification in some species of *Brachyhypopomus* (*B*. *brevirostris*, *B*. *bullocki*, *B*. *bombilla*, *B*. sp. “regani”, *B*. sp. “sullivani”).

#### Intramuscular bones

58. *Form of post-Weberian dorsal myorhabdoi*. (0) post-Weberian dorsal myorhabdoi unbranched; (1) post-Weberian dorsal myorhabdoi with branched structure (CI = 0.50; RI = 0.50).

The post-Weberian myorhabdoi are simple, unbranched filament-like bones (state 0) in all species of *Brachyhypopomus* except *B*. sp. “flavipomus”, and are also simple and unbranched (state 0) in *Gymnorhamphichthys*, *Gymnotus*, *Hypopomus*, *Microsternarchus*, *Racenisia*, *Rhamphichthys*, and *Sternopygus*. The post-Weberian dorsal myorhabdoi exhibit a branched structure (state 1; Lundberg & Mago-Leccia [55]: 59, fig 6) in *Brachyhypopomus* sp. “flavipomus”, and in *Hypopygus* and *Steatogenys*. This character is not applicable to *Racenisia*, in which the post-Weberian dorsal myorhabdoi are absent.

#### Sexual dimorphism of pigmentation

59. Occurrence of sexual dimorphism in pigmentation. (0) absent; (1) females possess distinctly darker pigmentation than males (CI = 1.00; RI = 1.00).

Sexual dimorphism of pigmentation (state 0) is absent in all outgroups, and in all species of *Brachyhypopomus* except *B*. sp. “alberti”, and *B*. sp. “arrayae”. Conversely, females exhibit distinctly darker pigmentation than males (state 1) in *B*. sp. “alberti”, and *B*. sp. “arrayae”.

#### Accessory electric organ

60. *Occurrence of accessory electric organ on opercular region*. (0) absent; (1) present (CI = 1.00; RI = 1.00).

Among the Rhamphichthyoidea accessory electric organs (AEOs) are known in *Steatogenys* species, which possess a paired mental and humeral AEO [66, 67], and *Hypopygus*, which possess a paired post-pectoral AEO [49]. A paired AEO overlying the operculum and lying immediately under the skin was noted by Sullivan [24] and Carvalho [22] in the genus. The paired opercular AEO is absent (state 0) in all *Brachyhypopomus* species except *B*. *bombilla*, *B*. sp. “menezesi”, and *B*. sp. “regani”, and is also absent (state 0) in all outgroups. Conversely, a paired opercular AEO is present (state 1; Sullivan [24]: 322, fig 54; Carvalho [22]: 177, fig 37) in *Brachyhypopomus*: *B*. *bombilla*, *B*. sp. “menezesi”, and *B*. sp. “regani”. In all three of these species, the AEO is an inverted U-shaped structure which originates near the anus and extends to approximately half-way up the head. It widens from a stalk-like ventral portion to a wider distal portion. The opercular AEO in all three species appears to represents a continuation of the electrocytes and associated gel-like matrix of the hypaxial organ, which extends anterior to the anal and urogenital pores and divides near the isthmus into the paired AEO. The AEO is a peduncular structure comprising translucent oblong or polygon-shaped electrocytes resembling those in the main hypaxial organ. The electrocytes are arranged irregularly in approximately three vertically oriented series; each series comprising some 6–10 electrocytes. The entire AEO is overlain by a thin layer of translucent skin. In *B*. sp. “menezesi” and *B*. sp. “regani”, the skin overlying the AEO possesses minimal chromatophores, such that the AEO and its margins are clearly visible as a pale patch. In contrast, the skin overlying the AEO of *B*. *bombilla* often exhibits a higher density of chromatophores, which occlude the outline of the organ. This probably accounts for why Loureiro & Silva [68] failed to mention the AEO in their description of *B*. *bombilla*.

### Geographic Distributions

**Fig 18:**
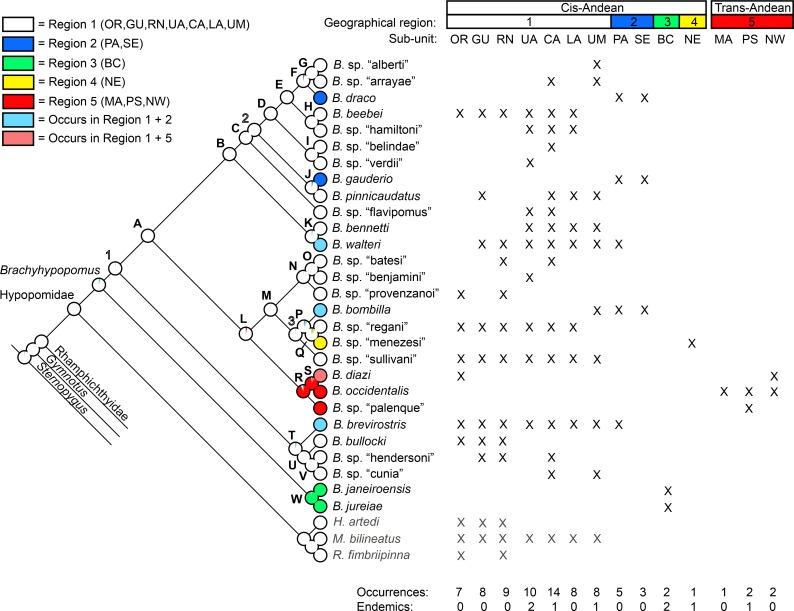
Geographical distributions of *Brachyhypopomus* and other hypopomids. Occurrence records are tabulated for five geographical regions and 14 drainage subunits. Crosses show occurrences for each species. The tree topology follows the total evidence BI phylogeny reported in Fig 7. Letters denote well-supported clades and numbers denote poorly-supported clades based on Bayesian Posterior Probabilities (see text and Fig 5). Colored circles at the terminals represent occurrence in one of the five geographical regions (see inset key), or occurrence in both region 1 and 2 (for 3 species), or in region 1 and 5 (for 1 species). Ancestral character states in the internal nodes are optimized by maximum likelihood, with the proportion of the color representing the probability of occurrence in a given region. The ancestral character state for *Brachyhypopomus* optimizes with high probability to Greater Amazonia. Records for outgroup species are in grey text. The regions listed here are as follows (for details of drainage boundaries see ‘Geographic and ecological distributions‘ in Materials and Methods): **Region 1 (Greater Amazonia):** (OR) = Orinoco basin; (GU) = Caribbean drainages of the Guianas; (RN) = rio Negro; (UA) = Upper Amazon; (CA) = Central Amazon; (LA) = Lower Amazon; (UM) = Upper Madeira. **Region 2 (La Plata–Lagoa dos Patos):** (PA) = La Plata drainages; (SE) = Lagoa dos Patos-Mirim system and adjacent coastal drainages of Brazil and Uruguay. **Region 3 (Brazilian coastal drainages):** (BC) = Atlantic coastal drainages from rio Ribeira de Iguape to rio Paraíba do Sul. **Region 4 (São Francisco drainage):** (NE) = rio São Francisco. **Region 5 = (Trans-Andean drainages):** (MA) = Middle America; (PS) = Pacific Slope; (NW) = northwest South America.

The occurrences of 28 species of *Brachyhypopomus* and three other hypopomid genera among the 14 drainage units described in ‘Geographic and ecological distributions’ (see Materials and Methods) are tabulated in Fig 18. This figure integrates georeferenced museum collection records for 11,750 specimens of *Brachyhypopomus* from 2,642 museum lots, presented in Crampton et al. [3] (fig 2). The 14 drainage units listed in Fig 18 are grouped into five geographic regions, which we optimize as character states on the Bayesian total evidence tree summarized in Fig 7 in order to explore biogeographical distributions in the phylogenetic context (see ‘General patterns of diversification’ in the Discussion). This approach mirrors that adopted by studies of the biogeography of two other widely distributed gymnotiform taxa: *Gymnotus* [53, 69], and *Sternopygus* [61]. The first four regions correspond to cis-Andean drainages (those to the east and south of the Andes). Region 5 groups trans-Andean basins (those to the west and north of the Andes).

### Ecological Distributions

*Brachyhypopomus* species are restricted to shallow-water ecosystems. These divide into two categories: floodplain systems subject to a predictable seasonal inundation cycle, and terra firme systems lying above the extent of seasonal flooding. In floodplain habitats, *Brachyhypopomus* live and breed in the root mats of floating rafts of grasses and other macrophytes. In terra firme systems *Brachyhypopomus* usually occur in and around aquatic plants, marginal root mats, and submerged leaf litter, debris, or in the case of shield and piedmont streams, rocks and stones. *Brachyhypopomus*, and other hypopomids, sensu Maldonado-Ocampo [14], are conspicuously absent from the benthos of large, deep, river channels, where Apteronotidae, Sternopygidae, and some Rhamphichthyidae are abundant [70].

Depending primarily upon catchment geology and geomorphology, Neotropical freshwaters exhibit substantial variation in water chemistry, including variation in conductivity and dissolved oxygen–parameters that are known to influence the localized distribution of *Brachyhypopomus*, and other electric fish [2, 5, 8, 71, 72].

As summarized in Crampton [22], Neotropical floodplain systems are typically of Quaternary origin and flank major rivers along their entire lowland courses–forming mosaics of lakes, channels, and seasonally flooded forest or grassland. Floodplains divide broadly into three major categories. 1. *Whitewater floodplains*: these flank high conductivity (ca 60–300 μScm^-1^) sediment-rich rivers of Andean origin (e.g. the rio Paraguay, Apuré, Marañon, Ucayali, Juruá, and Madeira). 2. *Blackwater floodplains*: these flank low conductivity (ca 5–30 μScm^-1^), sediment-poor humic-stained blackwater rivers that derive from forested lowland Paleogene-Neogene formations (e.g. the rio Japurá, Tefé, Negro, Uatamã, and Arapiuns). 3. *Clearwater floodplains*: these flank low conductivity, sediment-poor (ca 5–30 μScm^-1^) clearwater rivers of shield origin (e.g. the rio Tapajós, Xingú, and Tocantins). Whitewater floodplain waters become anoxic or severely hypoxic during the flood period as a consequence of the decomposition of accumulated leaf litter and other organic debris in inundated forests. In contrast, blackwater and clearwater floodplains waters usually remain well oxygenated through the year (> 2.0 mg/l), primarily due to the small extent of their inundated forests in comparison to whitewater floodplains (and consequently lower rates of deoxygenation from decomposing organic debris) [2, 72].

Terra firme systems occur in a greater diversity of geological formations, and divide into four categories [22]–the first three of which are permanently normoxic (dissolved oxygen >2.0 mgl^-1^). 1. *Lowland terra firme streams*: low-conductivity (ca. 5–30 μScm^-1^), sediment-poor, blackwater or clearwater streams and small rivers draining lowland (< ca. 200 m above sea level) Paleogene or Neogene formations; these formations including the giant forest and savanna-covered peneplain of lowland Amazonia. 2. *Upland shield streams*: low-conductivity (ca. 5–30 μScm^-1^), sediment-poor, clearwater upland streams (> ca. 200 m) draining Proterozoic or Paleozoic formations of the Guiana and Brazilian Shields. 3. *Upland piedmont streams*: high conductivity (ca. 100–500 μScm^-1^), sediment-rich (whitewater) upland streams (> ca. 200 m) draining the erosion zones of Andean or Panamanian piedmont formations, which are of Mesozoic or Paleogene origin [22]. 4. *Lowland terra firme swamps*: shallow (typically < 0.5 m), low-conductivity (ca. 5–30 μScm^-1^), sediment-poor blackwater or clearwater ephemeral or permanent swamps that form in depressions or poorly drained valleys in lowland Paleogene or Neogene formations. These become intermittently hypoxic (< 0.5 mgl^-1^) due to the accumulation of leaf litter, and are colonized by fishes from adjacent terra firme streams (the first category, above) following local flash floods [73].

**Fig 19:**
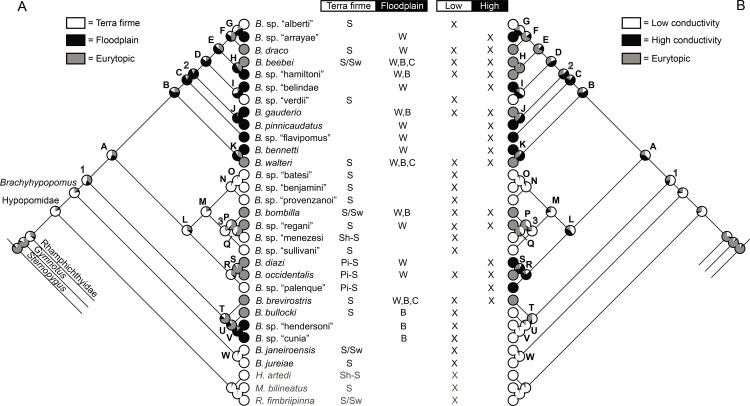
Ecological distributions of *Brachyhypopomus* and other hypopomids. (A) Habitat occupancy in terra firme and floodplain systems (letter codes listed below denote occurrences). (B) Occupancy of low (< 30 μScm^-1^) and high conductivity (> 60μScm^-1^) systems (crosses denote occurrences). The tree topology follows the total evidence BI phylogeny reported in Fig 7. Letters on the trees denote well-supported clades and numbers denote poorly-supported clades based on Bayesian Posterior Probabilities (see text and Fig 5). Records for outgroup species are in grey text. The circles of the terminals represent exclusive occurrence in either of two habitats (white/black), or eurytopy (grey). Ancestral character states in the internal nodes are optimized by maximum likelihood, with the proportion of white, black, and grey representing the probability of occurrence in a given habitat/conductivity. Letter codes for habitat occupancy: Terra firme systems: S = lowland (< ca. 200 m above sea level) terra firme stream in Paleogene-Neogene formations, Sw = lowland terra firme swamp in Paleogene-Neogene formations; Sh-S = upland (> ca. 200 m above sea level) stream in Proterozoic-Paleozoic shield formations; Pi-S = upland (> ca. 200 m above sea level) stream in Mesozoic-Paleogene Andean or Panamanian piedmont formations. Floodplain systems: W = whitewater, B = blackwater, C = clearwater.

The distributions of *Brachyhypopomus* (and three other hypopomid species) among the floodplain and firme systems described above are summarized in Fig 19A. Nine species of *Brachyhypopomus* are eurytopic–occupying both floodplain and terra firme systems. Nine are specialized to river floodplain systems. Ten species are endemic to terra firme systems. We explore these habitat distributions in the phylogenetic context in Fig 19A. Here, on the total evidence phylogeny, we optimize exclusive occurrence in floodplains, exclusive occurrence in terra firme streams, and eurytopy as three character states.

The occurrences of *Brachyhypopomus* species (and three other hypopomid species) in low conductivity systems (ca. 5–30 μScm^-1^) and high conductivity systems (ca. 60–500 μScm^-1^) are presented in Fig 19B. Nine species of *Brachyhypopomus* are eurytopic with regard to conductivity. Twelve are endemic to low conductivity systems, and seven are endemic to high conductivity systems. In Fig 19B we also explore distributions with regard to conductivity in the phylogenetic context.

**Fig 20:**
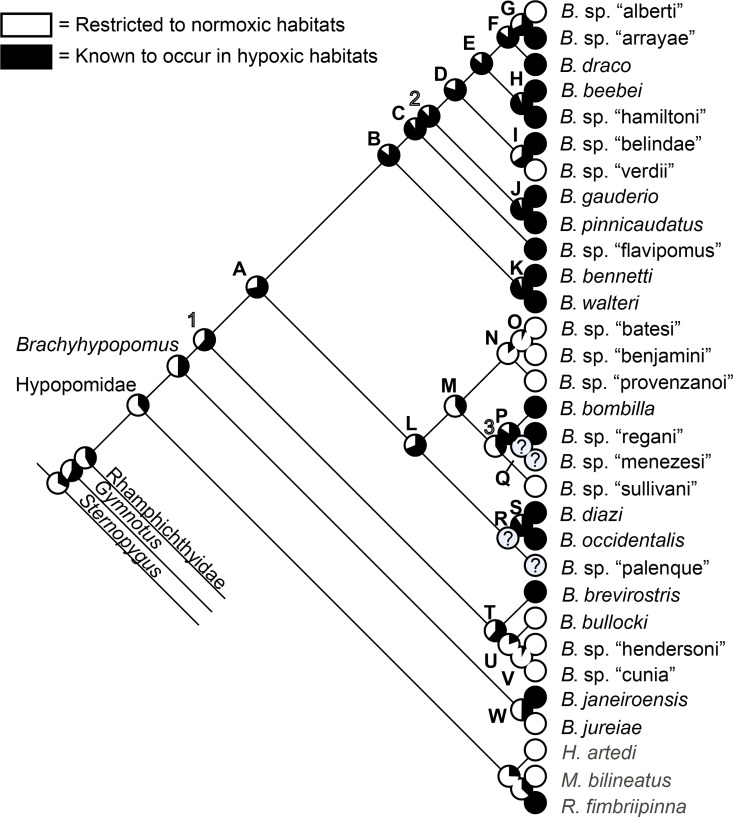
The occurrence of *Brachyhypopomus* and other hypopomids in habitats subject to hypoxia. The tree topology follows the total evidence phylogeny reported in Fig 7. Letters denote well-supported clades and numbers denote poorly-supported clades based on Bayesian Posterior Probabilities (see text and Fig 5). Records for outgroup species are in grey text. Ancestral character states in the internal nodes are optimized by maximum likelihood, with the proportion of white and black representing the probability of occurrence in a given habitat/conductivity environment.? = unknown state in terminal or equivocal ancestral condition.

In Fig 20 we classify *Brachyhypopomus* species (and three other hypopomid species) as either “known to occur in habitats subject to intermittent or perennial hypoxia” (< 0.5 mgl^-1^) (sixteen species of *Brachyhypopomus*) or “restricted to normoxic habitats” (always > 0.5 mgl^-1^) (ten species of *Brachyhypopomus*). Here we also optimize these character states onto the total evidence phylogeny for hypopomids.

## Discussion

### The Monophyly of *Brachyhypopomus*

Bayesian analysis of the cytb and rag2 genes separately (Figs 2 and 3), both genes combined (Fig 4), and both genes combined with morphological data (total evidence) (Figs 5 and 7) all provide strong support for a monophyletic *Brachyhypopomus* (nodal support by Bayesian Posterior Probabilities in each case = 1). Parsimony total evidence phylogenetic reconstruction (Fig 6) also provides support for a monophyletic *Brachyhypopomus* (nodal bootstrap support = 98%, Bremer support = 12).

In contrast, our phylogenetic analysis based on morphological characters alone reconstructed a paraphyletic *Brachyhypopomus*, with Microsternarchini at a deeply nested position in the genus, as part of a clade also including *B*. sp. “batesi”, *B*. sp. “benjamini”, and *B*. sp. “provenzanoi” (sp. “batesi” species-group) (Fig 1). We suspect that the placement of the Microsternarchini in our morphology-based phylogeny results from the sharing of homoplastic characters associated with stream-dwelling and diminutive size in the Microsternarchini and sp. “batesi”-group (see ‘Phylogenetic interrelationships within Brachyhypopomus‘, below), and is therefore incorrect.

Only one morphological character qualifies as an unambiguous, unreversed morphological synapomorphy for *Brachyhypopomus*, and is therefore of diagnostic value: the derived presence of a disk-like ossification in the anterior portion of the palatoquadrate cartilage in adult specimens (character 57; Fig 17). Lack of ossification of the palatoquadrate is the ubiquitous condition in all rhamphichthyoid outgroups included in our analyses. Although we did not include the other known rhamphichthyoid genera *Iracema*, and *Procerusternarchus* in our dataset, it appears that these taxa also lack ossification of the palatoquadrate. X-ray computed tomography images of the suspensorium of *Iracema caiana* and *Procerusternarchus pixuna* show no indication of ossification of the palatoquadrate cartilage (Carvalho & Albert [74]: 462, fig 5; Fernandes et al. [18]: 101, fig 3). Photographs of cleared and stained specimens of *Akawaio penak* (ROM 83884) show the palatoquadrate cartilage is also unossified in this genus. Our conclusion regarding the monophyly of *Brachyhypopomus* is contingent on the taxa we included in this analysis; collection of full morphological and molecular character data for *Iracema*, *Akawaio*, and *Procerusternarchus* will be necessary for a comprehensive test of *Brachyhypopomus* monophyly.

Based on the Bayesian total evidence topology (Figs 5 and 7), morphological characters 22, 32, 41, 46, and 50 are also optimized as synapomorphies for *Brachyhypopomus*, but have limited diagnostic value. In some cases these characters are reversed in the ingroup and/or outgroup taxa, in some taxa the characters are inapplicable, and in some cases complete character state data are not yet available for all taxa. We speculate that two of these synapomorphies could represent additional diagnostic characters for *Brachyhypopomus*: the presence of premaxillary teeth at some stage in ontogeny (Character 46), and the presence of dentary teeth at some stage in ontogeny (Characters 50).

*Premaxillary teeth as a potential synapomorphy*: We noted the presence of teeth on the premaxilla of small juvenile specimens (< 50 mm TL) of all three species of *Brachyhypopomus* for which specimens of this size were available (*B*. *brevirostris*, 47.7 mm; *B*. *diazi*, 20 mm; *B*. sp. “palenque”, 31 mm), and also in both immature (<75 mm TL) and mature adult (>140 mm TL) specimens of two additional species: *B*. *bennetti* and *B*. *walteri*. These observations suggest that the presence of premaxillary teeth early in ontogeny may be ubiquitous in the genus. Premaxillary teeth have never been reported for other rhamphichthyoid genera [20, 50, 51], and we confirmed the absence of teeth on the premaxilla not only in adults of all examined rhamphichthyoid genera outside *Brachyhypopomus* (i.e. *Hypopomus*, *Hypopygus*, *Microsternarchus*, *Racenisia*, *Rhamphichthys*, and *Steatogenys*) but also in small juvenile specimens (< 45 mm) for *Hypopygus*, *Microsternarchus*, *Racenisia*, and *Rhamphichthys*. We suspect that a more thorough survey of the osteology of juvenile rhamphichthyoid may reveal that the presence of premaxillary teeth at some stage in ontogeny is a diagnostic character for *Brachyhypopomus*.

*Dentary teeth as a potential synapomorphy*: Mago-Leccia [51], Albert & Campos-da-Paz [50], and Albert [20] stated that members of Rhamphichthyoidea are recognized by the absence of teeth on the dentary. However, as with premaxillary teeth, we noted the presence of teeth on the dentary of small juvenile specimens (< 50 mm) of all of three species of *Brachyhypopomus* for which specimens of this size were available (*B*. *brevirostris*, 47.7 mm; *B*. *diazi*, 20 mm; *B*. sp. “palenque”, 31 mm), and also in a larger juvenile specimen of *B*. *walteri* (73 mm). In contrast, we confirmed the absence of dentary teeth in both adults and small juveniles (< 42 mm) of *Hypopygus*, *Microsternarchus*, *Rhamphichthys*, and *Racenisia* (note: we were unable to confirm Fernandez-Yépez’s [64] report of the presence of dentary teeth in *Microsternarchus bilineatus*). Based on these observations we propose that the presence of dentary teeth at some stage in ontogeny is a diagnostic character for *Brachyhypopomus*–although further work is required to confirm this.

### Phylogenetic interrelationships within *Brachyhypopomus*

Below, we discuss phylogenetic relationships among *Brachyhypopomus* species. We emphasize the results for the total evidence Bayesian phylogenetic analysis, but provide comparisons with the parsimony analysis, and individual gene tree analyses when relevant.

#### Major clades and species groups

Bayesian total evidence phylogenetic reconstruction (Figs 5 and 7) provides strong support for four major clades within the genus: 1) Clade B comprising the *beebei*, sp. “belindae”, *pinnicaudatus*, and *bennetti* species-groups, and *B*. sp. “flavipomus”; 2) Clade L, comprising the sp. “batesi”, *bombilla*, and *occidentalis* species-groups, and *B*. sp. “sullivani”; 3) Clade T–the *brevirostris* species-group; and 4) Clade W–the *janeiroensis* species-group. At a higher level, there is strong support for a clade A comprising clades B and L. The internal nodes within the four major clades B, L, T, and W are strongly supported, with only two exceptions: First, a poorly supported Clade 2 comprising the *pinnicaudatus* species-group and a Clade D comprising the *beebei* and sp. “belindae” species-groups. Second, a poorly supported clade 3 comprising the *bombilla* species-group and *B*. sp. “sullivani”. Parsimony total evidence (Fig 6) recovers the same species-groups resolved by Bayesian analysis (Fig 5), and with the same species composition (with the exception of *B*. sp. “palenque” which appears outside the *B*. *occidentalis* species-group in the parsimony tree but is placed within it in the Bayesian tree).

Bayesian total evidence analysis provides weaker support for some of the higher level clades within the genus. For example, poor support for the monophyly of Clade 1 casts uncertainty on the interrelationships between clades A and T, and on the placement of the *janeiroensis* species-group as sister taxon to the remaining congeners. This instability is reflected by an alternative placement of the *janeiroensis* species-group in the parsimony topology–as sister taxon to the brevirostris species-group. These higher-level interrelationships will be revised in future analyses that incorporate additional molecular data.

#### Species monophyly

The topology and high nodal support values reported in the total evidence analysis support the monophyly of all 28 species of *Brachyhypopomus*, including those with large geographical ranges spanning multiple basins (Fig 18), for which we obtained molecular data from distant sites, e.g. *B*. *bombilla*, *B*. *brevirostris*, *B*. *gauderio*, *B*. *pinnicaudatus*, *B*. *occidentalis*, and *B*. *walteri*. Parsimony analysis resolved *B*. *beebei* as a paraphyletic species containing *B*. sp. “hamiltoni” (Fig 6). However, BI analysis provides strong support (PP = 1) for a monophyletic *B*. *beebei* as the sister species of *B*. sp. “hamiltoni” (Fig 5).

#### Comparison between phylogenies based on morphology and total evidence

The morphological tree presented in Fig 1 shows several areas of precise congruence with the Bayesian total evidence phylogeny: these include strong support for the *bennetti*, *pinnicaudatus*, *bombilla*, *occidentalis*, and *brevirostris* species-groups, and for the Microsternarchini (*Microsternarchus bilineatus* + *Racenisia fimbriipinna*)–all represented by precisely the same species. However, there are several areas of incongruence between the morphological and total evidence trees. First, the topologies of early branching in the genus differ, with nodal support for the higher level clades being considerably weaker in the morphology-based tree. Second, the morphological analysis places the Microsternarchini at a deeply nested position within a non-monophyletic *Brachyhypopomus*–inside a clade comprising other diminutive, slender-bodied stream-dwelling species from the sp. “batesi” species-group. Third, the morphological analysis does not reconstruct a monophyletic *janeiroensis* species-group. Finally, the interrelationship of species within Clade B differs from the total evidence analysis, with the exception of the *pinnicaudatus* and *bennetti* species-groups.

The nested position of Microsternarchini within the sp. “batesi”-group in the morphology-based phylogeny may be explained as follows. The clade comprising *B*. sp. “batesi” + *B*. sp. “provenzanoi” + *B*. sp. “benjamini” + Microsternarchini exhibits the following unambiguous synapomorphies based on parsimony analysis of morphological characters only: derived loss of first branchiostegal ray (character 19); descending process of maxilla a derived narrow shape, versus broad (character 47). The clade *B*. sp. “provenzanoi” + *B*. sp. “benjamini” + Microsternarchini is supported by three additional unambiguous synapomorphies: reversal to ancestral funnel-shape of second basibranchial (character 28); derived loss of gill rakers (character 31); derived loss of scales on dorsal region of anterior third of body (character 45). Further, a clade comprising *B*. sp. “benjamini” + Microsternarchini is supported by a single unambiguous synapomorphy: derived lack of ossification of the third basibranchial (character 29). A pattern emerges–the synapomorphies supporting the grouping of Microsternarchini with *B*. sp. “batesi”, *B*. sp. “benjamini” and *B*. sp. “provenzanoi” (all of which are known to mature at a small body size, less than 80 mm total length, and are confined to small lowland terra firme streams, see Fig 19A) mostly involve derived simplifications of the skeleton and squamation, which we assume to be associated with body size reduction and life in the small interstices of terra firme streams. De Santana & Crampton [49] noted a similar pattern of reductive morphological evolution associated with small body size in *Hypopygus*, which is also restricted to lowland terra firme streams. Our Bayesian and parsimony total evidence analyses provides strong support for a placing of the Microsternarchini outside *Brachyhypopomus*. We therefore speculate that the morphological characters uniting the Microsternarchini and the sp. “batesi” species-group of *Brachyhypopomus* may be homoplastic characters that evolved as convergent responses to similar ecological conditions.

We noted low levels of nodal support in the morphological tree not just for some of the early branching clades (as also observed in the total evidence analysis), but generalized across the phylogeny (except for some very well-supported clades such as the *bennetti*-group, the sister species *B*. sp. “cunia” + *B*. sp. “hendersoni”, and the Microsternarchini). Moreover, we were only able to identify 56 parsimony-informative morphological characters, despite the species richness of the genus, and these came mostly from the cephalic region. This apparent paucity of morphological character variation, coupled with a generalized weakness of nodal support may reflect a generalized pattern of morphological trait conservatism within the genus, which we speculate to be the consequence of one or both of two phenomena, described below.

First, morphological trait conservatism is often a consequence of phylogenetic niche conservatism, in which a taxon diversifies within a relatively narrow range of habitats and ecological variables, and consequently with limited adaptive ecomorphological diversification [75]. All species of *Brachyhypopomus* are restricted to tangled substrates in lentic or slow-flowing environments, and have similar diets of aquatic microinvertebrates, despite some adaptations related to water conductivity and dissolved oxygen availability [2, 8].

Second, a pattern of morphological trait conservatism may be an indirect consequence of speciation driven primarily by sexual selection and reproductive character displacement of mate attraction signals carried by the electric organ discharge (EOD). Because the EOD mate attraction signals of gymnotiforms are thought to play a strong role in prezygotic reproductive isolation and speciation [76, 77], and because gymnotiform fishes express variation in their electric mate attraction signals based on aspects of electric organ microanatomy and electrophysiology [77, 78] that are essentially decoupled from gross external morphology, a mechanism exists for speciation and diversification with little attendant ecomorphological evolution. Arnegard et al. [79] advance similar discussions for the mormyrid electric fish genus *Paramormyrops*.

#### Comparison to previous phylogenetic studies

Our total evidence analyses closely match those of Sullivan's [24] phylogenetic analyses of a smaller subset of *Brachyhypopomus* (11 species), which is summarized in part in Sullivan et al. [63]. First, Sullivan's [24] total evidence analyses, like ours, provides full support for the monophyly of *Brachyhypopomus*. Second, the "*brevirostris*-group" of Sullivan et al. [63] (clade B in Sullivan [24]), comprising *B*. *brevirostris* and *B*. *bullocki*, corresponds to our *brevirostris* species-group). Third, the subgenus *Odontohypopomus* of Sullivan et al. [63] (clade D in Sullivan [24]), comprising *B*. *bennetti* and *B*. *walteri*, corresponds to our *bennetti* species-group. Fourth, the "*beebei*-group" of Sullivan et al. [63] (Clade E in Sullivan [24]), comprising *B*. *beebei*, *B*. *draco*, *B*. *gauderio*, and *B*. *pinnicaudatus* corresponds to our Clade C (comprising our *pinnicaudatus*-, sp. “belindae”-, and *beebei*-groups). Fifth, the "occidentalis-group" of Sullivan et al. [63] (Clade F in Sullivan [24]), comprising *B*. *occidentalis* and *B*. *diazi* corresponds to our *occidentalis* species-group). Finally, Sullivan et al. [63] speculated, based mainly on external appearance, that *B*. *janeiroensis* and *B*. *jureiae* are closely related. This speculation matches our total evidence analyses, which place *B*. *janeiroensis* and *B*. *jureiae* as sister species in the *janeiroensis*-group.

The results of our total evidence analyses are also broadly congruent with Carvalho’s [22] phylogenetic analysis of a combined morphological and molecular dataset that included 14 species of *Brachyhypopomus*. Carvalho’s tree supports our clades B, C, E, L, and our *beebei*, *pinnicaudatus*, and *occidentalis* species-groups. However, it differs in its basal branching patterns, and in the placing of members of the *brevirostris* and *janeiroensis* groups.

Finally, the results of our total evidence analyses correspond partially to a topology for nine species of *Brachyhypopomus* presented in a phylogeny of all gymnotiforms by Tagliacollo et al [23]. This topology recovered our *occidentalis* species-group. It also includes a clade comprising the *occidentalis* species-species + *B*. sp. “sullivani”, listed as *B*. new sp. ‘roy’, (these belong to our clade L), and a clade they resolve comprising *B*. *beebei*, *B*. *draco*, and *B*. *pinnicaudatus* (which belong to our Clade C). Their topology differed in its higher level branching and with regard to the placing of *B*. *brevirostris and B*. *bullocki*.

#### A comment on subgenera in Brachyhypopomus

Sullivan et al. [63] elected to place *B*. *bennetti* and *B*. *walteri* within a sub-genus (*Odontohypopomus*). We do not advocate the use of subgenera to classify clades within *Brachyhypopomus* and instead recommend the adaptable and taxonomically less burdensome species-group approach that we utilize herein, and that has been applied to other species-rich gymnotiform taxa, e.g. *Apteronotus* [80] and *Gymnotus* [77]. Our primary justification for this approach that if a subgeneric name is maintained for *B*. *bennetti* and *B*. *walteri*, then based on our analyses, at least seven other subgenera might need to be created–corresponding approximately to the species groups annotated in Fig 7.

### Generic Interrelationships in the Rhamphichthyoidea

Although our analyses are designed to focus on species interrelationships within *Brachyhypopomus*, we found strong support for a clade comprising the hypopomid genera *Brachyhypopomus*, *Hypopomus*, and Microsternarchini. Likewise, our analyses provide strong support for a positioning of the Steatogeni (*Hypopygus* + *Steatogenys*) as sister taxon to a clade comprising *Gymnorhamphichthys* + *Rhamphichthys*. This mirrors the results of Alves-Gomes et al. [21], Arnegard et al. [81], Chen et al. [82], Carvalho [22], and Maldonado-Ocampo et al. [14] and supports the redefinition of the Hypopomidae by Maldonado-Ocampo et al. [14] to comprise *Akawaio* + *Brachyhypopomus* + *Hypopomus* + *Microsternarchini* and of the Rhamphichthyidae to comprise *Gymnorhamphichthys* + *Iracema* + *Rhamphichthys* + Steatogeni.

### Geographical Distributions and Diversification

To model the geographical distributions of *Brachyhypopomus* in a phylogenetic context, we optimized the distribution of 28 species and three hypopomid outgroup taxa among the five geographical regions described earlier (Results, ‘Geographic distributions’) (Fig 18). Our analyses unequivocally support an origin of *Brachyhypopomus* in Greater Amazonia (Region 1)—the superbasin comprising the rio Amazonas and río Orinoco basins, and the coastal drainages of the Guianas, sensu Albert & Reis [83]. All hypopomid genera other than *Brachyhypopomus* (including *Akawaio penak* and *Procerusternarchus pixuna*; not included in Fig 18) are entirely restricted to this region, mirroring patterns observed in many other Neotropical fish genera [84], the highest species diversity of *Brachyhypopomus* occurs in Greater Amazonia, with 21 of 28 *Brachyhypopomus* species (75%) known from this region (17 exclusively so). From a Greater Amazonian center of origin, *Brachyhypopomus* has evidently subsequently occupied adjacent systems by a combination of vicariance and dispersal; presumably dispersal across permeable watersheds and geodispersal via river capture events, sensu Albert & Crampton [85].

Like many Neotropical fish taxa of similar taxonomic rank [84], the genus *Brachyhypopomus* is evidently of considerable antiquity, indicating processes of cross-basin dispersal, speciation, and extinction that have occurred over periods of geological time that in many cases greatly exceed the age of modern drainage boundaries [22]. Chen et al. [82] estimate the Hypopomidae to have diverged from the Rhamphichthyidae (based on the branching of *Brachyhypopomus* from *Steatogenys* + *Rhamphichthys*) ca. 38 Mya (with 95% confidence intervals ranging from the Paleocene to early Miocene). Lavoué et al. [86] dated this event (based on the divergence of *Brachyhypopomus* from *Gymnorhamphichthys*) to between the Mid and Late Cretaceous, ca. 100–66 Mya (depending upon fossil calibration protocol), with 95% confidence intervals ranging from the Early Cretaceous to Mid Eocene. Near et al. [87] estimate the divergence of Rhamphichthyoidea from Sternopygidae, an event that must substantially precede the origins of *Brachyhypopomus*, at ca. 50 Mya.

Below we discuss salient phylogenetic patterns of distribution for each of the five geographical regions in Fig 18. An emergent pattern is that regional assemblages of *Brachyhypopomus* are polyphyletic in structure, with no evidence of extensive *in-situ* diversification, including within Greater Amazonia. This pattern, which is perhaps ubiquitous in the continental fish fauna of South America, implies a history of dispersal-assembly–sensu Hubbell [88]–from wider, continental-scale species pools, and over long periods of time [2, 85].

We acknowledge that our interpretation of the historical biogeography of *Brachyhypopomus* is limited by the weak support for clade 1 in our total evidence analyses (Fig 5). Moreover, because of the antiquity of the genus, distributional patterns have likely been drastically rearranged on multiple occasions, consequently erasing evidence for early vicariance and dispersal. Finally, we recognize that our interpretations are also to some extent contingent on the accuracy of our recognition of widely distributed species with known population-level genetic sub-structuring (e.g. *B*. *brevirostris*), versus closely related species with allopatric distributions (e.g. *B*. *gauderio* and *B*. *pinnicaudatus*).

#### Region 1. Greater Amazonia

*Brachyhypopomus* has occupied all major non-shield drainages of Greater Amazonia. In some cases modern drainage divides within Greater Amazonia, or major fall and rapid series correspond approximately to the distributional limits of sister taxa–implying a history of vicariant speciation. Examples include *B*. sp. “provenzanoi” in the Orinoco, versus *B*. sp. “batesi” + *B*. sp. “benjamini” in the rio Negro and Upper Amazon, which may reflect fluvial interconnectance across Amazonian foreland arc prior to a Late Miocene reconfiguration of the Amazon's main tributaries [84, 89]. Another example involves three species (*B*. sp. “alberti”, *B*. sp. “arrayae”, and *B*. sp. “cunia”) that are completely, or almost completely restricted to the Upper Madeira, above the major series of cataracts and rapids in its middle course (see also comments for *B*. *bombilla* in the discussion of Region 2, below). *B*. sp. “alberti” + *B*. sp. “arrayae”, and their sister taxon *B*. *draco* (confined to Region 2, the La Plata system and Lagoa dos Patos-Merim and associated drainages of SE Brazil) form a sister taxon to *B*. *beebei* + *B*. sp. “hamiltoni”, a clade that is absent from the Upper Madeira but is distributed over much of the remaining portions of Greater Amazonia. Likewise, *B*. sp. “cunia” is found mostly above the middle-Madeira falls (notwithstanding its description from a site just below the first falls), and is sister taxon to *B*. *bullocki* + *B*. sp. “hendersoni”, a clade restricted to other parts of Greater Amazonia. Several authors have remarked on the high levels of fish endemicity in the Upper Madeira, and attributed this, in part, to isolation from the lowland Central and Lower Amazon by the middle-Madeira falls–the long series of cataracts and rapids (and the absence of broad riverine floodplains) between Porto Velho and Guajará-Mirim [90–93]. Other major Amazonian rivers are interrupted by major falls or rapids in their lower or middle courses (e.g. the Xingú, Tapajós, Tocantins, and Negro). We are unaware, as yet, of endemic *Brachyhypopomus* confined to the headwaters of these systems, but these regions are exceptionally poorly collected.

In other cases, species exhibit distributions that span modern drainage divides within Greater Amazonia, or major waterfalls, suggesting a recent formation of the barriers or relatively recent cross-watershed dispersal. For example, the rio Amazon-río Orinoco divide is bridged by *B*. *bullocki*, *B*. *beebei*, *B*. *brevirostris*, and *B*. sp. “sullivani”–at least judging from their distributions in both upper and lower Orinoco, as well as the upper and lower Negro. The role of chemical gradients along the río Casiquiare (which connects the Orinoco and Negro) and flanking rapids in the upper rio Negro and río Orinoco as filters for dispersal are discussed by Winemiller et al. [94] and Winemiller & Willis [95] (and see ‘Ecological distributions in a phylogenetic context’, below). Other examples of distributions spanning major divides within the Amazon include species that occur both in the rio Negro, and in the Essequibo River (*B*. *bullocki*, *B*. *beebei*, *B*. *brevirostris*, *B*. sp. “hendersoni”, *B*. sp. “regani”, and *B*. *walteri*). The Negro and Essequibo exhibit a contemporary connection via the seasonal wetlands of the Rupununi Savanna, at the headwaters of the Essequibo River and rio Branco (a major rio Negro tributary) [96–98].

A putative example of dispersal between drainages of Greater Amazonia that are currently completely disconnected involves species that known from the Eastern Amazon and also from small drainages along the Atlantic coastal plain of the Guianas—particularly French Guiana and Suriname. These include *B*. *beebei*, *B*. *brevirostris*, *B*. *pinnicaudatus*, and *B*. sp. “regani”. One of these species, *B*. *pinnicaudatus*, exhibits a wide distribution through the Amazon basin, but is absent from the Essequibo (another potential conduit from the Central Amazon via the rio Branco), and thus probably dispersed from the Amazon's modern estuary. Jegú & Keith [99] noted the strong similarity between the freshwater fish population of coastal French Guiana and the main stem of the Amazon River basin, and suggested that the similarity derives from dispersal from the Amazon via its freshwater plume, or by stream capture or interdigitation along the coastal floodplain; see also Lujan & Armbruster [98].

#### Region 2. La Plata—Lagoa dos Patos

Our total evidence phylogeny suggests that two species, *B*. *draco* and *B*. *gauderio*, originated from independent dispersal events from the Amazon basin into the Paraguay basin, with subsequent allopatric speciation. In both cases dispersal across the Upper Madeira—Paraguay divide is implicated because the sister taxa are abundant in the Upper rio Madeira (*B*. sp. “alberti” + *B*. sp. “arrayae” in the case of *B*. *draco*, and *B*. *pinnicaudatus* in the case of *B*. *gauderio*) but absent from other Amazonian tributaries with headwaters close to Paraguay headwaters (Tapajós, Xingú, and Tocantins-Araguaia drainages).

Hubert & Renno [100], Lovejoy et al. [101], and Carvalho & Albert [93] describe the role of the Amazon-Paraguay Divide as a historically semipermeable barrier–acting not only as a barrier promoting allopatric speciation, but also as a conduit for dispersal, faunal exchanges, distributional range extensions, and secondary contact between previously isolated taxa. The authors also discuss pathways for dispersal via river capture between the Amazon and Paraguay via headwaters of the Mamoré, Guaporé, Tapajós, and Xingú, and comment on the existence of contemporary seasonal wetlands that may permit dispersal across these divides.

Carvalho & Albert [93] note that around one third of the species known from the Paraguay basin also occur in southern Amazon tributaries or other parts of Amazonia, suggesting that a large proportion of species that have crossed the Amazon-Paraguay divide did so recently, and have not yet diverged into diagnosable species; despite a trend for even relatively subtle morphological or genetic variation between populations in species shared between major basins to be assumed to represent species-level divergence [102]. Mirroring this observation for the Paraguay basin ichthyofauna as a whole, three of the five species of *Brachyhypopomus* known from the rio Paraguay, are also known from southern Amazon drainages: *B*. *bombilla*, *B*. *brevirostris*, and *B*. *walteri*, with the topology of our total evidence analyses implying dispersal into the Paraguay from the Amazon in each case. In contrast to *B*. *brevirostris* and *B*. *walteri*, whose distributions are mostly centered on the Amazon and other parts of Greater Amazonia, the range of *B*. *bombilla* is centered primarily on the rio Paraguay-Paraná-Uruguay, and the Lagoa dos Patos system and adjacent coastal drainages. Populations of *B*. *bombilla* from the Upper Madeira are morphologically similar to populations from high southern latitudes, and together these form a monophyletic group in our total evidence analyses. We speculate that *B*. *bombilla* may have been isolated from its sister taxon (clade Q, with a distribution encompassing most of Greater Amazonia and the rio São Francisco) by the middle Madeira falls (see above)–with concomitant or subsequent dispersal across the Guaporé-Paraguay Divide. *Brachyhypopomus brevirostris* and *B*. *walteri* are represented in multiple southern Amazon drainages with headwaters abutting rio Paraguay headwaters. Consequently, pathways for dispersal into the rio Paraguay are unknown for these two species but may be elucidated by future population genetic analyses that incorporate populations from the headwaters of the Paraguay, Guaporé, Tapajós, Xingú, and Upper Tocantins-Araguaia. *Brachyhypopomus* is absent from the entire rio Paraná drainage upstream of the former Guaíra Falls (drowned by the Itaipu hydroelectric dam since 1982), indicating that the colonization of the La Plata drainages by *Brachyhypopomus* is unlikely to have occurred via an Amazon-Paraná conduit.

*The Lagoa dos Patos-Merim drainage*, *and adjacent coastal drainages*: Three species, *B*. *bombilla*, *B*. *draco*, and *B*. *gauderio* exhibit similar southern distributions, which bridge the Paraguay-Paraná-Uruguay system and the Lagoa dos Patos-Mirim drainage (and for *B*. *draco* and *B*. *gauderio* the rio Tramandaí and rio Maquiné –small coastal drainages adjacent to the Lagoa dos Patos). Extensive faunal sharing between these drainages has been noted in other groups of fishes [103, 104].

#### Region 3. Brazilian coastal drainages

The origins of *B*. *jureiae* and *B*. *janeiroensis* (which together form a strongly supported *B*. *janeiroensis* species-group, clade W), require a separate explanation to the three species from the more southerly coastal Brazilian systems of the Lagoa dos Patos-Merim system and adjacent rio Tramandaí and rio Maquiné (see above). *Brachyhypopomus jureiae* and *B*. *janeiroensis* occur further to the north, and occupy small distributional ranges–*B*. *jureiae* from the Ribeira de Iguape [105], and *B*. *janeiroensis* from the São João and rio Paraiba do Sul drainages [106]. These limited ranges are relatively unusual for species from these drainages; many other fish groups in the Ribeira de Iguape and Paraiba do Sul systems are commonly also known from upper rio Paraná drainages to the east of the coastal mountain ranges of eastern Brazil [103, 107]. Nonetheless, species of *Brachyhypopomus* are unknown from the upper Paraná.

Our Bayesian total evidence phylogenetic analysis provides some support for a basal divergence between the *janeiroensis* species-group and all remaining *Brachyhypopomus* species, which belong to Clade 1. Nonetheless, weak support for the monophyly of Clade 1 (Fig 5), and an alternative placement of the *janeiroensis* species-group in our parsimony analysis (as sister taxon to the *brevirostris* species-group, see Fig 6) diminish our confidence in the early branching events in *Brachyhypopomus*. Regardless of its phylogenetic position, we postulate that the *janeiroensis* species-group originated by allopatric isolation across the main block of the Brazilian Shield, following colonization of its south-eastern fringe from a Greater Amazonian center of diversification. Similar sister-clades from the Amazon and southeast coastal drainages (without species occurring in intervening areas) have been described in several other Neotropical fish groups; see "pattern B" vicariant patterns described by Ribeiro [103].

#### Region 4. São Francisco drainage

The rio São Francisco drains a large portion of the central Brazilian Shield but hosts only one species of *Brachyhypopomus*–*B*. sp. “menezesi”. This species is the only member of the genus that is entirely restricted to a shield drainage. The sister-species relationship between *B*. sp. “menezesi” and *B*. sp. “regani”, which occurs over wide areas of Greater Amazonia, suggests a history of dispersal from an Amazon drainage and subsequent allopatric speciation, perhaps from a rio Tocantins tributary (*B*. sp. “regani” is known from the upper Araguaia but not upper Tocantins), or from populations once present in the rio Parnaíba drainage; see Buckup [107] (*Brachyhypopomus* is not known from the Parnaíba).

#### Region 5. Trans-Andean drainages

Northwestern South America and the Isthmus of Panama have experienced greater geological upheaval than any other area of the Neotropics–including the partitioning of the Maracaibo basin, Magdalena basins, Pacific coastal systems, and Caribbean coastal drainages by multiple Andean orogenies, and the Plio-Pleistocene (or earlier) closure of the Panamanian isthmus. These transformations, and their impact on the ichthyofauna, have been reviewed by multiple authors [69, 101, 108–111]. Two species of *Brachyhypopomus*, *B*. *occidentalis* and *B*. sp. “palenque”, are exclusively trans-Andean. One species, *B*. *diazi* occurs in both trans- and cis-Andean drainages.

Our total evidence analyses support a single trans-Andean vicariant speciation event between clade R–the *occidentalis* species-group and clade M (a clade almost entirely restricted to Greater Amazonia, see Fig 18). This was followed by a divergence between B. sp. “palenque”, which occurs in southerly Pacific drainages of Ecuador, and the clade comprising *B*. *occidentalis* + *B*. *diazi*. *Brachyhypopomus occidentalis* is known from multiple Pacific and Atlantic drainages of Colombia, Panama, and Venezuela, including the Maracaibo drainage. *Brachyhypopomus diazi* is the only species of hypopomid that occurs in both cis-Andean drainages (Llanos wetlands of the río Orinoco) and trans-Andean drainages (Caribbean coastal drainages to the north of the Venezuelan Caribbean Cordillera). Our total evidence topology suggests that *B*. *diazi* colonized the Orinoco via range extension from trans-Andean drainages, rather than the reverse–despite occupying a wider geographical distribution in the Orinocan Llanos than in the trans-Andean coastal drainages of the Caribbean coast.

We noted a striking degree of genetic similarity between *B*. *diazi* populations in the trans-Andean coastal río Salado drainage (the type locality), and in the cis-Andean Llanos. For cytb, all four individuals sequenced across these two areas (Table 3) are identical, except for *B*. *diazi*_305_OR, which showed a single base pair difference (0.09% uncorrected sequence divergence) from the other three individuals. For rag2, we found a maximum of two base pair differences (0.2% uncorrected divergence) between *B*. *diazi*_305_OR and *B*. *diazi*_2409_NW. These divergences are consistent with a recent dispersal or translocation event. Dispersal by stream capture across a low-lying area of the Caribbean Cordillera is a possibility, and there are candidate sites for such an event where headwaters of the río Yaracuy (Carribbean drainage) reach within 2–3 km of headwaters of headwaters of the río Portuguesa (Orinoco drainage) at ca. 10°06°N, 068°58'W and at an elevation of around 300 m. Dispersal events from trans-Andean to cis-Andean drainages are not common in Neotropical fish (for a review of cis-trans Andean vicariance see [108]), and *B*. *diazi* may represent one of the first cases for which there is strong support from phylogenetic and genetic data.

### Ecological Distributions

To model ecological diversification and specialization in *Brachyhypopomus* in a phylogenetic context, we considered distributions based on habitat occupancy, electrical conductivity, and dissolved oxygen in light of our total evidence BI phylogeny (Figs 19 and 20).

#### Habitat occupancy

All hypopomid outgroups to *Brachyhypopomus* are restricted to (low-conductivity) terra firme streams and swamps, or shield stream systems (including *Akawaio penak* and *Procerusternarchus pixuna*, not included in our phylogeny). This is reflected by the optimization of habitat and conductivity onto the total evidence phylogeny in Fig 19. Here the ancestral condition for the genus *Brachyhypopomus* is optimized as terra firme (white) in Fig 19A and low conductivity (white) in Fig 19B with much higher certainty than floodplain (black) or high conductivity (black) or grey (eurytopic for habitat or eurytopic for conductivity). The ancestral habitat of *Brachyhypopomus* therefore likely resembled a low-conductivity non-floodplain system.

Floodplain specialization has evidently evolved in at least two clades: clade B (both high- and low-conductivity floodplains), and clade V–comprising *B*. sp. “cunia” and *B*. sp. “hendersoni”) (low-conductivity blackwater floodplains only). Within clade B there are subsequent reversals to stream occupation: in *B*. sp. “alberti” and *B*. sp. “verdii” (stream specialists) and in *B*. *beebei*, *B*. *draco*, and *B*. *walteri* (eurytopic species). Outside clade B there are some transitions from an ancestral character state optimized with high probability as terra-firme stream to a eurytopic condition–notably *B*. *bombilla*, *diazi*, *B*. *occidentalis*, and *B*. sp. “regani”.

#### Conductivity

The ancestral habitat in *Brachyhypopomus* is optimized with high probability as low-conductivity. This is mostly retained in clade T, with a transition to a eurytopic condition in *B*. *brevirostris*. Although the ancestral character states of higher level clades 1, A, and B are ambiguous, a pattern emerges in which species in Clade B are mostly specialized to high conductivity systems, or eurytopic–with reversals to the occupation of low-conductivity systems in *B*. sp. “alberti”, and *B*. sp. “verdii”. Within Clade L, the cis-Andean species in Clade M are mostly specialized to low-conductivity systems, with some derived transitions to eurytopy. In contrast, the trans-Andean species belonging to clade R (the *occidentalis* species-group) optimize as high-conductivity specialists, with a transition to eurytopy in *B*. *occidentalis*.

Occurrence in floodplains (Fig 19A) correlates approximately to occurrence in high-conductivity systems (Fig 19B), and likewise occurrence in terra-firme streams correlates approximately to occurrence in low-conductivity systems. These correlations occur because whitewater floodplains and lowland terra firme streams (in which *Brachyhypopomus* are especially diverse) are characterized by high and low conductivity, respectively. However, the correspondence is imperfect because the Andean and Panamanian piedmont terra firme streams inhabited by *B*. *diazi*, *B*. *occidentalis*, and *B*. sp. “palenque” are characterized by high-conductivity. Likewise, the blackwater floodplain systems inhabited by members of the *brevirostris* species-group are characterized by low conductivity (see ‘Ecological distributions‘, in Results).

Models of impedance matching presented by Hopkins [112] predict correlations between conductivity and the arrangement of electrocytes in the caudal portion of the electric organ, which is located mainly in the caudal filament and generates the high amplitude component of the EOD used in communication [113]. In low conductivity systems, maximum EOD power is associated with a predominantly serial configuration of the electrocytes in a long caudal filament, while in high conductivity systems power is maximized by a parallel configuration of electrocytes in a short caudal filament. As predicted by these models, *Brachyhypopomus* specialists of high conductivity systems have short tails with a parallel arrangement of electrocyte (e.g. *B*. *bennetti*, *B*. *diazi*, *B*. *occidentalis*, *B*. sp. “palenque”), while species specialized to low conductivity systems have relatively long tails (e.g. *B*. *brevirostris*, *B*. *bullocki*, *B*. sp. “cunia”, *B*. sp. “hendersoni”, *B*. *janeiroensis*, *B*. *jureiae*). These characters are also known to be exaggerated in the breeding males of some of these species [5, 63, 71, 112]. The optimization of conductivity on our total evidence tree (Fig 19B) indicates that salient impedance matching adaptations to high conductivity may have evolved in response to a transition from an ancestral low-conductivity system independently in at least two lineages: in Clade B (*B*. *bennetti* and *B*. *beebei*), and in all three species in the *occidentalis* species-group. Nonetheless, some species endemic to high conductivity systems (*B*. sp. “arrayae”, B sp. “flavipomus”), and to low conductivity systems (*B*. sp. “alberti”, *B*. sp. “batesi”, *B*. sp. “benjamini”, *B*. sp. “menezesi”, *B*. sp. “provenzanoi”. *B*. sp. “sullivani”) exhibit no obvious electric organ or caudal filament specializations of the kind associated with impedance matching, and no salient sexual dimorphism.

Impedance matching to a relatively narrow range of conductivity has been predicted to act as a barrier to the dispersal of *Brachyhypopomus* species, given the geographical distribution of low and high-conductivity rivers in the Neotropics [71, 112]. The geographical distributions and habitat occupancies summarized in Figs 18 and 19 provide some support for this prediction. For example, low-conductivity blackwater conditions are found across the Orinoco-Negro divide, and Winemiller & Willis [95] have argued that this allows the dispersal of blackwater adapted fishes or those tolerant of variable water conditions. *Brachyhypopomus* species bridging the Orinoco-Negro Divide (labeled OR and RN in Fig 18) are, as predicted, all either low-conductivity blackwater specialists (*B*. *bullocki* and *B*. sp. “sullivani”) or eurytopic (*B*. *beebei* and *B*. *brevirostris*). Likewise, species shared between the Essequibo (part of the GU region listed in Fig 18) and rio Negro (RN), which are both low-conductivity blackwater systems, are also either low-conductivity blackwater specialists (*B*. *bullocki*, *B*. sp. “hendersoni”), or eurytopic (*B*. sp. “regani”, and *B*. *walteri*). In contrast, species that are specialized to high-conductivity whitewater systems are evidently unable to traverse long corridors of low-conductivity water (often with rapids) to reach similar habitats in adjacent basins (e.g. *B*. *diazi*, restricted to the Orinoco basin, and *B*. *bennetti*, *B*. sp. “belindae”, *B*. sp. “flavipomus”, and *B*. *pinnicaudatus*, restricted to the Amazon basin).

#### Hypoxia

The capacity to occupy habitats with prolonged hypoxia (Fig 20) approximately mirrors the occupation of high-conductivity whitewater floodplains (Fig 19A). All clade B members, except *B*. sp. “alberti” and *B*. sp. “verdii”, occur in hypoxic habitats, as do several species in clade P and R. The occupation of hypoxic habitats has also arisen sporadically in three other species: in *B*. *brevirostris*, and also in *B*. *janeiroensis*, and *Racenisia fimbriipinna* (both of which are known to occur in hypoxic terra firme swamp habitats). The ancestral condition regarding tolerance of hypoxia is ambiguous.

Most species of *Brachyhypopomus* persist in hypoxic water by undertaking aerial gill respiration–either by aspirating bubbles of air into their gill chambers, or by opening their mouths at the surface to expose the gills to air (*B*. *beebei*, *B*. *gauderio*, *B*. sp. “flavipomus”, *B*. *pinnicaudatus*, and *B*. *walteri*) [72, 114, 115]. Crampton et al. [116] noted that two species endemic to seasonally anoxic whitewater floodplains (*B*. *bennetti* and *B*. sp. “flavipomus”) have significantly larger gills than two species endemic to normoxic terra firme streams (*B*. sp. “sullivani”) or blackwater floodplains (*B*. sp. “hendersoni”). *Brachyhypopomus* species also exhibit a reduction of motor and EOD activity under hypoxic conditions, presumably to save metabolic energy [72].

#### Ecologically cosmopolitan species and geographic ranges

We observed a tendency for ecologically eurytopic species (which are tolerant of a range of conductivity, dissolved oxygen, and other conditions) to occupy wider geographical ranges than stenotopic species–matching observations for other gymnotiform species, e.g. *Gymnotus carapo*, and *Sternopygus macrurus*, and other Neotropical fish taxa [22]. For example, several species of *Brachyhypopomus* in Greater Amazonia exhibit ecological distributions that include both high-conductivity, seasonally-hypoxic floodplain systems and low-conductivity, perennially normoxic blackwater or clearwater systems (Figs 19 and 20): *B*. *beebei*, *B*. *brevirostris*, *B*. sp. “hamiltoni”, *B*. sp. “regani”, and *B*. *walteri*. With the exception of *B*. sp. “hamiltoni”, these species exhibit the widest geographical distributions known among congeners.

### Elevated Species Diversity

The 28 species of *Brachyhypopomus* make up 82.4% of the 34 currently known species of Hypopomidae (including the 15 species of *Brachyhypopomus* under description). *Brachyhypopomus* is unmatched in species diversity by any other rhamphichthyoid genus (Hypopomidae: *Akawaio* 1 sp., *Hypopomus* 1 sp., *Microsternarchus* 2 spp., *Racenisia* 1 sp., and *Procerusternarchus* 1 sp.; Rhamphichthyidae: *Gymnorhamphichthys* 6 spp., *Hypopygus* 9 spp., *Iracema* 1 sp., *Rhamphichthys* 9 spp., *Steatogenys* 3 spp.). *Brachyhypopomus* is also one of the most diverse of all gymnotiform genera–surpassed only by *Gymnotus*, with 40 species [77, 117], and *Sternarchorhynchus* with 32 [29], and closely matched only by *Apteronotus*, with 28 species–the monophyly of which is questionable [23], and *Eigenmannia*, with 16 species [118] (or 15 species, see Dutra et al. [119] for comments on the validity of *E*. *goajira*). No other gymnotiform genera exceed 10 species, including taxa that have undergone recent and thorough taxonomic revisions [22].

Albert et al. [53] and de Santana & Vari [29] seek explanations for why *Gymnotus* and *Sternarchorhynchus*, respectively, exhibit elevated species richness. The hypotheses advanced for high species richness in *Sternarchorhynchus* are not applicable to *Brachyhypopomus*. Key ecomorphological innovations such as the grasp-suction feeding mode of *Sternarchorhynchus*, which is postulated to have triggered an adaptive radiation in the genus [29], are lacking in *Brachyhypopomus*. *Sternarchorhynchus* is also inferred to have diversified in part through allopatric speciation in fast-flowing shield headwater systems located above cataracts, but *Brachyhypopomus* is absent or uncommon in these systems.

The reasons for high diversity in *Brachyhypopomus* appear to more closely parallel those for *Gymnotus*. These include a wide geographical range encompassing multiple drainages outside Greater Amazonia (trans-Andean systems included), the occupation of multiple shallow-water habitats, tolerance of hypoxia (which permits the occupation of whitewater river floodplains and terra firme swamps), and high interspecific EOD diversity [2, 53]; data from *Gymnotus* suggest that electric communication signals may play an important role in reproductive isolation and speciation [5, 76, 120]. As in *Gymnotus*, *Brachyhypopomus* generate a diversity of EOD signals–with sympatric species usually exhibiting distinct, species-typical waveforms and/or repetition rates [5, 71, 121–127]. The phylogenetic framework established herein makes the genus *Brachyhypopomus* a superb model system for future investigations of communication signal evolution and species diversification.

## Supporting Information

S1 Appendix List of examined cleared and stained *Brachyhypopomus* specimens. (DOCX)

S2 Appendix List of examined outgroup specimens examined. (DOCX)

S3 Appendix Morphological Synapomorphy scheme for *Brachyhypopomus*. (DOCX)
