# Supplementary material for: Phylogenetic Systematics, Biogeography, and Ecology of the Electric Fish Genus Brachyhypopomus (Ostariophysi: Gymnotiformes)
Source: PLoS One. 2016 Oct 13;11(10):e0161680. doi: 10.1371/journal.pone.0161680 (PMC5063478; doi:10.1371/journal.pone.0161680)
Supplement: S1 Appendix — (DOCX) [file pone.0161680.s001.docx]

**Supplementary Information 1**

**Appendix**

**List of examined cleared and stained *Brachyhypopomus* specimens**

*Brachyhypopomus* sp. “alberti” (n = 1). UMSS 07042, 1 (WC43.060707, male, 84 mm), Bolivia, Beni, Mun. Riberalta, stream nr. Boceron, on Riberalta – Guayaramerín rd., aff. río Beni, aff. rio Madeira, rio Amazonas drainage, 11°02’51”S, 065°50’06”W.

*Brachyhypopomus* sp. “arrayae” (n = 1). UMSS 07047, 1 (WC51.060707, female, 120 mm), Bolivia, Beni, Mun. Riberalta – small stream nr. Village Puerto Hamburgo, aff. río Beni, aff. rio Madeira, rio Amazonas drainage, 11°01’52”S, 066°05’39”W.

*Brachyhypopomus* sp. “batesi” (n = 1). MCP 45356, 1 (WC11.231297b, female, 85 mm), Brazil, Amazonas, Mun. Tefé, igarapé Xidarini, aff. lago Tefé (mouthbay of rio Tefé), rio Amazonas drainage, 03°23’52”S, 064°41’17”W.

*Brachyhypopomus beebei* (n = 8). KU 12741, 1, 90 mm; KU 13800, 4, 50-145 mm, Ecuador, Napo, swamp, S. air strip at Santa Cecília, río Aguarico, aff. río Napo, rio Amazonas drainage, ca. 03°52’S, 076°59’W. MCP 45381, 1 (WC03.110597, immature, 140 mm TL), Brazil, Amazonas, Mun. Alvarães, Jarauá Lake System, Ressaca do Caetono, rio Solimões – rio Japurá floodplain, rio Amazonas drainage, 02º50’15”S, 064º55’50”W. MCP 45387, 1 (WC01.200598, immature, 97 mm), Brazil, Amazonas, Mun. Alvarães, Mamirauá Reserve, lago Periquito Comprido, rio Solimões – rio Japurá floodplain, rio Amazonas drainage, 03º04’57”S, 064°46’42”W. MCP 45421, 1 (WC04.160698, male, 201 mm), Brazil, Amazonas, Mun. Alvarães, Mamirauá Reserve, cano do lago Mamirauá, rio Solimões – rio Japurá floodplain, rio Amazonas drainage, 03º04’29”S, 064°48’29”W. MCP 45427, 1 (WC03.130300, female, 136 mm), Mun. Tefé, igarapé Curupira, at bridge on Estrada Agrovila, lago Tefé, rio Tefé, rio Amazonas drainage, 03º25’48”S, 064º44’15”W. MCP 45450, 4 (WC06.090600, female, 152 mm; WC07.090600, female, 166 mm; 2 immature unrecorded 102- 195 mm), Brazil, Amazonas, Mun. Alvarães, Mamirauá Reserve, lago Secretaria, rio Solimões – rio Japurá floodplain, rio Amazonas drainage, 03º07’12”S, 064°47’49”W.

*Brachyhypopomus* sp. “belindae” (n = 1). MCP 45430, 1 (WC03.150699, immature, 102 mm). Brazil, Amazonas, Mun. Alvarães, Mamirauá Reserve, lago Araçazinho, rio Solimões – rio Japurá floodplain, rio Amazonas drainage, 02º59’35”S, 064º51’33”W.

*Brachyhypopomus* sp. “benjamini” (n = 1). UF 183771, 1, immature, 81 mm, Peru, Loreto, río Nanay, 50 km 250° from Iquitos, rio Amazonas drainage, 03°53’50”S, 073°40’01”W.

*Brachyhypopomus bennetti* (n = 7). MCP 45346, 1 (WC04.290696, female, 156 mm), Brazil, Amazonas, Mun. Alvarães, Mamirauá Reserve, lago Juruá Grande, rio Solimões – rio Japurá floodplain, rio Amazonas drainage, 03º01’51”S, 064º51’07”W. MCP 45359, 3 (WC01. 020497, female, 143 mm; WC17.050497, male 190 mm; WC02.090497, female, 145 mm), Brazil, Amazonas, Mun. Alvarães, Mamirauá Reserve, lago Araçazinho, rio Solimões – rio Japurá floodplain, rio Amazonas drainage, 02º59’35”S, 064º51’33”W. MCP 45252, 1 (WC02.150598, immature, 83 mm), Brazil, Amazonas, Mun. Alvarães, Mamirauá Reserve, lago Juruazinho, rio Solimões – rio Japurá floodplain, rio Amazonas drainage, 03º02’41”S, 064º51’26”W. MCP 45254, 1 (WC13.310598, female, 141mm), Brazil, Amazonas, Mun. Alvarães, Mamirauá Reserve, lago Geraldo, rio Solimões – rio Japurá floodplain, rio Amazonas drainage, 03º06’53”S, 064°49’06”W. MCP 46934, 1, 73 mm, Brazil, Amazonas, Mun. Alvarães, Mamirauá Reserve, cano do lago Rato, rio Solimões – rio Japurá floodplain, rio Amazonas drainage, 03º02’41”S, 064º51’26”W.

*Brachyhypopomus bombilla* (n = 5). CBF 10273, 1 (WC02.240607, immature, 91 mm); CBF 10274, 1 (WC03.240607, male, 96 mm), Bolivia, Beni, Mun. Riberalta, arroio Tres Cuchillos, río Beni floodplain, río Beni, aff. rio Madeira, rio Amazonas drainage, 10°51’44.3”S, 066°04’50.3”W. MCP 19847, 2, 127-140 mm, Brazil, Rio Grande do Sul, bridge over Banhado do Inhatium, hwy. BR 290, 21 km, 295° from São Gabriel, aff. rio Ibicuí, aff. rio Uruguay drainage, 30°15’43”S, 054°31’33”W. UF 183773, 1 (WC02.231106, immature, 105 mm), Uruguay, Durazno, Laguna Lavalle, río Negro, rio Uruguay drainage, 33°01’16”S, 055°22’30”W.

*Brachyhypopomus brevirostris* (n = 13). MCP 46930, 2, (WC10.250693, immature, 178 mm; WC01.260793, immature, 110 mm), Brazil, Amazonas, Mun. Alvarães, Mamirauá Reserve, cano do lago Mamirauá: rio Solimões – rio Japurá floodplain, rio Amazonas drainage, 03º03’51”S, 064º51’05”W. MCP 46927, 1 (WC09.140893, immature, 136 mm), Brazil, Amazonas, Mun. Alvarães, Mamirauá Reserve, lago Bolsinha, rio Solimões – rio Japurá floodplain, rio Amazonas drainage, 03º03’47”S, 064º50’03”W. MCP 44605, 1 (WC06.010596, male, 374 mm), Brazil, Amazonas, Mun. Tefé, Ressaca do Cachorro, lago Tefé, rio Amazonas drainage, 03°19’50”S, 64°42’15”W. MCP 44606, 3 (WC13.180197, male, 308 mm, WC06.310197, immature, 175 mm; WC07.310197, unrecorded immature, 124 mm), Brazil, Amazonas, Mun. Tefé, lago Tefé, Cabeçeira do lago Tefé, rio Tefé, rio Amazonas drainage, 03°36’55”S, 064°57’33”W. MCP 44758, 2 (WC03.030597, female, 262 mm; WC07.030597, immature, 257 mm), Brazil, Amazonas, Mun. Alvarães, Mamirauá Reserve, Ressaca da Vila Alencar, rio Solimões – rio Japurá floodplain, rio Amazonas drainage, 03º07’41”S, 064°48’04”W. MCP 44759, 2 (immature, 47.7 mm, immature 110), Brazil, Amazonas, Mun. Tefé, Ressaca do Cachorro, lago Tefé, rio Amazonas drainage, 03°19’50”S, 64°42’15”W. MCP 46935, 1 (post-larval, 30 mm), Brazil, Amazonas, rio Amazonas drainage. Brazil, Amazonas, Mun. Alvarães, Mamirauá Reserve, cano do lago Mamirauá: rio Solimões – rio Japurá floodplain, rio Amazonas drainage, 03º03’51”S, 064º51’05”W. UF 176887, 2 unsexed, 75.5-105 mm, Venezuela, Apure, Hato El Frio, nr. Mantecal, floodplain lakes of río Guaratico, aff. río Apure, río Orinoco drainage, 07°49’32”N, 068’55’23”W.

*Brachyhypopomus bullocki* (n = 2). UF 177348, 2 (WC02.110304, immature, 191 mm; WC04.110304, immature, 128 mm), Venezuela, Amazonas, Pozo “CVG” (Corporacion Venezuela Guyana) on rd. from San Fernando de Atabapo to Santa Barbara, 10.5 km, 140° from San Fernando de Atabapo, aff. río Orinoco, río Orinoco drainage, 03°58’59”N, 067°38’27”W.

*Brachyhypopomus* sp. “cunia” (n = 3). MCP 46937, 2, 92-118 mm; INPA 37691, 1 female, 121 mm: Brazil, Rondônia, Mun. Calamã, floodplains lakes of rio Madeira at Calamã, rio Amazonas drainage, 08º01’37”S, 062°52’28”W.

*Brachyhypopomus diazi* (n = 7). UF 176888, 3 (WC02.020403, female, 106 mm; WC06.020403, female, 96 mm; WC14.020403, female, 80 mm), Venezuela, Apure, Hato El Frio, nr. Mantecal, floodplain lakes of río Guaratico, aff. río Apure, río Orinoco drainage, 07°49’32”N, 068’55’23”W. UF 174333, 4 (WC05.210304, female, 100 mm; WC06.210304, immature, 132 mm; WC09.210304, male, 121 mm; unrecorded post-larval 20 mm), Venezuela, Carabobo, Mun. Morón, río Alpargatón, at rd. and railway bridge on hwy. 1, 8.4 km, 265° from Morón, río Salado drainage, 10°27’58”N, 068°15’38”W.

*Brachyhypopomus draco*. MCP 19846, 1, female, 130 mm, Brazil, Rio Grande do Sul, Bridge over Banhado do Inhatium, hwy. BR 290, 21 km, 295° from São Gabriel, aff. rio Ibicuí, aff. rio Uruguay drainage, 30°15’43”S, 054°31’33”W.

*Brachyhypopomus* sp. “flavipomus” (n = 4). MCP 45365, 4 (WC28.110597, male, 97 mm; WC05.150597, female, 103 mm; WC06.150597, female, 101 mm; WC09.150597, female, 95 mm), Brazil, Amazonas, Mun. Alvarães, Jarauá Lake System, Ressaca do Caetono, rio Solimões – rio Japurá floodplain, rio Amazonas drainage, 02º50’15”S, 064º55’50”W.

*Brachyhypopomus gauderio* (n = 5). MCP 14606, 1 unsexed, 112 mm, Brazil, Rio Grande do Sul, drainage channel below hwy. BR-290, ca. 2 km from BR-116, aff. rio Jacuí, rio Guaíba drainage, 30°02’46”S, 051°21’07”W. UF 177364, 1 unsexed, 150 mm, Argentina, Entre Rios, nr. La Paz, floodplain of río Paraná, rio Paraná drainage, ca. 30°42’S, 059°39’W. UF 183779, 1, immature 153 mm, captive bred specimen at P. Stoddard Lab, Florida International University – population originally exported from Uruguay. UF 183774, 2, immature, 118-155 mm, Uruguay, Durazno, Laguna Lavalle, río Negro, rio Uruguay drainage, 33°01’16”S, 055°22’30”W.

*Brachyhypopomus* sp. “hamiltoni” (n = 4). MCP 45250, 1, (WC04.261198, immature, 80 mm), Brazil, Amazonas, Mun. Maraã, Boca do igarapé Juá Grande, lago Amanã, aff. rio Japurá, rio Amazonas drainage, 02°27’14”S, 064°48’26”W. MCP 45302, 3 (WC05.031298, immature, 89 mm; WC10.031298, immature, 75 mm TL; WC12.031298, 1, 85 mm), Brazil, Amazonas, Mun. Maraã, igarapé Juá Grande, lago Amanã, aff. rio Japurá, rio Amazonas drainage, 02°28’50”S, 064°48’50”W.

*Brachyhypopomus* sp. “hendersoni” (n = 7). MCP 45271, 1 (WC09.080197, immature, 132 mm), Brazil, Amazonas, Mun. Tefé, Ressaca do Socorro, lago Tefé, rio Tefé, rio Amazonas drainage, 03°18’45”S, 64°41’50”W. MCP 45304, 1 (WC02.011298, immature, 100 mm TL), Brazil, Amazonas, Mun. Maraã, lago Amanã, mouth of igarapé Uxi, aff. rio Japurá, rio Amazonas drainage, 02°32’41”S, 064°40’12”W. MCP 45432, 2 (WC05.130799, male, 161 mm; WC06.130799, female, 142 mm), Brazil, Amazonas, Mun. Tefé, rio Tefé, rio Amazonas drainage, 03º37’43”S, 064°59’03”W. MCP 45426, 1 (WC16.140799, female, 166 mm), Brazil, Amazonas, Mun. Tefé, lago Tefé, Cabeçeira do lago Tefé, rio Tefé, rio Amazonas drainage, 03°38’01”S, 064°57’59”W. MCP 45454, 2 (WC01.160200, male, 175 mm; WC05.160200, female, 127 mm), Brazil, Amazonas, Mun. Tefé, lago Tefé, Cabeçeira do lago Tefé, rio Tefé, rio Amazonas drainage, 03°38’01”S, 064°57’59”W.

*Brachyhypopomus janeiroensis* (n = 2). MZUSP 80122, 1, 174 mm; UF 183780, 1, 120 mm, Brazil, Rio de Janeiro, Mun. Silva Jardim. aff. São João, 28 km N. by estrada Boqueirao-Japuiba, at Gaviões, rio São João drainage, 22°34’S, 042°34’W.

*Brachyhypopomus jureiae* (n = 1). MZUSP 93118, 1 (WC02.090706, male, 170 mm), Brazil, São Paulo, stream, aff. rio Momuna, ca. 1.5 km from Vila de Momuna, rio Ribeira de Iguape drainage, 24°42’29”S, 47°40’53”W.

*Brachyhypopomus* sp. “menezesi” (n = 1). MZUSP 40190, 1 unsexed, 111 mm, Brazil, Bahia, Mun. Ibiraba, nr. mouth of rio Icatú, rio São Francisco drainage, 10°33’S, 043°06’W.

*Brachyhypopomus occidentalis* (n = 3). UF 21796, 1, immature, 120 mm, Venezuela, Zulia, stream about 10 km N. Mene Grande, lago de Maracaibo drainage, ca. 09°55’N, 070°55’W. USNM 293152, 1, immature, 156 mm, Panamá, San Blas, río Cartí, 3 km from Cartí, río Cartí drainage, 09°26’N, 078°58’N. USNM 302001, 1, immature, 102 mm, Panamá, San Blas, río Chucubti, nr. Obaldía [Puerto Obaldía], río Chucubti drainage, ca. 08°39’N, 077°25’W.

*Brachyhypopomus* sp. “palenque” (n = 3). UF 180271, 1 (WC08.160404, immature, 31 mm), Ecuador, Los Ríos, Mun. Buena Fé, Parroquia Patricia Pilar, Centro Científico río Palenque, 45 km S. Santo Domingo, small rain forest stream, aff. río Palenque, río Guayas drainage, 00°35’01”S, 079°22’13”W. USNM 270692, 2, 163 – 202 mm, “río Sigchos, aff. río Esmeraldo” [interpreted as río Toachi nr. town Sigchos, Cotopaxi, aff. río Esmeraldas], no coordinates.

*Brachyhypopomus pinnicaudatus* (n = 5). MCP 45370, 2 (WC04.010497, female, 125 mm; WC03.050497, female, 122 mm), Brazil, Amazonas, Mun. Alvarães, Mamirauá Reserve, lago Araçazinho, rio Solimões – rio Japurá floodplain, rio Amazonas drainage, 02º59’35”S, 064º51’33”W. MCP 45398, 1 (WC03.160598, female, 116 mm), Brazil, Amazonas, Mun. Alvarães, Mamirauá Reserve, lago Juruá Grande, rio Solimões – rio Japurá floodplain, rio Amazonas drainage, 03º01’51”S, 064º51’07”W. MCP 46928, 2 (WC06.150199, female, 148 mm; WC02.160199, female, 143mm), Brazil, Amazonas, Mun. Alvarães, Mamirauá Reserve, lago Secretaria, rio Solimões – rio Japurá floodplain, rio Amazonas drainage, 03º07’12”S, 064°47’49”W.

*Brachyhypopomus* sp. “provenzanoi” (n = 4). UF 177347, 3 immature, 67 – 87 mm, Venezuela, Amazonas, Mun. San Fernando de Atabapo, caño “CVG” (Corporacion Venezuelana Guyana), 10.5 km, 140° from San Fernando on rd. to Santa Barbara, aff. río Orinoco, río Orinoco drainage, 03º58’59”N, 067º38’29”W. MBUCV-V 35651, 1 immature, 75 mm: Venezuela, Amazonas, caño Viejita, on rd. from San Fernando de Atabapo to Santa Barbara, aff. río Atabapo, río Orinoco drainage, 03°55’59”N, 067°36’34”W.

*Brachyhypopomus* sp. “regani” (n = 3). MCP 45457, 1, male, 130 mm, Brazil, Amazonas, Mun. Alvarães, Mamirauá Reserve, Ressaca da Vila Alencar, rio Solimões – rio Japurá floodplain, rio Amazonas drainage, 03º07’41”S, 064°48’04”W. MCP 45484, 2 (WC04.150301, immature, 90 mm; WC09.150301, immature, 105 mm), Brazil, Amazonas, Mun. Alvarães, Mamirauá Reserve, cano do lago Mamirauá, rio Solimões – rio Japurá floodplain, rio Amazonas drainage, 03º06’40”S, 064°47’52”W.

*Brachyhypopomus* sp. “sullivani” (n = 11). FMNH 70075, 2, 60-65 mm, Brazil, Pará, Missão do Cururu, rio Cururu, aff. rio Tapajós, rio Amazonas drainage, 07°36’50”S, 057°38’58”W. INPA 11595, 3, 85-94 mm, Brazil, Mato Grosso, igarapé do aeroporto, cidade de Humboldt [Aripuanã], rio Aripuanã, aff. rio Madeira, rio Amazonas drainage, 10°11’18”S, 059°27’47”W. MCP 45486, 3 (WC02.221299, male, 101 mm; WC01.310100, male, 101 mm; WC02.310100, female, 86 mm), Brazil, Amazonas, Mun. Tefé, igarapé Repartimento, 1.5 km downstream from Estrada Agrovila, rio Tefé, rio Amazonas drainage, 03º24’25”S, 064º44’08”W. UF 177355, 3, immature, 70 – 97 mm, Venezuela, Amazonas, on rd. from Puerto Ayacucho to Gavilan, 27.7 km, 122° from Puerto Ayacucho town center caño Moia, aff. río Cataniapo, río Orinoco drainage, 05°32’26”N, 067°23’02”W.

*Brachyhypopomus* sp. “verdii” (n = 3). UF 148520, 3 (WC20.090104, immature, 93 mm, 2 immature unrecorded, 70-80 mm), Peru, Loreto, Mun. Requena, stream in forest ca. 2 km N. Instituto de Investigaciones de la Amazonia Peruana (IIAP) field station (2.7 km E. Jenaro Herrera), aff. río Ucayali, rio Amazonas drainage, 04º53’S, 073º39’W.

*Brachyhypopomus walteri* (n = 8) MCP 44607, 1 (WC05.200598, female, 150 mm), Brazil, Amazonas, Mun. Alvarães, Mamirauá Reserve, lago Periquito Comprido, rio Solimões – rio Japurá floodplain, rio Amazonas drainage, 03º04’57”S, 064°46’42”W. MCP 44742, 1 (WC07.040598, female, 104 mm), Brazil, Amazonas, Mamirauá Reserve, lago Periquito Comprido, rio Solimões – rio Japurá floodplain, rio Amazonas drainage, 03º04’57”S, 064°46’42”W. MCP 44649, 2 (WC04.020698, male, 185mm; WC05.020698, female, 140 mm), Brazil, Amazonas, Mun. Alvarães, Mamirauá Reserve, lago Curuçá Comprido, rio Solimões – rio Japurá floodplain, rio Amazonas drainage, 03º05’31”S, 064°48’58”W. MCP 44741, 1 (WC01.201296, immature, 146 mm), Brazil, Amazonas, Mun. Tefé, igarapé Repartimento, 1.5 km downstream from Estrada Agrovila, rio Tefé, rio Amazonas drainage, 03º24’25”S, 064º44’08”W. MCP 44743, 2 (WC01.130799, immature, 124 mm; WC17.130799, 1, 103 mm), Brazil, Amazonas, Mun. Tefé, rio Tefé, rio Amazonas drainage, 03º37’43”S, 064°59’03”W. MCP 46933, 1, 70 mm, Brazil, Amazonas, Amazon drainage, Mun. Alvarães, Mamirauá Reserve, lago Rato, rio Solimões – rio Japurá floodplain, rio Amazonas drainage, 03º03’01”S, 064º52’23”W.

*Brachyhypopomus* sp. indet. (n = 4). FMNH 96986, 1, 121 mm, Peru, Amazonas, Caterpiza, Río Caterpiza, aff. Río Marañon, Rio Amazonas drainage, ca. 03°55’S, 077°44’W. MCP 44760, 2, 37-39 mm; MCP 46021 (illustrated in de Santana & Crampton, 2011, 1104, fig. 2), 1, 74 mm, Brazil, Amazonas, Mun. Alvarães, Ressaca da Vila Alencar, Rio Solimões – Rio Japurá floodplain, Rio Amazonas drainage, 03º07'41"S, 064°48'04"W.
