# Supplementary material for: Phylogenetic Systematics, Biogeography, and Ecology of the Electric Fish Genus Brachyhypopomus (Ostariophysi: Gymnotiformes)
Source: PLoS One. 2016 Oct 13;11(10):e0161680. doi: 10.1371/journal.pone.0161680 (PMC5063478; doi:10.1371/journal.pone.0161680)
Supplement: S2 Appendix — (DOCX) [file pone.0161680.s002.docx]

**Supplementary Information 2**

**Appendix**

**List of examined outgroup specimens examined** (including cleared and stained specimens)

***Gymnorhamphichthys***

*Gymnorhamphichthys rondoni* (Miranda-Ribeiro) (n = 6). MCP 44603, 4 (paratypes), 83-157 mm (1 CS WC01.240899, 128 mm), MCP 46936, 2, 161-164 mm (1 CS, 161 mm), Brazil, Amazonas, Mun. Tefé, igarapé Repartimento, near Tefé, rio Amazonas drainage, 03º24’27”S, 064º44'10”W.

***Gymnotus***

*Gymnotus jonasi* Albert and Crampton (n = 6). INPA 13507, 1 (holotype), 114 mm, Brazil, Amazonas, Mun. Alvarães, Mamirauá Lake System, Cano do Lago Rato, rio Solimões – rio Japurá floodplain, rio Amazonas drainage, 03º02'41"S, 064º51'26"W. MCP 46931, 3 (CS), 42-106 mm, Brazil, Amazonas, Amazon drainage, rio Solimões-Japurá floodplain, Paraná Maiana, rio Amazonas drainage, 03º06'44”S, 064°47'32”W. MZUSP 103220, 1, 121 mm, Brazil, Amazonas, Mun. Alvarães, Ressaca da Vila Alencar, rio Solimões – rio Japurá floodplain, rio Amazonas drainage, 03°07’42”S, 064°48’02”W. UF 131410 (WC01.140503), 1, male, 129 mm, Peru, Loreto, cocha Yarina, Pacaya Samiria National Reserve, R. Ucayali, R. Amazonas drainage, 05°24'37”S, 074°30'15”W.

***Hypopomus***

*Hypopomus artedi* Kaup (n = 13). ANSP 179505, 1, 136 mm; AUM 35574, 1, 130 mm, Guyana, Cuyuni-Mazaruni, 6.8 km SW Bartica, “whitewater creek”, trib. Mazaruni River, Essequibo River drainage, 06°22' 41"N, 058°40'25”W. NRM 32247, 1 (CS), 31.4 mm. French Guiana, Cayenne, Fleuve Oyapock drainage, tributary of Oyapock, no coordinates. UF 176889, 10, 215-430 mm (2 CS, WC05.070307, male, 242 mm, WC06.070307, male, 225 mm), Suriname, Brokopondo, Marshall Kreek, Affobaka road from Paranam refinery to Brokopondo reservoir, Suriname Rivier drainage, 05°14’42”N, 055º06’04”W.

***Hypopygus***

*Hypopygus lepturus* Hoedeman (n = 17). MCP 44755, 1 (CS), 30 mm, Brazil, Amazonas, igarapé Baré, aff. Lago Amanã, aff. rio Japurá, rio Amazonas drainage, 02°26’17”S, 064°43’30”W. RMNH 19466, 1 (holotype), 74 mm, Suriname Maroni drainage, no exact locality. UF 176800, 1, 50 mm, Peru, Loreto, R. Nanay, 50 km 250° from Iquitos, R. Amazonas drainage, 03°53’50”S, 073°40’01”W. UF 176882, 2, 91-92 mm (1 CS, WC09.050307, immature, 92 mm), Suriname, Para, Mun. Zanderij, Kola Kreek, ca. 6km W. village of Zanderij and Johan Adolf Pengel International Airport, Suriname R. drainage, 05°27’09”N, 055º14’42”W. UF 176884, 12, 44-99 mm (1CS, WC25.0903071, immature, 91 mm), Suriname, Marowijne, small stream on Paramaribo-French Guiana rd., Cottica Riviere, Commewijne Rivier drainage, 05°35’13”N, 054°17’07”W.

***Microsternarchus***

*Microsternarchus bilineatus* Fernández-Yépez (n = 15). MCP 44653, 3 CS, immature, 23–44 mm, Brazil, Amazonas, Amazonas drainage, nr. Tefé, igarapé Repartimento, aff. Lago Tefé, aff. rio Tefé drainage, 03°24′30”S, 064°44′12”W. MCP 45463, 1, 82 mm, Brazil, Amazonas, Mun. Tefé, igarapé Curupira, aff. Lago Tefé (rio Tefé), R. Amazonas drainage, 03°26’01”S, 064°43’47”W. MCP 45480 (WC02.200201), 1, 93 mm, Brazil, Amazonas, Mun. Tefé, igarapé Repartimento, aff. Lago Tefé (rio Tefé), R. Amazonas drainage, 03°24’25”S, 064°44’08”W. UF 176885, 2, 82-92 mm, Venezuela, Amazonas, Mun. San Fernando de Atabapo, río Orinoco drainage: on rd. to Santa Barbara, 03°59’00”N, 067°38’29”W. UF 176886, 10, 75-111 mm (5 CS, immature, 75-111 mm), Venezuela, Amazonas, Mun. San Fernando de Atabapo, Caño Cascaradura, near San Fernando de Atabapo, río Orinoco drainage, 04°00’20”N, 067°39’42”W.

***Procerusternarchus***

*Procerusternarchus pixuna* Fernandes, Nogueira & Alves-Gomes (n = 3). MZUSP 80140, 3, 81-116 mm, Brazil, Amazonas, igarapé Mipiriyapotemakaya, igarapé Açai, rio Tiquié, rio Negro, rio Amazonas drainage, 00°15’55”N, 069°58’16”W.

***Racenisia***

*Racenisia fimbriipinna* Mago-Leccia (n = 78). MBUCV-V 7127, 23 (paratypes), 47-90 mm; MBUCV-V 7540, 1 (holotype), 117 mm, Venezuela, Amazonas, El Pozo de Lucas, San Fernando de Atabapo, río Orinoco drainage, ca. 04°15’N, 067°39’W. UF 177352, 54, 42-95 mm (14 CS, WC12.120304, immature, 90 mm; WC04.130303, immature, 91 mm; 12 unrecorded 44 – 85 mm), Venezuela, Amazonas, Mun. San Fernando de Atabapo, 16.5 km and 142 degrees from San Fernando de Atabapo, Caño Viejita, aff. R. Orinoco, R. Orinoco drainage, 03°55’59”N, 067°36’34”W.

***Rhamphichthys***

*Rhamphichthys marmoratus* Castelnau (n = 5). MCP 44604, 2 CS, 43.4-43.4 mm (WC01.020195, immature, 43.4 mm; WC02.020195, immature, 43.4 mm), Brazil, Amazonas, Mun. Alvarães, Lago Juruazinho, rio Japurá-rio Solimões floodplain, rio Amazonas drainage, 03º02'35”S, 064º51'01”W. MCP 44756, 1 CS, 159 mm (WC01.290594, immature), Brazil, Amazonas, Mun. Tefé, boca do igarapé Xidarini, aff. Lago Tefé (rio Tefé), rio Amazonas drainage, 03°21’37”S, 064°41’53”W. MCP 46929, 1, 465 mm; MCP 46932, 1, 515 mm, Brazil, Amazonas, Mun. Alvarães, Praia Caborini, rio Japurá-Solimões confluence, rio Amazonas drainage, 03°07’09”N, 064°47’18”W.

***Steatogenys***

*Steatogenys duidae* La Monte (n = 17). MCP 31957 2 CS (WC01.300600, immature, 104 mm; WC02.300600, immature, 100 mm); MCP 31960, 1 CS (WC01.210201), 178 mm, Brazil, Amazonas, Mun. Tefé, igarapé Repartimento, aff. Lago Tefé (rio Tefé), R. Amazonas drainage, ca. 03°24’25”S, 064°44’08”W. MCP 31958, Brazil, Amazonas, Mun. Barcelos, R. Demini, aff. R. Negro, R. Amazonas drainage, 00°21’50”S, 062°49’28”W. UF 148542, 16, 97-181 mm (1 CS, 90 mm), Venezuela, Amazonas, Mun. San Fernando de Atabapo, río Orinoco drainage, Caño Viejita on rd. to Santa Barbara, 03°55’59”N, 067°36’34”W.

***Sternopygus***

*Sternopygus astrabes* Mago-Leccia (n = 20). MCP 32231 (WC16.240899), 1, 75 mm, Brazil, Amazonas, Mun. Tefé, igarapé Repartimento, aff. Lago Tefé (rio Tefé), R. Amazonas drainage, 03°24’25”S, 064°44’08”W. MCP 32235 (WC07.291000), 134 mm, Brazil, Amazonas, Mun. Tefé, igarapé Repartimento, aff. Lago Tefé (rio Tefé), R. Amazonas drainage, 03°24’25”S, 064°44’08”W. UF 177351, 18, 117-238 mm (2 CS, immature, 125-155 mm), Venezuela, Amazonas, San Fernando de Atabapo, río Orinoco drainage: Caño Viejita on rd. to Santa Barbara, 03°55’59”N, 067°36’34”W.
