# Supplementary material for: Phylogenetic Systematics, Biogeography, and Ecology of the Electric Fish Genus Brachyhypopomus (Ostariophysi: Gymnotiformes)
Source: PLoS One. 2016 Oct 13;11(10):e0161680. doi: 10.1371/journal.pone.0161680 (PMC5063478; doi:10.1371/journal.pone.0161680)
Supplement: S3 Appendix — (DOCX) [file pone.0161680.s003.docx]

**Supplementary Information 3**

**Appendix**

**Morphological Synapomorphy scheme for *Brachyhypopomus***

The lettered and numbered nodes defining clades listed here refer to the total evidence consensus tree for phylogenetic interrelationships among *Brachyhypopomus* spp. (Figs.5,7). Clades that are well-supported (node support exceeding 0.88 Bayesian Posterior Probability [PP]) are labeled with letters, A-W. Clades with weaker support (PP < 0.88) are labeled with numbers, 1-3 (for node-support, see Fig. 6; for clade labels see Fig. 7). Here we list character state transitions that optimize as unambiguous or ambiguous synapomorphies at each node, and also transitions that optimize as autapomorphies for species. We limit the scheme to characters pertinent to interrelationships within *Brachyhypopomus* and to the diagnosis of the genus.

**Clade — *Brachyhypopomus***

(28 species; bootstrap support 98%, decay index 12)

Character 32, state 0 → 1 (unambiguous, homoplastic); *gill rakers funnel-shaped*.

Character 41, state 0 → 1 (ambiguous, non-homoplastic); *mesocoracoid bridge distinctly curved*.

Character 47, state 0 → 1 (ambiguous, homoplastic); *descending process of the maxilla narrow*.

Character 57, state 0 → 1 (unambiguous, non-homoplastic); *anterior portion of the palatoquadrate cartilage ossifies in adults*.

**Clade 1:**

(22 species; bootstrap support 62%, decay index 2): *Brachyhypopomus* sp. ALBE, *B.* sp. ARRA, *B.* sp. BATE, *B. beebei*, *B.* sp. BELI, *B.* sp. BENJ, *B. bennetti*, *B. bombilla*, *B. diazi, B. draco*, *B.* sp. FLAV, *B. gauderio*, *B.* sp. HAMI, *B. occidentalis*, *B. pinnicaudatus*, *B.* sp. MENE, *B.* sp. PALE, *B.* sp. PROV, *B.* sp. REGA, *B.* sp. SULL, *B.* sp. VERD, *B. walteri*.

Character 38, state 0 → 1 (ambiguous, homoplastic); *mesocoracoid bridge contacting scapula.*

**Clade A**

(12 species; bootstrap support 95%, decay index 5): *Brachyhypopomus* sp. ALBE, *B.* sp. ARRA, *B. beebei*, *B.* sp. BELI, *B. bennetti*, *B. draco*, *B.* sp. FLAV, *B. gauderio*, *B.* sp. HAMI, *B. pinnicaudatus*, *B.* sp. VERD,  *B. walteri*.

Character 19, state 0 → 1 (unambiguous, homoplastic); *interhyal* *cone-like.*

Character 23, state 0 → 1 (unambiguous, homoplastic); *first branchiostegal ray distinctly narrower than third ray*.

Character 33, 0 → 1 (unambiguous, homoplastic); *absence of the fourth proximal radial*.

**Clade B**

(10 species; bootstrap support 98%, decay index 5): *Brachyhypopomus* sp. ALBE, *B.* sp. ARRA, *B. beebei*, *B.* sp. BELI, *B. draco*, *B.* sp. FLAV, *B. gauderio*, *B.* sp. HAMI, *B. pinnicaudatus*, *B.* sp. VERD.

Character 17, state 0 → 1 (unambiguous, homoplastic); *lateral ethmoid narrow, and tube-shaped*.

Character 31, state 0 → 1 (ambiguous, homoplastic); *absence of gill rakers*.

Character 48, state 0 → 1 (unambiguous, homoplastic); *preopercular sensory canals independent of preopercle*.

**Clade C**

(7 species bootstrap support 88%, decay index 3): *Brachyhypopomus* sp. ALBE, *B.* sp. ARRA, *B. beebei*, *B.* sp. BELI, *B. draco*, *B.* sp. HAMI, *B.* sp. VERD.

Character 6, state 0 → 1 (ambiguous, homoplastic); *absence of branch of infraorbital canal over frontal.*

**Clade D (beebei Species Group)**

(5 species; bootstrap support 84%, decay index 3): *Brachyhypopomus* sp. ALBE, *B.* sp. ARRA, *B. beebei*, *B. draco*, *B.* sp. HAMI.

Character 31, state 1 → 0 (ambiguous, homoplastic); *presence of gill rakers.*

**Clade E**

(3 species; bootstrap support 88%, decay index 3): *Brachyhypopomus* sp. ALBE, *B.* sp. ARRA, *B. draco*.

Character 20, state 0 → 1 (ambiguous, non-homoplastic); *anterior extension in cone-like interhyal curved*.

Character 23, state 1 → 0 (unambiguous, homoplastic); *first branchiostegal ray approximately as wide as third ray.*

**Clade F**

(2 species; bootstrap support 78%, decay index 2): *Brachyhypopomus* sp. ALBE, *B.* sp. ARRA.

Character 31, state 0 → 1 (ambiguous, homoplastic); *absence of gill rakers*.

Character 56, state 0 → 1 (unambiguous, homoplastic); *ascending process on the endopterygoid forms a contact with the orbitosphenoid*.

Character 59, state 0 → 1 (unambiguous, non-homoplastic); *females possess distinctly darker pigmentation than males*.

***Brachyhypopomus* sp. ALBE**

Character 22, state 0 → 1 (unambiguous, homoplastic); *absence of first branchiostegal ray*.

***Brachyhypopomus* sp. ARRA**

Character 26, state 0 → 1 (unambiguous, homoplastic); *absence of medial bridge on posterior portion of basihyal*.

***Brachyhypopomus draco***

Character 6, state 1 → 0 (ambiguous, homoplastic); *branch of the infraorbital canal over the frontal present.*

Character 17, state 1 → 0 (unambiguous, homoplastic); *lateral ethmoid wide*.

Character 19, state 1 → 0 (unambiguous, homoplastic); *interhyal uneven, non-cone-like shape*.

Character 25, state 1 → 0 (unambiguous, homoplastic); *basihyal smaller than first ceratohyal.*

**Clade G**

(2 species; bootstrap support 97%, decay index 3): *Brachyhypopomus beebei*, *B.* sp. HAMI.

No morphological synapomorphies.

***Brachyhypopomus beebei***

Character 6, state 1 → 0 (ambiguous, homoplastic); *branch of the infraorbital canal over the frontal present.*

Character 22, state 0 → 1 (unambiguous, homoplastic); *absence of first branchiostegal ray*.

Character 36, state 0 → 1 (unambiguous, homoplastic); *presence of foramen on coracoid*.

***Brachyhypopomus* sp. HAMI**

Character 32, state 1 → 0 (unambiguous, homoplastic); *gill rakers in form of a crown with small teeth*.

Character 35, state 0 → 1 (unambiguous, homoplastic); *anterior portion of first through third proximal radials in contact*.

Character 56, state 0 → 1 (unambiguous, homoplastic); *ascending process on endopterygoid contacting orbitosphenoid*.

**Clade H (sp. BELI Species Group)**

(2 species; bootstrap support 100%, decay index 4): *Brachyhypopomus* sp. BELI, *B.* sp. VERD*.*

Character 22, state 0 → 1 (unambiguous, homoplastic); *absence of first branchiostegal ray.*

Character 61, state 1 → 0 (unambiguous, homoplastic); *high number of precaudal vertebrae.*

***Brachyhypopomus* sp. BELI**

Character 19, state 1 → 0 (unambiguous, homoplastic); *interhyal uneven, non-cone-like shape*.

Character 30, state 0 → 1 (unambiguous, homoplastic); *fourth basibranchial ossified*.

Character 56, state 0 → 1 (unambiguous, homoplastic); *ascending process on endopterygoid forms a contact with orbitosphenoid*.

***Brachyhypopomus* sp. VERD**

Character 9, state 0 → 1 (unambiguous, homoplastic); *supraorbital canal independent from frontal.*

Character 33, state 1 → 0 (unambiguous, homoplastic); *presence of fourth proximal radial*.

Character 54, state 1 → 0 (unambiguous, homoplastic); *neural complex not contacting exoccipital*.

Character 55, state 0 → 1 (unambiguous, non-homoplastic); *anterior portion of neural complex concave*.

**Clade 2**

(3 species; bootstrap support 60%, decay index 2): *Brachyhypopomus* sp. FLAV, *B. gauderio*, *B. pinnicaudatus*.

Character 26, state 0 → 1 (ambiguous, homoplastic); *absence of medial bridge on posterior portion of basihyal*.

**Clade I (pinnicaudatus Species Group)**

(2 species; bootstrap support 98%, decay index 4): *Brachyhypopomus gauderio*, *B. pinnicaudatus*.

Character 36, state 0 → 1 (unambiguous, homoplastic); *presence of foramen on coracoid*.

***Brachyhypopomus pinnicaudatus***

Character 26, state 1 → 0 (ambiguous, homoplastic); *presence of medial bridge on posterior portion of basihyal*.

Character 44, state 0 → 1 (unambiguous, non-homoplastic); *posttemporal and supracleithrum fused*.

***Brachyhypopomus gauderio***

No morphological autapomorphies.

***Brachyhypopomus* sp. FLAV**

Character 8, state 0 → 1 (unambiguous, non-homoplastic); *branch of infraorbital canal independent from frontal*.

Character 9, state 0 → 1 (unambiguous, homoplastic); *supraorbital canal independent from frontal*.

Character 19 , state 1 → 0 (unambiguous, homoplastic); *interhyal* *uneven, non-cone-like shape*.

Character 22, state 0 → 1 (unambiguous, homoplastic); *absence of first branchiostegal ray.*

Character 54, state 1 → 0 (unambiguous, homoplastic); *neural complex not contacting exoccipital*.

Character 58, state 0 → 1 (unambiguous, homoplastic); *anterior portion of neural complex concave*.

**Clade J (bennetti Species Group)**

(2 species; bootstrap support 100%, decay index 31): *Brachyhypopomus bennetti*, *B. walteri*.

Character 3, state 0 → 1 (unambiguous, homoplastic); *absence of antorbital*.

Character 35, state 0 → 1 (unambiguous, homoplastic); *anterior portion of first through third proximal radials in contact*.

Character 36, state 0 → 1 (unambiguous, homoplastic); *presence of foramen on coracoid*.

Character 42, state 0 → 1 (unambiguous, non-homoplastic); *presence of ascending process on coracoid*.

Character 56, state 0 → 1 (unambiguous, homoplastic); *ascending process on endopterygoid contacts orbitosphenoid.*

***Brachyhypopomus bennetti***

No morphological autapomorphies (although see diagnoses for differences in caudal-filament morphology not included in the morphological character matrix).

***Brachyhypopomus walteri***

No morphological autapomorphies (see comment above for *B. bennetti*).

**Clade 3**

(10 species; bootstrap support 74%, decay index 5): *Brachyhypopomus* sp. BATE, *B.* sp. BENJ, *B. bombilla,* *B. diazi*, *B.* sp. MENE, *B. occidentalis*, *B.* sp. PALE, *B.* sp. PROV, *B.* sp. REGA¸ *B.* sp. SULL.

Character 34, state 0 → 1 (ambiguous, homoplastic); t*hird and fourth proximal radials partially fused.*

Character 47, state 1 → 0 (ambiguous, homoplastic); *descending process of maxilla broad*.

Character 51, state 0 → 1 (unambiguous, homoplastic); *dorsoposterior portion of dentary uneven, or with hook-like process.*

Character 54, state 1 → 0 (unambiguous, homoplastic); *neural complex not contacting exoccipital.*

**Clade 4**

(9 species; bootstrap support <50%, decay index 1): *Brachyhypopomus* sp. BATE, *B.* sp. BENJ, *B. bombilla,* *B. diazi*, *B.* sp. MENE, *B. occidentalis*, *B.* sp. PROV, *B.* sp. REGA¸ *B.* sp. SULL.

No morphological synapomorphies.

***Brachyhypopomus* sp. PALE**

Character 24, state 0 → 1 (unambiguous, homoplastic); *first branchiostegal ray approximately half the length as the second branchiostegal ray.*

Character 48, state 0 → 1 (unambiguous, homoplastic); *preopercular sensory canals independent of preopercle.*

**Clade 5**

(7 species; bootstrap support 55%, decay index 3): *Brachyhypopomus* sp. BATE, *B.* sp. BENJ, *B. bombilla,* *B.* sp. MENE, *B.* sp. PROV, *B.* sp. REGA, *B.* sp. SULL.

Character 16, state 1 → 0 (unambiguous, homoplastic); *absence of lateral ethmoid*.

Character 19, state 0 → 1 (ambiguous, homoplastic); *interhal cone-like, posterior portion distinctly wider than the anterior portion*.

Character 34, state 1 → 0 (ambiguous, homoplastic); *third and fourth proximal radials clearly separated*.

Character 48, state 0 → 2 (ambiguous, homoplastic); *only the posterior-most sensory canal incised in preopercle*.

Character 56, 0 → 1 (unambiguous, homoplastic); *ascending process on endopterygoid contacting orbitosphenoid.*

**Clade 6**

(6 species; bootstrap support <50%, decay index 1): *Brachyhypopomus* sp. BATE, *B.* sp. BENJ, *B. bombilla,* *B.* sp. MENE, *B.* sp. PROV, *B.* sp. REGA.

Character 23, state 0 → 1 (ambiguous, homoplastic); *first branchiostegal ray distinctly narrower than third ray*.

Character 26, state 0 → 1 (ambiguous, homoplastic); *absence of medial bridge on posterior portion of basihyal*.

Character 33, state 0 → 1 (unambiguous, homoplastic); *absence of the fourth proximal radial*.

Character 51, state 1 → 0 (ambiguous, homoplastic); *dorsoposterior portion of dentary straight and even*.

***Brachyhypopomus* sp. SULL**

No morphological autapomorphies

**Clade K (sp. BATE Species Group)**

(3 species; bootstrap support 97%, decay index 23): *Brachyhypopomus* sp. BATE, *B.* sp. BENJ, *B.* sp. PROV*.*

Character 19, state 1 → 0 (ambiguous, homoplastic); *interhyal cone-like*.

Character 22, state 0 → 1 (unambiguous, homoplastic); *absence of first branchiostegal ray*.

Character 28, state 1 → 0 (ambiguous, homoplastic); *second basibranchial funnel-shaped*.

Character 31, state 0 → 1 (ambiguous, homoplastic); *absence of gill rakers*.

Character 45, state 0 → 1 (ambiguous, homoplastic); *scales absent on dorsal region of anterior 1/3 of body*.

Character 47, state 0 → 1 (unambiguous, homoplastic); *descending process of maxilla narrow in all ontogenetic stages*.

**Clade L**

(2 species; bootstrap support 97%, decay index 19): *Brachyhypopomus* sp. BATE, *B.* sp. BENJ*.*

No morphological synapomorphies

***Brachyhypopomus* sp. BATE**

Character 28, state 0 → 1 (ambiguous, homoplastic); *second basibranchial* *arrow-shaped*.

Character 31, state 1 → 0 (ambiguous, homoplastic); *presence of gill rakers*.

Character 45, state 1 → 0 (ambiguous, homoplastic); *presence of scales on dorsal region of anterior 1/3 of body*.

***Brachyhypopomus* sp. BENJ**

Character 21, state 0 → 1 (unambiguous, homoplastic); *tube-like interhyal narrow*.

Character 29, state 0 → 1 (unambiguous, homoplastic); *third basibranchial ossified*.

***Brachyhypopomus* sp. PROV**

Character 26, state 1 → 0 (ambiguous, homoplastic); *presence of medial bridge on posterior portion of basihyal.*

**Clade M (bombilla Species Group)**

(3 species; bootstrap support 91%, decay index 1): *Brachyhypopomus bombilla*, *B.* sp. MENE, *B.* sp. REGA.

Character 60, state 0 → 1 (unambiguous, non-homoplastic); *presence of accessory electric organ on opercular region*.

**Clade N**

(2 species; bootstrap support 77%, decay index 1): *Brachyhypopomus* sp. MENE, *B.* sp. REGA.

Character 24, state 0 → 1 (ambiguous, homoplastic); *first branchiostegal ray approximately half the length as the second branchiostegal ray*.

Character 25, state 1 → 0 (unambiguous, homoplastic); *basihyal smaller than first ceratohyal*.

Character 51, state 0 → 1 (ambiguous, homoplastic); *dorsoposterior portion of dentary uneven, or with hook-like process*.

***Brachyhypopomus* sp. MENE**

Character 22, state 0 → 1 (unambiguous, homoplastic); *absence of first branchiostegal ray*.

***Brachyhypopomus* sp. REGA**

Character 54, state 0 → 1 (unambiguous, homoplastic); *neural complex contacting exoccipital.*

***Brachyhypopomus bombilla***

No morphological autapomorphies

**Clade O (occidentalis Species Group)**

(2 species; bootstrap support 100%, decay index 16): *Brachyhypopomus diazi*, *B. occidentalis*.

No morphological synapomorphies.

***Brachyhypopomus diazi***

Character 23, state 0 → 1 (unambiguous, homoplastic); *first branchiostegal ray distinctly narrower than third ray.*

***Brachyhypopomus occidentalis***

Character 24, state 0 → 1 (unambiguous, homoplastic); *first branchiostegal ray approximately half the length as the second branchiostegal ray*.

**Clade 7**

(6 species; bootstrap support <50%, decay index 1): *Brachyhypopomus brevirostris*, *B. bullocki*, *B.* sp. CUNI, *B.* sp. HEND, *B. janeiroensis*, *B. jureiae*.

Character 22, state 0 → 1 (unambiguous, homoplastic); *absence of first branchiostegal ray*.

**Clade P (brevirostris Species Group)**

(4 species; bootstrap support 86%, decay index 7): *Brachyhypopomus brevirostris*, *B. bullocki*, *B.* sp. CUNI, *B.* sp. HEND*.*

Character 37, state 0 → 1 (unambiguous, homoplastic); *presence of mesocoracoid bridge.*

Character 43, state 0 → 1 (unambiguous, non-homoplastic); *ventral process of coracoid absent*.

Character 53, state 0 → 1 (unambiguous, non-homoplastic); *supraoccipital overlapping with a small un-named bone above supraocciptal*.

**Clade Q**

(3 species; bootstrap support 99%, decay index 21): *Brachyhypopomus bullocki*, *B.* sp. CUNI, *B.* sp. HEND*.*

Character 17, state 0 → 1 (unambiguous, homoplastic); *lateral ethmoid narrow and tube-shaped*.

Character 18, state 0 → 1 (ambiguous, homoplastic); *ventral portion of orbitosphenoid not contacting dorsal region of parasphenoid*.

Character 26, state 0 → 1 (ambiguous, homoplastic); *absence of medial bridge on posterior portion of basihyal*.

Character 33, state 0 → 1 (unambiguous, homoplastic); *absence of the fourth proximal radial*.

Character 35, state 0 → 1 (ambiguous, homoplastic); *anterior portion of first through third proximal radials in contact with each other in adults*.

Character 39, state 0 → 1 (ambiguous, homoplastic); *absence of mesocoracoid bridge process on scapula*.

Character 40, state 0 → 1 (ambiguous, homoplastic); *posterior tip of mesocoracoid bridge narrow – of approximately equal width to the medial portion*.

***Brachyhypopomus brevirostris***

Character 27, state 0 → 1 (unambiguous, non-homoplastic); *first basibranchial ossified*.

Character 30, state 0 → 1 (unambiguous, homoplastic); *third basibranchial unossified*.

**Clade R**

(2 species; bootstrap support 98%, decay index 15): *Brachyhypopomus bullocki*, *B.* sp. HEND*.*

Character 56, state 0 → 1 (unambiguous, homoplastic); *ascending process on endopterygoid contacting orbitosphenoid*

***Brachyhypopomus bullocki***

Character 18, state 1 → 0 (ambiguous, homoplastic); *ventral portion of orbitosphenoid contacting dorsal region of parasphenoid*.

Character 19, state 0 → 1 (unambiguous, homoplastic); *cone-like, posterior portion distinctly wider than the anterior portion*.

Character 25, state 1 → 0 (unambiguous, homoplastic); basihyal smaller than first ceratohyal.

Character 26, state 1 → 0 (ambiguous, homoplastic); *presence of medial bridge on posterior portion of basihyal*.

Character 35, state 1 → 0 (ambiguous, homoplastic); *anterior portion of first through third proximal radials separated in adults*.

Character 39, state 1 → 0 (ambiguous, homoplastic); *presence of mesocoracoid bridge process on scapula*.

Character 40, state 1 → 0 (ambiguous, homoplastic); *posterior tip of mesocoracoid bridge wide*.

***Brachyhypopomus* sp. HEND**

Character 38, state 0 → 1 (unambiguous, homoplastic); *mesocoracoid bridge contacting scapula*.

***Brachyhypopomus* sp. CUNI**

Character 21, state 0 → 1 (unambiguous, homoplastic); *tube-like interhyal narrow*.

**Clade S (janeiroensis Species Group)**

(2 species; bootstrap support 100%, decay index 16): *Brachyhypopomus janeiroensis*, *B. jureiae.*

Character 48, state 0 → 1 (unambiguous, homoplastic); *preopercular sensory canals independent of preopercle*.

***Brachyhypopomus janeiroensis***

No morphological autapomorphies.

***Brachyhypopomus jureiae***

Character 16, state 1 → 0 (unambiguous, homoplastic); *absence of lateral ethmoid*.

Character 34, state 0 → 1 (unambiguous, homoplastic); *third and fourth proximal radials partially fused*.
